# The long-term efficacy of tetracycline class antimicrobials as local adjuncts in the treatment of chronic periodontitis: a systematic review and meta-analysis

**DOI:** 10.3389/fdmed.2025.1658720

**Published:** 2025-09-26

**Authors:** Niroshani S. Soysa, Samadhi L. Jayakody, C. N. R. A. Alles

**Affiliations:** ^1^ Department of Oral Medicine and Periodontology, Faculty of Dental Sciences, University of Peradeniya, Peradeniya, Sri Lanka; ^2^ Department of Biochemistry, Faculty of Medicine, University of Peradeniya, Peradeniya, Sri Lanka

**Keywords:** chronic periodontitis, clinical attachment gain, meta-analysis, probing pocket depth, randomized controlled trial, smoking status, systematic review, tetracycline

## Abstract

**Introduction:**

This systematic review assesses the long-term efficacy of tetracycline-class local antimicrobials as adjuncts to scaling and root planing (SRP) in chronic periodontitis. It focuses on improvements in primary outcomes such as probing pocket depth (PPD) and clinical attachment level (CAL), with particular attention to differences in treatment outcomes between smokers and non-smokers. Moreover, the assessed secondary outcomes encompassed bleeding on probing (BOP), gingival index (GI), and plaque index (PI).

**Method:**

A systematic search of PubMed, Cochrane Central, Scopus, and Embase identified randomized controlled trials (RCTs) published up to 2024 with ≥6 months follow-up. Long-term efficacy of local tetracyclines was assessed from the selected studies. Meta-analysis calculated weighted mean differences (WMDs) and 95% CIs for the selected periodontal indices using R software. Meta-regression evaluated the impact of study design, assessment approach, treatment phase, and smoking status.

**Results:**

This systematic review included 52 RCTs assessing the efficacy of adjunctive locally delivered antimicrobials in periodontal therapy. Meta-analysis showed significant benefits in both medium-term (6–9 months) and long-term (12 +  months) outcomes. Medium-term results demonstrated significant PPD reduction (WMD 0.516 mm, 95% CI 0.413; 0.620, *P* = 0.0001) and CAL gain (WMD 0.336 mm, 95% CI 0.204; 0.467, *P* = 0.0001), while long-term studies showed sustained improvements (PPD: WMD 0.371 mm, 95% CI 0.181; 0.560, *P* = 0.0001; CAL: WMD 0.310 mm, 95% CI 0.240; 0.381, *P* = 0.0001). Tetracycline fibers showed the greatest medium-term PPD reduction (0.705 mm), followed by minocycline ointment (0.580 mm). Long-term follow-up also demonstrated significant improvements in BOP, PI, and GI (WMD = 0.1–0.3, *P* < 0.05). Subgroup analysis revealed non-smokers had greater PPD reduction (0.630 mm vs. 0.112 mm) and CAL gain (0.715 mm vs. 0.464 mm) than smokers (*P* < 0.05) in long-term. Meta-regression indicated study design influenced outcomes, with split-mouth designs showing significantly greater improvements [*β* = 0.422, 95% CI (0.231; 0.613), *P* = 0.0001].

**Discussion:**

Sustained-release tetracyclines with SRP improve long-term outcomes in chronic periodontitis. Non-smokers exhibit greater clinical gains, though smokers also benefit. These results support tailored adjunctive local tetracycline use to optimize outcomes across patient groups. Further large-scale, long-term RCTs are needed to confirm efficacy and refine delivery formulations.

**Conclusion:**

Locally delivered tetracycline-class antimicrobials significantly improve periodontal outcomes, with minocycline showing the most consistent benefits. These findings support the integration of tetracycline-class agents into treatment protocols, with special consideration for high-risk patients such as smokers. Future studies should emphasize cost-effectiveness, comparative efficacy, and long-term benefits, including the challenging management of furcation lesions, to better guide clinical decision-making and optimize patient outcomes.

## 1 Introduction

Periodontal diseases are considered one of the most prevalent non-communicable diseases globally, affecting approximately 19% of the world population (1). Among them, chronic periodontitis is characterized by progressive attachment loss, alveolar bone resorption, pocket formation, and gingival inflammation (2).

Scaling and Root Planing (SRP) remains the routine and established non-surgical therapy in managing patients diagnosed with periodontitis, among the other modalities which are encompassed in Subgingival Mechanical Debridement (SMD) (3). It is defined as a procedure involving the removal of dental plaque and calculus (scaling) and the smoothing of exposed root surfaces (root planing) to eliminate cementum or dentin impregnated with calculus, toxins, or microorganisms (4), thereby allowing regeneration of the lost attachment apparatus (5). SRP has been shown to reduce periodontal bacterial load and improve clinical parameters, including probing pocket depth (PPD), clinical attachment level (CAL), bleeding on probing (BOP), gingival index (GI), and plaque index (PI) (6, 7).

Despite its proven benefits, SRP presents several inherent limitations that preclude its use as a monotherapy in certain clinical situations. Studies have shown that SRP is less effective at mobile teeth, deep sites, and posterior teeth, particularly in molars with furcation involvements (8). Furthermore, routine SRP often results in hard tissue loss of the tooth structure, potentially causing root sensitivity (9). Most importantly, SRP alone may be insufficient to prevent bacterial invasion into tissues of deep pockets, leading to possible re-infection and persistent lesions, particularly at localized sites (10).

To overcome these limitations of SRP, various adjunctive therapies have been developed and investigated. These include antimicrobials, antiseptics, host-modulating agents, lasers, photodynamic therapy, and probiotics (11). Among them, due to the localized nature of persistent lesions and the bacterial etiology of chronic periodontitis, antimicrobials are considered among the most suitable adjunctive therapies (12).

Antimicrobials can be delivered systemically or locally, each with distinct characteristics. Systemic antimicrobials have demonstrated efficacy in both chronic and aggressive periodontitis (13, 14). However, it presents numerous disadvantages that limit its routine application. These include the requirement for good patient compliance, potential systemic adverse effects, increased risk of bacterial resistance, inability to attain adequate concentrations at pathological sites, and the need for high systemic doses (15, 16). Local drug delivery systems offer a compelling alternative, providing direct administration to the affected site, achieving high concentrations for prolonged periods, and minimizing systemic exposure (17). These systems, available as fibers, films, gels, pastes, ointments, and microspheres, exhibit superior pharmacokinetic properties and reduce the need for surgical intervention in deep pockets (18, 19). Numerous studies assessing the effectiveness of locally administered antimicrobials suggest that they may be as effective as SRP alone, indicating that adjunctive use could further enhance SRP's efficacy (20, 21).

Among local antimicrobials, the tetracycline class—comprising tetracycline, doxycycline, and minocycline—is widely utilized due to its broad-spectrum antimicrobial activity and additional therapeutic properties. Systemically, tetracyclines are bacteriostatic, but when delivered locally via controlled-release devices, they exert bactericidal effects against many anaerobic periodontal pathogens (22). Tetracyclines also demonstrate substantivity, adhering to dentine and cementum, which allows sustained drug release in the gingival crevicular fluid for 10–14 days. Beyond antimicrobial action, tetracyclines possess anti-collagenolytic, anti-inflammatory, and anti-resorptive effects, which mitigate connective tissue destruction and bone loss (23). Tetracyclines have also been shown to retard pellicle and plaque formation and pocket formation, contributing to their overall efficacy in periodontal therapy (24). Multiple studies have confirmed that locally administered tetracyclines improve clinical indices to a degree comparable to SRP alone, suggesting significant potential as adjuncts (25), but their long-term benefits, particularly in specific patient populations, require further exploration.

Tetracycline HCl (TET) has been extensively studied in various controlled and sustained-release delivery systems. Studies have demonstrated that TET alone can improve clinical indices such as PPD reduction, CAL gain, and changes in BOP, PI, and GI to levels comparable to SRP alone (26, 27). When used as an adjunct to SRP, TET has shown additional benefits in terms of clinical and microbiological outcomes (10, 28). Doxycycline is a semisynthetic tetracycline derivative that has demonstrated significant improvements in PPD reduction and CAL gain when used as an adjunct to SRP compared to SRP alone (18, 29). Minocycline has also demonstrated significant improvements in clinical parameters when used as an adjunct to SRP (30, 31).

Despite extensive research, several knowledge gaps persist. Previous systematic reviews have consistently reported that adjunctive antimicrobial therapy provides short-term improvements in periodontal parameters, but they also highlighted the lack of long-term follow-up data beyond 6–9 months. This limitation prevents firm conclusions regarding relapse or the sustained effect of these agents (32). Few systematic reviews and meta-analyses on locally delivered antibiotics have analyzed tetracycline separately, but subgroup analyses were generally limited to only a few medium-term studies (33) and limited long-term studies (34), with no evaluation by specific test products. Studies that did analyze test products, however, did not perform subgroup analyses according to tetracycline type (35). Reviews concentrating solely on tetracycline were often restricted to short-term (3 months) or medium-term (6 months) outcomes without subgroup analyses (36). In smoker populations, subgroup analyses were either absent (37) or restricted to a single formulation such as doxycycline (38), with some reviews excluding smoker-only studies while still including mixed-population studies without stratified analyses for smokers vs. non-smokers (35). Moreover, most studies have primarily focused on primary outcomes such as PPD and CAL, while secondary outcomes including BOP, PI, and GI have been underexplored (33, 36, 39). In addition, participant numbers for individual tetracycline formulations were often small, limiting the strength of conclusions about their efficacy. To compensate, many reviews pooled data across different antibiotic types, but this approach risks obscuring formulation-specific effects. These gaps underscore the need for adequately powered, long-term studies that evaluate individual tetracycline products, extend follow-up beyond 12 months, and stratify efficacy across clinically relevant subgroups, such as smokers and non-smokers.

Smoking is a major risk factor for periodontitis, associated with deeper pockets, more severe attachment loss, greater bone destruction, and higher rates of tooth loss (40). Smokers typically exhibit reduced healing responses following conventional periodontal therapy compared to non-smokers (41). Intriguingly, some evidence suggests smokers may derive greater benefits from local antibiotics, with studies reporting significantly greater PPD reductions and CAL gains when adjunctive local antimicrobials are used (37, 42). However, no comprehensive meta-analysis has specifically evaluated tetracycline-class local delivery devices in chronic periodontitis with long-term follow-up, stratified by smoking status.

This study aims to address the following Population, Intervention, Comparison, and Outcome (PICO) question: Do sustained and controlled-release local tetracycline delivery devices, used as adjuncts to SRP, improve clinical indices such as PPD and CAL in patients with chronic periodontitis compared to those treated with SRP alone? By focusing on long-term outcomes, this study seeks to fill a critical knowledge gap. Understanding the differential efficacy of local tetracycline therapy in groups such as smokers vs. non-smokers could inform personalized treatment strategies, optimizing outcomes for patients with varying risk profiles.

## 2 Methods

### 2.1 Literature search

**Figure 1:**
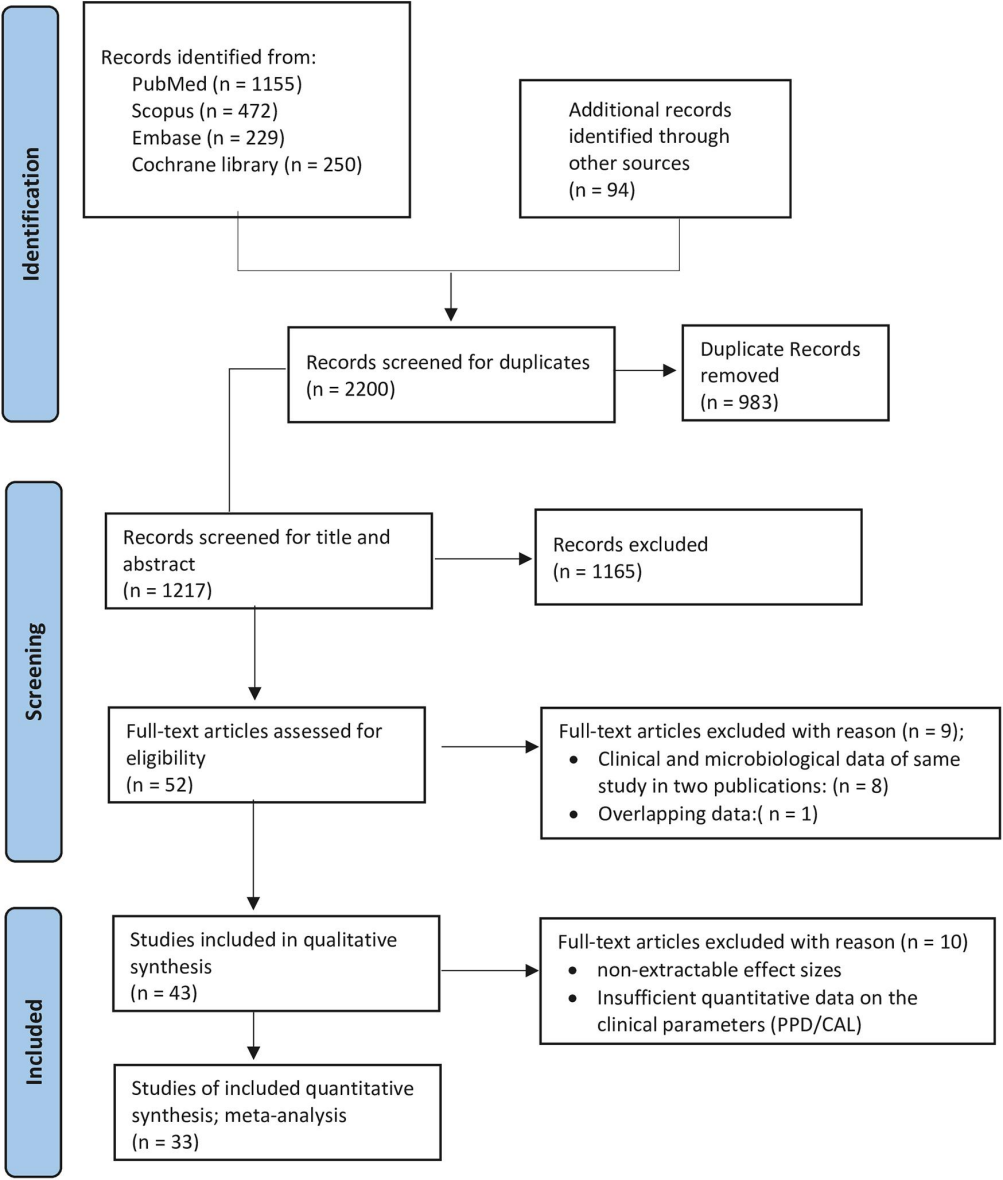
PRISMA flow diagram of the study selection process.

In order to identify potentially relevant human randomized controlled clinical trials published in English, a comprehensive systematic search was performed across several electronic databases, namely PubMed/MEDLINE, Cochrane Central Register of Controlled Trials, Scopus, and Embase. The search included publications up to December 2024 and utilized a mix of Medical Subject Headings (MeSH) and free-text keywords. The search strategy, initially designed for PubMed and adjusted for use in the other databases, combined terms as follows: (Periodontal Diseases OR periodontal disease OR periodontitis) AND (tetracycline OR doxycycline OR minocycline) AND (local OR topical) AND (randomized controlled trials OR clinical trials) (Supplementary Appendix 1). In addition, the reference lists of selected systematic reviews and individual studies were manually examined to locate any potentially relevant articles not captured by the electronic database. This systematic review was conducted in accordance with the PRISMA 2020 guidelines for systematic reviews and meta-analyses (Supplementary Appendix 2), and its protocol was registered in the PROSPERO database (Registration No: CRD42021247300) (Figure 1).

Although the redefinition of periodontal diagnoses (e.g., Stage/Grade) was established by the 2017 World Workshop on the Classification of Periodontal and Peri-Implant Diseases (43), a plethora of published literature from 1990 to 2024 labeled cases as “chronic periodontitis” and described interventions as “SRP”. Since, SRP remains the gold standard non-surgical therapy and therefore, in order to ensure comprehensiveness and consistency with the available RCT literature, we retained the terms “SRP” and “chronic periodontitis” in our search strategy and data inclusion criteria while acknowledging the updated classification framework.

### 2.2 Selection of studies

The inclusion criteria were defined according to the PICO framework:

Population (P): Human participants aged 18 years or older diagnosed with chronic or adult periodontitis.

Intervention (I): SRP (whether full-mouth, localized, or performed in single or repeated appointments) combined with the administration of a locally delivered tetracycline-based antibiotic (e.g., tetracycline, doxycycline, or minocycline) at manufacturer-recommended concentrations or dosages.

Comparison (C): SRP alone or combined with a vehicle/placebo.

Outcomes (O): Primary outcomes included changes in PPD and CAL. Secondary outcomes included BOP, PI, and GI, which provide additional information on inflammation and oral hygiene status.

Any adverse effects reported by patients or examiners were also collected when available. Only human randomized controlled clinical trials (RCTs) with either parallel or split-mouth designs were included. A minimum follow-up period of six months was required for inclusion. Studies were excluded if they involved systemic antimicrobials as an intervention, evaluated local antimicrobials as monotherapy, employed non–sustained-release delivery vehicles, or were conducted exclusively in diabetic populations.

### 2.3 Data abstraction and study characteristics

A systematic search of electronic databases was conducted to identify relevant studies. Two independent reviewers (NSS, SLJ) evaluated study eligibility by first screening titles and abstracts, followed by a detailed review of the full texts of potentially eligible studies. Studies lacking adequate data for meta-analysis were included in the systematic review but excluded from the meta-analysis. Data extraction was carried out using Microsoft Excel (Microsoft, Redmond, WA, USA), collecting the following details: author information (name and publication year), participant demographics, study design and characteristics, methods for clinical outcome assessment, intervention specifics (type and dosage of tetracycline used), changes in clinical indices, and any documented adverse effects.

### 2.4 Risk of bias assessment; in individual studies

Quality assessment was conducted independently by the same two reviewers. The risk of bias within individual randomized controlled trials was evaluated using the Cochrane Collaboration Risk of Bias Tool, which is widely regarded as the standard instrument for assessing methodological quality in intervention studies (44). This tool was chosen because it provides a structured framework to assess bias across key domains: selection bias (random sequence generation and allocation concealment), performance bias (blinding of participants and personnel), detection bias (blinding of outcome assessors), attrition bias (handling of incomplete outcome data), and reporting bias (selective outcome reporting). Each domain was rated as having a high (H), low (L), or unclear risk (UR) of bias (45).

### 2.5 Data analysis

Meta-analyses were performed by pooling the mean differences in treatment effects from baseline to follow-up. Data on primary outcomes from the selected studies were combined and evaluated using weighted mean differences (WMDs) accompanied by 95% confidence intervals (CIs). For both test and control groups, mean changes in clinical indices—such as probing pocket depth (PPD) and clinical attachment level (CAL)—were obtained from the studies. When these differences were not directly reported, they were derived from baseline and end-point values following methods outlined in prior literature (34, 35, 46). The difference (Δ) was computed using the equation:

$$\Delta PPD\;=PPD_{BL}-PPD_{End}$$

where PPDBL is the mean PPD at baseline, and PPDEnd is the mean PPD at the end of follow-up. This calculation was extended to other indices, including ΔCAL, ΔBOP, ΔPI, and ΔGI. Variances for these differences were estimated using,

$$Var\Delta \;=Var_{BL}+\;Var_{End}-2\times r\times \sigma_{BL}\times \sigma_{End}$$

where r (assumed 0.5) represents the correlation between baseline and follow-up measures, and *σ*BL and *σ*End are the standard deviations at baseline and end of follow-up, respectively (34, 35, 46). Heterogeneity among studies was quantified with the Cochrane *Q* test and *I*² statistic (25% = low, 50% = moderate, 75% = high), and effect estimates were combined under both fixed-effect/common-effect (Mantel–Haenszel–Peto) and random-effects (DerSimonian–Laird) models, reporting the latter as significant heterogeneity was present. Subsequently, subgroup analysis and meta-regression were performed. All computations were performed in R (version 4.4.2; R Core Team, 2024), with statistical significance defined at *P* ≤ 0.05.

### 2.6 Publication bias and sensitivity analyses

For assessment of publication bias across trials, funnel plot asymmetry was examined and formally tested using Egger's regression method (47) when at least ten studies were available. If bias was indicated, the trim-and-fill approach (48) was applied to estimate and impute potentially missing studies and derive an adjusted pooled effect size. Robustness of the findings was further evaluated through sensitivity analyses—by leave-one-study-out approach, in which each study was omitted in turn to assess its individual contribution to the overall effect.

## 3 Results

### 3.1 Study selection

The PRISMA (Preferred Reporting Items for Systematic Reviews and Meta-Analyses) study flow, which delineates the process of article inclusion, is shown in Figure 1. Publications released until 2024 that were in the English language underwent a screening process. Records were screened, and following an examination of the titles and abstracts of the identified articles, 52 articles were selected; of these, 43 articles were incorporated into the systematic review, while the remaining 9 were excluded after a thorough analysis of the full texts (Supplementary Appendix 3). For the meta-analysis, 33 articles met the eligibility criteria. Although 52 articles were reviewed for data extraction, they correspond to 43 independent studies due to the fact that clinical data and clinical data coupled with microbiological data were presented in two distinct publications: Cortelli et al. (49, 50); Drisko et al.; Michalowicz et al. (26, 51); Eickholz et al.; Ratka-Krüger et al. (21, 52); Goodson et al.; Socransky et al. (53, 54); Newman et al.; Wilson et al. (28, 55); Wong et al.; Wong et al. (56, 57). Thus, the publications that addressed the clinical data were utilized (21, 26, 28, 49, 53, 56). The investigations conducted by Tomasi et al. (58); Tomasi and Wennstrom (59) explored the same data from varying perspectives, with the former providing a comprehensive discussion and the latter examining the data in relation to different degrees of furcation involvement. Moreover, another study derived its data from three distinct papers: Colombo et al.; Gonçalves et al.; Rodrigues et al. (60–62), two of which primarily concentrated on microbiological data, while Gonçalves et al. (61) addressed clinical data. Nevertheless, the research by Rodrigues et al. (62) engaged in a subgroup analysis based on the smoking status of the cohort.

### 3.2 Study characteristics and quality assessment

#### 3.2.1 Study design and population

The characteristics of the studies included in this review are delineated in Tables 1, 2. Among the 43 studies selected for inclusion, 17 employed a split-mouth design, whereas 26 utilized a parallel design. Of the 43 studies, 31 were conducted in a single-center context, while 12 were executed in multi-centers (Table 1). The research settings predominantly occurred within university environments (*n* = 37) or private facilities (*n* = 3). Two studies failed to disclose the specific setting of the investigation (Supplementary Table 1). The duration of the studies ranged from a minimum of 6 months to a maximum of 24 months. Among the studies under consideration, 14 incorporated multiple test groups, while 6 included more than one control group (Supplementary Table 2).

**Table 1:** Demographics of the study population.

Study reference	Country	Centre (Single/ Multi)	Sample size (baseline/end)	Mean age (range)	Female (%)	Smokers (%)	Study duration (months)
Aboelsaad et al. (2014) (63)	Lebanon	Single	20/20	37 (31–49)	75	100	6
Ağan et al. (2006) (64)	Turkey	Single	10/NR	55 (41–69)	40	NR	6
Ahamed et al. (2013) (65)	India	Single	12/12	NR (22–55)	41.6	NR	6
Aimetti et al. (2004) (20)	Italy	Single	19/19	47 ± 10.78 (NR)	58	Non-smokers	6, 12
Bogren et al. (2008) (66)	USA & Sweden	Multi	128/124	NR (34–82)	58.6	29.7	12, 24
Cortelli et al. (2006) (49); Cortelli et al. (2008) (50)	Brazil	Single	59/26	46.8 ± 12.1 (26–69)	46.2	Non-smokers	6,9
Dannewitz et al. (2009) (67)	Germany	Single	39/34	NR	59	25.6	6, 12
Deo et al. (2011) (68)	India	Single	60/60	36.8 ± 4.87 (30–45)	58.3	Non-smokers	6
Drisko et al. (1995); Michalowics et al. (1995) (26, 51)	USA	Multi	122/116	45.1 (25–73)	44.3	59	12
Eickholz et al. (2002); Ratka-Krüger et al. (2005) (21, 52)	Germany, The Netherlands	Multi	111/110	49.9 ± 9.8 (23–71)	63	37	6
Flemmig et al. (1996) (69)	Germany	Single	35/28	53.6 ± 12.4 (NR)	35.7	NR	6
Friesen et al. (2002) (70)	USA	Single	28/24	43.6 (26–69)	53.6	NR	6
Colombo et al. (2003); Gonçalves et al. (2004); Rodrigues et al. (2004) (60–62)	Brazil	Single	30/NR	46 ± 11 (NR)	56.7	16.7	6
Goodson et al. (1985) (71)	USA	Single	10/10	38 (27–52)	70	NR	6, 9, 12
Goodson et al. (2012); Socransky et al. (2013) (53, 54)	USA, Sweden	Multi	231/187	48 ± 0.8 (>20)	NR	40.1	24
Gopinath et al. (2009) (72)	India	Single	15/15	35–50	NR	NR	6
Henderson et al. (2002) (73)	New Zealand	Single	15/15	46.3 (35–69)	53.3	40	6
Jain et al. (2012) (74)	India	Single	15/13	NR	NR	Non-smokers	6, 9
Jones et al. (1994) (75)	USA	Single	51/39	NR (28–68)	NR	NR	6
Killeen et al. (2016) (76)	USA	Single	60/51	67.0 (40–85)	31.3	23.5	6, 12
Kinane and Radvar (1999) (77)	UK	Single	83/79	45 ± 6.4	63.3	NR	6
Lie et al. (1998) (78)	Norway	Single	18/18	NR (36–77)	NR	NR	6
Machion et al. (2004) (79)	Brazil	Single	48/43	NR	55.8	100	6
Machion et al. (2006) (80)	Brazil	Single	48/30	NR	55.8	100	12, 18, 24
Meinberg et al. (2002) (81)	USA	Single	48/48	56 (NR)	54.2	31.3	12
Newman et al. (1994); Wilson et al. (1997) (28, 55)	USA	Multi	113/105	51.0 (NR)	46.7	NR	6
OPI (103A) (2000) (82)	USA	Multi	368	NR	44.8	41	9
OPI (103B) (2000) (83)	USA	Multi	380	NR	45.5	31.6	9
Oringer et al. (2002) (84)	USA	Multi	271	>30	NR	100	6, 9
Reddy et al. (2016) (85)	India	Single	53/48	NR	NR	Non-smokers	6, 12
Singh et al. (2014) (86)	India	Single	41/35	20–50	51.4	NR	6
Soeroso et al. (2017) (87)	Indonesia	Single	84/81	30–55	77.8	Non-smokers	6
Sweatha et al. (2015) (88)	India	Single	18/NR	>30	NR	NR	6
Tabenski et al. (2017) (89)	Germany	Single	54/45	48–63	53.3	10	6, 12
Timmerman et al. (1996) (90)	The Netherlands	Single	20/20	44.9 (39–59)	65	NR	6, 9, 12,15, 18
Tomasi et al. (2008); Tomasi and Wennstrom (2011) (58, 59)	Sweden	Single	33/32	NR (32–70)	53.1	46.9	9
Tonetti et al. (1998) (91)	Italy	Multi	127/123	49.7 ± 9.2 (NR)	44.8	45.6	6
Tonetti et al. (2012) (92)	Italy, Belgium, Germany, Greece, The Netherlands, Switzerland	Multi	202/200	NR	60.4	NR	6, 12
Van Dyke et al. (2002) (93)	USA	Single	50/44	NR	NR	NR	6
Van Steenberghe et al. (1999) (94)	Belgium, Sweden, UK, The Netherlands	Multi	104/93	46 (34–64)	50	NR	6, 9, 12, 15
Williams et al. (2001 (31)	USA	Multi	748/696	NR (29–79)	45.2	36.2	9
Wong et al. (1998); Wong et al. (1999) (56, 57)	Taiwan	Single	30/30	42.7 (NR)	36.7	NR	6
Zingale et al. (2012) (95)	USA	Single	25/25	50.9 (31–76)	52	4	6

NR, not reported.

**Table 2:** Study characteristics used for the meta-analysis.

Study reference	Blinding	Design	Phase; Initial/Maintenance	Assessment; FM/PM	Test product
Aboelsaad et al. (2014) (63)	Single	Split	Initial	PM—2 sites	Arestin
Ağan et al. (2006) (64)	Single	Split	Initial	PM—2 sites	Atridox
Ahamed et al. (2013) (65)	NR	Parallel	Initial	PM—5 sites	Atridox
Aimetti et al. (2004) (20)	Single	Split	Maintenance	PM—2 teeth	Actisite
Bogren et al. (2008) (66)	Single	Parallel	Maintenance	FM—PD > 4	Atridox
Cortelli et al. (2006) (49); Cortelli et al. (2008) (50)	Double	Parallel	Initial	PM—2 sites	Arestin
Dannewitz et al. (2009) (67)	Single	Parallel	Maintenance	PM—all furcation lesions	Ligosan
Deo et al. (2011) (68)	Single	Parallel	Initial	PM—6 sites	Atridox
Drisko et al. (1995) (26)	Single	Split	Initial and Maintenance	FM	Actisite
Eickholz et al. (2002); Ratka-Krüger et al. (2005) (21, 52)	Double	Split	Initial and Maintenance	PM—4–6 site	Ligosan
Flemmig et al. (1996) (69)	Single	Split	Maintenance	PM—1 tooth 6 sites/tooth	Actisite
Friesen et al. (2002) (70)	Single	Split	Initial	PM—1 tooth	Tetra strip
Colombo et al. (2003); Gonçalves et al. (2004); Rodrigues et al. (2004) (60–62)	Single	Parallel	Initial	FM/PM—4 sites	Actisite
Goodson et al. (1985) (71)	Single	Split	Initial	FM	Actisite
Goodson et al. (2012); Socransky et al. (2013) (53, 54)	Single	Parallel	Initial	FM	Actisite
Gopinath et al. (2009) (72)	NR	Split	Initial	PM—4 sites 2–6 teeth	Arestin
Henderson et al. (2002) (73)	Single	Split	Initial	PM—1 site	Arestin
Jain et al. (2012) (74)	NR	Split	Initial	PM—1–2 sites	Dentomycin
Jones et al. (1994) (75)	Double	Parallel	Initial	PM—13 site (average)	Minocin (mino. Powder)
Killeen et al. (2016) (76)	Single	Parallel	Maintenance	PM—1 site	Arestin
Kinane and Radvar (1999) (77)	Single	Parallel	Maintenance	PM—1 site	Dentomycin and Actisite
Lie et al. (1998) (78)	Single	Split	Initial	PM—1 site	Aureomycin
Machion et al. (2004) (79)	Single	Parallel	Initial	PM—6 site	Atridox
Machion et al. (2006) (80)	Single	Parallel	Maintenance	PM—6 site	Atridox
Meinberg et al. (2002) (81)	Single	Parallel	Maintenance	PM—2 sites	Arestin
Newman et al. (1994); Wilson et al. (1997) (28, 55)	Single	Split	Maintenance	PM—2sites	Actisite
OPI (103A) (2000) (82)	Single	Parallel	Initial	FM-PD≥5mm	Arestin
OPI (103B) (2000) (83)	Single	Parallel	Initial	FM-PD≥5mm	Arestin
Oringer et al. (2002) (84)	Single	Parallel	Initial	PM	Arestin
Reddy et al. (2016) (85)	NR	Parallel	Maintenance	PM—1 site	Perio Col-TC
Singh et al. (2014) (86)	NR	Split	Initial	FM	Periodontal Plus AB
Soeroso et al. (2017) (87)	Open blind	Parallel	Initial	FM	Periocline
Sweatha et al. (2015) (88)	Single	Split	Initial	PM—4	Arestin
Tabenski et al. (2017) (89)	Single	Parallel	Initial	PM—4 teeth	Arestin
Timmerman et al. (1996) (90)	Double	Parallel	Initial	FM/PM—4–10 sites	Dentomycin
Tomasi et al. (2008); Tomasi and Wennstrom (2011) (58, 59)	Single	Parallel	Initial	FM/PM—all furcation lesions	Atridox
Tonetti et al. (1998) (91)	Single	Parallel	Maintenance	PM—1 furcation lesion	Actisite
Tonetti et al. (2012) (92)	Single	Parallel	Maintenance	FM—PD >3	Ligosan
Van Dyke et al. (2002) (93)	Double	Parallel	Initial	PM—2 teeth	Arestin
Van Steenberghe et al. (1999) (94)	Double	Parallel	Initial	PM—6 sites	Mino ointment
Williams et al. (2001) (31)	Single	Parallel	Initial	FM—PD >4	Arestin
Wong et al. (1998); Wong et al. (1999) (56, 57)	NR	Split	Maintenance	PM—1–2 sites	Actisite
Zingale et al. (2012) (95)	Double	Split	Maintenance	PM—1 Site	Arestin

NR, not recorded; FM, full-mouth assessment; PM, partial-mouth assessment; PD, probing pocket depth.

The sample sizes of the studies included in this analysis exhibited considerable variability, with certain studies reporting sample sizes exceeding 100 (*n* = 10), all of which were multi-center investigations, and a few studies presenting sample sizes as low as 10 (*n* = 2), alongside others falling within intermediate ranges (Table 1). The majority of the studies provided data regarding the mean age and the age range of their respective cohorts, either for the aggregate group or based on the delineation of test/control groups. The percentage of female participants in the respective studies varied considerably, ranging from 31.3% to 77.8%. In terms of smoking status, 6 studies focused exclusively on non-smokers, 16 included both smokers and non-smokers, while 4 studies were conducted solely with smokers. The proportion of smokers in the mixed group varied from 4% to 59%. Notably, seventeen studies did not report the smoking status of their cohorts (Table 1).

#### 3.2.2 Patient recruitment, disease definition and criteria used to select treatment

The periodontal status of patients across different studies and the type of treatment administered (or not administered) prior to inclusion are presented in Supplementary Table 3. The majority of the research has classified the disease condition as moderate to severe chronic periodontitis (*n* = 31), whereas a few described it only as chronic periodontitis or adult periodontitis (Supplementary Table 4). Twenty-seven studies involved patients with initial disease, while 14 studies focused on maintenance patients (Supplementary Table 1). Additionally, two studies have utilized both initial and maintenance patients. The majority of the research incorporated in this systematic review uses PPD ≥ 5 mm and BOP as criteria for assessing patients/sites, while merely four studies have considered sites with PPD of ≥4 mm (Supplementary Table 4). Certain research has additionally employed different criteria, such as clinical attachment loss, bleeding on probing (BOP), and bone loss as alternative inclusion factors. Three studies focused solely on lesions with furcation involvement, while four studies explicitly stated that they did not include lesions exhibiting furcation involvement (Supplementary Table 4).

#### 3.2.3 Assessment of clinical parameters

Ten investigations have assessed the full mouth (FM) to evaluate changes in clinical indices either by considering values from all sites or based on a clinical criterion (e.g., PPD >4 mm), while 30 studies adopted a partial (PM) approach where most utilized a limited number of sites ranging from 1 to 10, and 3 studies focused on furcation involved lesions (58, 59, 67) (Supplementary Table 4). Three studies have utilized the FM and PM method (58, 61, 90). Twenty-two studies assessed 6 sites for each tooth, 4 studies examined 4 sites per tooth, and 9 studies evaluated 1–3 sites per tooth. Conversely, 7 studies did not provide information on that (Supplementary Table 5). Concerning the count of examiners in clinical evaluations, 15 studies utilized a single examiner, while 16 studies employed two or more examiners, and 12 studies did not disclose the number of examiners involved (Supplementary Table 5). Twenty studies have trained their examiners, while 23 did not report on this. The research has utilized different kinds of probes from the first, second, and third generations to assess clinical indices. A total of thirty-one studies employed manual probes like the Williams (*n* = 2) and UNC-15 (*n* = 13), along with the Florida probe, recognized as a force-controlled and computer-assisted option (*n* = 9); however, 5 studies did not disclose the type of probe utilized. Ten studies utilized stents, while seven studies conducted duplicate measurements, and two studies employed both (Supplementary Table 5). Five studies employed a site-based method, whereas 28 utilized a patient-based approach for data analysis. Two studies utilized both site and subject as the statistical unit, while 8 others did not provide that information (Supplementary Table 5). Aside from clinical outcomes, 15 studies conducted microbiological analysis, while 5 studies examined biomarkers. Additional effects assessed encompassed both radiological and systemic results. Different approaches have been utilized to assess PI, GI, and BOP, with the most prevalent being the techniques developed by Silness and Löe; Löe and Silness (96, 97) for measuring PI and GI, respectively (Supplementary Table 6). BOP was predominantly recorded either dichotomously (presence/absence), by the Sulcus Bleeding Index of Mühlemann and Son (98), or by the Papillary Bleeding Index of Mühlemann (99). To ensure a consistent basis for our synthesis, only those studies employing the papillary bleeding index were included in the meta-analysis (Supplementary Table 6).

#### 3.2.4 Pre-intervention measures

The individuals participating in the studies were either initial patients or those in maintenance with chronic periodontitis. Patients in maintenance received supportive periodontal therapy every 3–6 months. Consequently, the patients in most studies did not receive any periodontal treatment in the past 3–6 months. Following the recruitment of patients, the majority of the studies provided oral hygiene instructions (OHI) (*n* = 31), which were reinforced during later patient appointments. Numerous studies have provided either full mouth SRP (FMSRP) to patients (*n* = 2) or supragingival mechanical plaque removal (*n* = 8), while other research has administered Supportive Periodontal Therapy (SPT) at various time points (*n* = 4) (Supplementary Table 3). Additionally, 10 studies failed to disclose any interventions prior to the application of test products. One study has explicitly noted that they did not provide OHI (71). A limited number of studies have offered specific guidelines, such as refraining from brushing the treated area (*n* = 14) and avoiding hard and sticky foods from the treated sites (*n* = 5). Three studies have explicitly stated that patients were instructed to avoid mouthwashes or irrigation devices (65, 73, 94), while other studies have recommended that patients utilize 0.1%–0.2% chlorhexidine mouthwash for a duration ranging from 5 days to 1 month (*n* = 8) (Supplementary Table 3).

#### 3.2.5 Mechanical debridement by SRP

Prior to the adjunct application, 22 studies conducted FMSRP, while 19 studies performed SRP on localized sites, and 2 studies did not specify the type of SRP administered (Supplementary Table 3). Fourteen studies have provided local anesthesia prior to mechanical debridement, while 6 studies offered it if patients requested. One research explicitly stated that local anesthesia was not utilized (81), whereas the other studies did not mention its use (*n* = 22) (Supplementary Table 7). The majority of the research involved one operator conducting the mechanical debridement (*n* = 13), while six studies utilized 2–3 operators, and in other studies, the number of operators varied based on the number of centers (*n* = 4). Limited research has indicated the type of operator being either a dentist/periodontist (*n* = 6) or a dental hygienist (*n* = 4), while the majority of studies failed to disclose that information (*n* = 32). A few studies reported the duration of debridement, which was 1–1.5 h for full-mouth debridement (*n* = 2), 15–45 min for a quadrant (*n* = 3), and 5–10 min per tooth (*n* = 7). Eight studies conducted debridement without any time limitations, and 23 studies did not report the time limit. Nine studies have conducted SRP utilizing both hand and ultrasonic scalers, while four conducted hand scaling exclusively, and three performed SRP using only ultrasonic instruments (Supplementary Table 7).

#### 3.2.6 Application of test product and control groups

The current systematic review incorporated studies that utilized three varieties of tetracyclines: tetracycline, doxycycline, and minocycline, each administered in various formulations (Table 2). Research that incorporated tetracycline as an adjunct included Actisite (*n* = 10), Periodontal Plus AB (*n* = 1), and PerioCol-TC (*n* = 1), all offered in fiber form, while Aureomycin (*n* = 1) was available as an ointment. Additionally, research has utilized tetracycline strips where Doxycycline was administered in gel form in Atridox (*n* = 7) and Ligosan (*n* = 3). Research that employed minocycline was found in Arestin (*n* = 14), Dentomycin (*n* = 3), Periocline (*n* = 1), and Minocin (*n* = 1). Minocycline ointment (*n* = 1) has also been utilized by certain researchers which was not available in the market (Supplementary Table 2). In four studies, the agent was administered prior to an SRP procedure: one study (91) administered the agent on the day of fiber placement before the SRP, while three studies (56, 57, 69) administered the agent one week after the initial SRP. In contrast, all other studies (*n* = 39) administered the test agent at baseline following the SRP procedure (Supplementary Table 8).

Regardless of the agent, the majority of studies have utilized the agent just once (*n* = 29), while a small number have used it 2–4 times (*n* = 12), and two studies have employed the agent seven times (90, 94). In 12 studies, a dressing was used to avoid the dislodgement of the test product or to prevent spill-over, utilizing cyanoacrylate or a periodontal dressing for 3–13 days, with 9 studies documenting the dislodgement records (Supplementary Table 8). Concerning the control groups, most of the studies utilized SRP alone (*n* = 37), while five studies applied either SRP + vehicle or SRP + placebo. In comparison, three studies have utilized two controls: SRP only and SRP combined with a vehicle. Notably, a study that employed SRP exclusively included 2 control sites, with one adjacent to the test sites and another positioned in a different quadrant, considered as remote sites (73). Seven studies (*n* = 7) utilized an untreated group as a control, while two studies included placebo control groups (Supplementary Table 2).

#### 3.2.7 Quality assessment of the studies

Quality criteria for most studies were either unmet or unclear, indicating a high risk of bias (Supplementary Table 9). Random sequence generation (selection bias) was judged at low risk of bias in 14 studies (21, 26, 28, 52, 55, 58–62, 66, 67, 70, 71, 82, 83, 89, 92, 94) because of the use of a computer-generated random sequence prepared by an independent researcher, distinct from recruitment and treatment. While 28 studies (20, 31, 49, 53, 54, 56, 57, 63–65, 68, 69, 73–81, 84–88, 90, 91, 93, 95) were unclear, one study (72) was high as the randomization performed by the treating clinician in a way that could be foreseen.

Allocation concealment (selection bias) was low risk in 5 studies (21, 52, 58, 59, 92, 94) because of sequentially numbered, opaque, sealed envelopes and visually indistinguishable devices prepared off-site or by a third party, and high in 15 studies (20, 49, 56, 57, 65, 68, 72, 74–76, 87–90, 93) because of open lists. Twenty-three studies were unclear on how they concealed allocation (20, 26, 31, 49, 53, 54, 60–64, 66, 69–71, 73, 77–81, 84, 95).

Performance bias (blinding of participants and personnel) was low in 11 studies (21, 26, 49, 52, 67, 73, 75, 76, 90, 93–95) because of a double-blind setup and documented blinding procedure, and high in 5 (64, 72, 76, 87, 88) studies due to the absence of blinding. Meanwhile, 27 studies were unclear about the blinding procedure (20, 53, 54, 56, 57, 60–66, 69–72, 74, 77–81, 84–88).

Detection bias (blinding of outcome assessment) was low risk in 12 studies due to factors such as clinical outcomes (PPD, CAL) measured by an examiner blinded to allocation, controlled probing force, or use of electronic probes; reporting examiner calibration, and consistent measurement protocols and time points (20, 26, 31, 49, 53, 54, 63, 66, 78, 82, 83, 91, 95), and 3 were high due to unblinded outcome assessment (63, 72, 88). The remaining 28 studies were unclear on detection bias (20, 21, 52, 56, 57, 60–62, 64, 65, 67–77, 79–81, 84–87, 90).

Attrition bias (incomplete outcome data) was judged low risk in 14 studies, typically where attrition was balanced between groups, reasons for dropout were reported, and appropriate handling of missing data was conducted (20, 21, 52–54, 56, 57, 65, 68, 70, 73, 82, 83, 86, 87, 90, 92). It was unclear in 25 studies because studies did not describe how missing data were handled (26, 49, 60–67, 69, 71, 72, 74, 76–81, 84, 85, 88, 91, 95), and high in 4 (20, 72, 75, 87).

Reporting bias (selective reporting) was low in 17 studies that reported all pre-specified outcomes (PPD/CAL as primary; BOP/PI/GI and adverse events as secondary) at stated time-points with complete summary statistics (means and variance) and adverse events (21, 26, 31, 52, 56–63, 67, 70, 76, 77, 81–83, 89, 93, 94). It was unclear in 24 studies where no protocol/registration was available, the pre-specification of outcomes/time-points was not explicit, or results were presented partially unclear (20, 49, 53, 54, 64–66, 69, 71–75, 78–80, 84–88, 90, 91, 95). Two studies were high risk, showing clear discordance between Methods and Results—omitting pre-specified primary outcomes or time-points (72, 89).

#### 3.2.8 Outcomes with significant differences of studies

Of the 43 studies, 21 indicated a statistically significant reduction in PPD, 14 revealed a notable difference in CAL gain, and 5 showed a significant reduction in BOP. Moreover, 4 and 2 studies showed a significant difference in the decrease of GI and PI, respectively. Conversely, 18 studies failed to show any significant difference (Supplementary Table 10).

#### 3.2.9 Occurrence of adverse effects

Only a few studies reported adverse effects with the use of local antimicrobials. They included gingival redness, gingival tingling, headache, rhinitis, inflammation, periodontal abscesses, root sensitivity, caries, taste disturbances, tongue pigmentation, gingivitis, and stomatitis (Supplementary Table 10).

### 3.3 Meta-analysis—efficacy of the tested adjunctive local antimicrobials

#### 3.3.1 Meta-analysis results by primary and secondary outcomes

Results are presented separately for each parameter and timeframe, focusing on significant effects of study design (split-mouth vs. parallel), population (initial vs. maintenance phase), assessment type (full-mouth vs. partial-mouth), and smoking status (smokers vs. non-smokers). The findings highlight the influence of these moderators on treatment outcomes.

##### 3.3.1.1 Probing pocket depth (PPD)

For medium-term studies (6–9 months), a meta-analysis of 42 comparisons involving 1,995 control and 2,040 test patients revealed a significant PPD reduction favoring test groups [WMD = 0.516, 95% CI (0.413; 0.620), *P* = 0.0001], with substantial heterogeneity (*I*² = 72.39%, *P* = 0.0001) (Table 3; Supplementary Figure 1A). Significant differences (*p* < 0.05) were observed across study designs, with split-mouth studies showing a larger effect [*n* = 13, WMD = 0.819, 95% CI (0.622; 1.016), *P* = 0.0001] than parallel studies [*n* = 29, WMD = 0.391, 95% CI (0.303; 0.480), *P* = 0.0001]. Partial-mouth assessments yielded a higher WMD [*n* = 32, 0.573, 95% CI (0.469; 0.677), *P* = 0.0001] than full-mouth assessments [*n* = 10, WMD = 0.433, 95% CI (0.184; 0.682), *P* = 0.0007]. Patients in both initial and maintenance phase of treatment showed similar effect on the PPD reduction, where initial phase showed a reduction of 0.524 mm [*n* = 31, 95% CI (0.393; 0.655), *P* = 0.0001] and maintenance phase with a reduction of 0.559 mm [*n* = 9, 95% CI (0.393; 0.725), *P* = 0.0001]. Non-smokers showed significant effects [*n* = 7, WMD = 0.694, 95% CI (0.472; 0.917), *P* = 0.0001] similar to smokers [*n* = 2, WMD = 0.709, 95% CI (0.205; 1.213), *P* = 0.0058] (Table 3).

**Table 3:** Meta-analyses and meta-regression for probing pocket depth (PPD) changes.

(a) Medium-term Studies (6–9 months)									
	Number of			Weighted mean difference (WMD)				Heterogeneity	
		Patients			95% CI				
6–9 months	Studies	Control	Test	WMD	Lower	Upper	*p*-value	I2 (%)	*p*-value
All	42	1,995	2,040	0.516	0.413	0.62	0.0001	72.39%	0.0001
Parallel	29	1,478	1,523	0.391	0.303	0.48	0.0001	67.05%	0.0001
Split	13	517	517	0.819	0.622	1.016	0.0001	50.13%	0.0199
FM	10	1,058	1,095	0.433	0.184	0.682	0.0007	79.50%	0.0001
PM	32	937	945	0.573	0.469	0.677	0.0001	49.01%	0.0011
Initial	31	1,462	1,511	0.524	0.393	0.655	0.0001	77.93%	0.0001
Maintenance	9	317	313	0.559	0.393	0.725	0.0001	2.28%	0.4150
SM	2	41	42	0.709	0.205	1.213	0.0058	71.89%	0.0593
NS	7	117	117	0.694	0.472	0.917	0.0001	0.00%	0.4690
Type of tetracycline									
Tetracycline	8	303	305	0.740	0.401	1.079	0.0001	68.25%	0.0025
Doxycycline	7	306	309	0.454	0.209	0.698	0.0003	48.07%	0.0727
Minocycline	27	1,386	1,426	0.460	0.354	0.565	0.0001	73.39%	0.0001
Test product									
Actisite	5	234	236	0.705	0.503	0.907	0.0001	0.00%	0.9520
Arestin	17	1,154	1,190	0.406	0.279	0.534	0.0001	63.20%	0.0002
Atridox	4	70	77	0.446	0.029	0.862	0.0359	65.54%	0.0335
Dentomycin	5	66	67	0.581	0.265	0.896	0.0003	0.00%	0.5280
Ligosan	3	236	232	0.518	0.251	0.784	0.0001	3.90%	0.3530
Mino ointment	4	160	158	0.580	0.345	0.814	0.0001	86.74%	0.0001
Meta-regression									
				95% CI					
Moderator		*β* coefficient		Lower		Upper		*p*-value	
Split/parallel-mouth		0.422		0.231		0.613		0.0001	
Full/partial-mouth		0.182		−0.021		0.384		0.0795	
Initial/maintenance		−0.004		−0.282		0.274		0.9760	
Smoking/non-smoking		0.068		−0.355		0.490		0.7530	
(b) Long-term studies (12 + months)									
	Number of			Weighted mean difference (WMD)				Heterogeneity	
		Patients			95% CI				
12 + months	Studies	Control	Test	WMD		Lower	Upper	*p*-value	I2 (%)
All	21	530	535	0.371	0.181	0.56	0.0001	74.43%	0.0001
Parallel	20	511	516	0.348	0.153	0.542	0.0005	75.29%	0.0001
Split	1	19	19	0.850	0.245	1.455	0.0059	N/A	NA
FM	3	151	148	0.034	−0.143	0.21	0.7080	0.00%	0.5740
PM	18	379	387	0.444	0.236	0.651	0.0001	64.39%	0.0001
Initial	14	297	315	0.498	0.272	0.723	0.0001	63.09%	0.0008
Maintenance	7	233	220	0.179	−0.082	0.44	0.1800	62.53%	0.0137
SM	4	55	70	0.112	−0.193	0.418	0.4720	0.00%	0.4420
NS	4	61	61	0.630	0.145	1.115	0.0109	18.93%	0.2960
Type of tetracycline									
Tetracycline	3	58	61	0.427	−0.007	0.860	0.0538	34.05%	0.2200
Doxycycline	8	226	231	0.143	−0.058	0.344	0.1620	38.27%	0.1240
Minocycline	10	246	243	0.580	0.283	0.876	0.0001	72.79%	0.0001
Test Product									
Actisite	2	42	45	0.554	0.017	1.091	0.0434	44.74%	0.1790
Arestin	3	53	50	0.359	−0.497	1.216	0.4110	69.47%	0.0378
Atridox	7	207	216	0.141	−0.072	0.353	0.1950	47.06%	0.0786
Dentomycin	3	30	30	0.144	−0.635	0.922	0.7180	0.00%	0.9860
Mino ointment	4	163	163	0.745	0.496	0.994	0.0001	85.15%	0.0002
Meta-regression									
				95% CI					
Moderator		*β* coefficient		Lower		Upper		*p*-value	
Split/parallel-mouth		0.502		−0.412		1.416		0.2810	
Full/partial-mouth		0.379		−0.054		0.811		0.0859	
Initial/maintenance		−0.322		−0.674		0.030		0.0729	
Smoking/non-smoking		−0.531		−1.065		0.003		0.0513	

FM, full-mouth assessment; PM, partial-mouth assessment; SM, smokers; NS, non-smokers.

In long-term studies (12 + months), 21 comparisons with 530 control and 535 test patients showed a significant PPD reduction [WMD = 0.371, 95% CI (0.181; 0.560), *P* = 0.0001], with high heterogeneity (*I*² = 74.43%, *P* = 0.0001) (Table 3; Supplementary Figure 1B). Significant effects were noted for parallel-mouth design [*n* = 20, WMD = 0.348, 95% CI (0.153; 0.542), *P* = 0.0001], partial-mouth assessments [*n* = 18, WMD = 0.444, 95% CI (0.236; 0.651), *P* = 0.0001], initial phase [*n* = 14, WMD = 0.498, 95% CI (0.272; 0.723), *P* = 0.0001], and non-smokers [*n* = 4, WMD = 0.630, 95% CI (0.145; 1.115), *P* = 0.0109] (Table 3).

##### 3.3.1.2 Clinical attachment level (CAL)

In medium-term studies (6–9 months), 35 comparisons with 1,425 control and 1,448 test patients demonstrated a significant CAL gain [WMD = 0.336, 95% CI (0.204; 0.467), *P* = 0.0001], with high heterogeneity (*I*² = 79.21%, *P* = 0.0001) (Table 4; Supplementary Figure 2A). Comparatively higher significant effects were observed for split-mouth [*n* = 13, WMD = 0.564, 95% CI (0.314; 0.814), *P* = 0.0001], partial-mouth assessments [*n* = 30, WMD = 0.329, 95% CI (0.199; 0.459), *P* = 0.0015], and initial phase [*n* = 24, WMD = 0.367, 95% CI (0.192; 0.542), *P* = 0.0001] (Table 4). Almost similar values were observed for smokers [*n* = 2, WMD = 0.710, 95% CI (0.463; 0.957), *P* = 0.0001] and non-smokers [*n* = 4, WMD = 0.734, 95% CI (0.401; 1.066), *P* = 0.0001] (Table 4).

**Table 4:** Meta-analyses and meta-regression for clinical attachment level (CAL) changes.

(a) Medium-term Studies (6–9 months)									
	Number of			Weighted mean difference (WMD)				Heterogeneity	
		Patients			95% CI				
6–9 months	Studies	Control	Test	WMD	Lower	Upper	*p*-value	I2 (%)	*p*-value
All	35	1,425	1,448	0.336	0.204	0.467	0.0001	79.21%	0.0001
Parallel	22	888	911	0.215	0.083	0.348	0.0015	81.50%	0.0001
Split	13	537	537	0.564	0.314	0.814	0.0001	61.35%	0.0019
FM	5	494	509	0.396	−0.18	0.973	0.1780	88.64%	0.0001
PM	30	931	939	0.329	0.199	0.459	0.0001	76.84%	0.0001
Initial	24	892	919	0.367	0.192	0.542	0.0001	85.21%	0.0001
Maintenance	9	317	313	0.222	0.048	0.395	0.0123	0.00%	0.5250
SM	2	41	42	0.710	0.463	0.957	0.0001	0.00%	0.5820
NS	4	78	78	0.734	0.401	1.066	0.0001	0.00%	0.6140
Type of tetracycline									
Tetracycline	10	351	353	0.460	0.114	0.806	0.0092	72.86%	0.0001
Doxycycline	7	306	309	0.464	0.191	0.738	0.0009	35.80%	0.1550
Minocycline	18	768	786	0.241	0.090	0.392	0.0017	85.13%	0.0001
Test Product									
Actisite	5	234	236	0.264	0.02	0.509	0.0343	24.48%	0.2580
Arestin	9	549	563	0.298	0.107	0.488	0.0023	63.60%	0.0050
Atridox	4	70	77	0.495	0.061	0.928	0.0253	67.25%	0.0272
Dentomycin	4	53	54	0.064	−0.261	0.388	0.7000	0.00%	0.9730
Ligosan	3	236	232	0.408	0.063	0.753	0.0204	0.00%	0.9820
Mino ointment	4	160	158	0.303	0.011	0.595	0.0420	90.41%	0.0001
Meta-regression									
		95% CI							
Moderator		*β* coefficient		Lower		Upper		*p*-value	
Split/parallel-mouth		0.339		0.083		0.596		0.0094	
Full/partial-mouth		0		−0.338		0.338		1.0000	
Initial/maintenance		−0.106		−0.438		0.225		0.5300	
Smoking/non-smoking		−0.024		−0.438		0.39		0.9100	
(b) Long-term studies (12 + months)									
	Number of			Weighted mean difference (WMD)				Heterogeneity	
		Patients			95% CI				
12 + months	Studies	Control	Test	WMD	Lower	Upper	*p*-value	I2 (%)	*p*-value
All	18	480	485	0.310	0.24	0.381	0.0001	25.10%	0.1590
Parallel	17	461	466	0.304	0.234	0.375	0.0001	12.67%	0.3050
Split	1	19	19	1.230	0.365	2.095	0.0053	NA	NA
FM	3	151	148	0.230	0.041	0.419	0.0169	0.00%	0.6460
PM	15	329	337	0.325	0.243	0.407	0.0001	33.38%	0.1010
Initial	12	271	289	0.340	0.263	0.417	0.0001	0.00%	0.8830
Maintenance	6	209	196	0.206	−0.211	0.623	0.3330	62.59%	0.0202
SM	4	55	70	0.464	0.142	0.786	0.0048	14.22%	0.3210
NS	2	35	35	0.715	−0.244	1.675	0.1440	64.50%	0.0933
Type of tetracycline									
Tetracycline	3	58	61	0.605	0.124	1.085	0.0136	32.94%	0.2250
Doxycycline	7	202	207	0.282	0.118	0.446	0.0008	0.00%	0.4750
Minocycline	8	220	217	0.307	0.227	0.386	0.0001	42.88%	0.0925
Test product									
Actisite	2	42	45	0.795	0.093	1.497	0.0265	46.13%	0.1730
Atridox	6	183	192	0.281	0.115	0.446	0.0009	9.76%	0.3530
Dentomycin	3	30	30	0.167	−0.813	1.146	0.7390	0.00%	0.9880
Mino ointment	4	163	163	0.329	0.249	0.41	0.0001	0.00%	0.7470
Meta-regression									
				95% CI					
Moderator		*β* coefficient		Lower		Upper		*p*-value	
Split/parallel-mouth		0.926		0.058		1.793		0.0364	
Full/partial-mouth		0.093		−0.11		0.297		0.3690	
Initial/maintenance		−0.184		−0.378		0.0090		0.0617	
Smoking/non-smoking		−0.219		−0.967		0.529		0.5660	

FM, full-mouth assessment; PM, partial-mouth assessment; SM, smokers; NS, non-smokers.

For long-term studies (12 + months), 18 comparisons with 480 control and 485 test patients showed a significant CAL gain [WMD = 0.310, 95% CI (0.240; 0.381), *P* = 0.0001], with moderate heterogeneity (*I*² = 25.10%, *P* = 0.1590) (Table 4; Supplementary Figure 2B). Statistically significant, higher effects were noted for split-mouth design [*n* = 1, WMD = 1.230, 95% CI (0.365; 2.095), *P* = 0.0053], partial-mouth assessments [*n* = 15, WMD = 0.325, 95% CI (0.243; 0.407), *P* = 0.0001], and initial phase [*n* = 12, WMD = 0.340, 95% CI (0.263; 0.417), *P* = 0.0001]. Although non-smokers exhibited a greater CAL gain [*n* = 2, WMD = 0.715 mm, 95% CI (–0.244; 1.675), *p* = 0.1440], this increase did not reach statistical significance. In contrast, smokers showed a smaller but statistically significant CAL gain (*n* = 4; WMD = 0.464 mm; 95% CI, 0.142–0.786 mm; *p* = 0.0048) (Table 4).

##### 3.3.1.3 Bleeding on probing (BOP)

In medium-term studies (6–9 months), five comparisons with 127 patients per group showed a significant BOP change of 0.261 [95% CI (0.085; 0.437), *P* = 0.0036], with moderate heterogeneity (*I*² = 34.36%, *P* = 0.1920) (Table 5; Supplementary Figure 3A). No significant moderator effects were reported for study design, population, assessment type, or smoking status due to limited data (Table 5).

**Table 5:** Meta-analyses and meta-regression for bleeding on probing (BOP) changes.

(a) Medium-term Studies (6–9 months)									
	Number of			Weighted mean difference (WMD)				Heterogeneity	
		Patients			95% CI				
6–9 months	Studies	Control	Test	WMD	Lower	Upper	*p*-value	I2 (%)	*p*-value
All	5	127	127	0.261	0.085	0.437	0.0036	34.36%	0.1920
Test product									
Dentomycin	2	20	20	0.575	−0.142	1.293	0.1160	0.00%	0.8590
Arestin	1	18	18	0.06	−0.205	0.325	0.6570	NA	NA
Mino ointment	2	89	89	0.306	0.111	0.502	0.0021	59.03%	0.1180
(b) Long-term Studies (12 + months)									
	Number of			Weighted mean difference (WMD)				Heterogeneity	
		Patients			95% CI				
12 + months	Studies	Control	Test	WMD	Lower	Upper	*p*-value	I2 (%)	*p*-value
All	5	121	121	0.302	0.18	0.425	0.0001	0.00%	0.9960
Test product									
Dentomycin	3	30	30	0.353	−0.226	0.933	0.2320	0.00%	0.9210
Mino ointment	2	91	91	0.3	0.175	0.425	0.0001	0.00%	1.0000

For long-term studies (12 + months), five comparisons with 121 patients per group demonstrated a significant BOP change of 0.302 [95% CI (0.180; 0.425), *P* = 0.0001], with no heterogeneity (*I*² = 0.00%, *P* = 0.9960) (Table 5; Supplementary Figure 3B). No significant moderator effects were reported due to limited data (Table 5).

##### 3.3.1.4 Plaque index (PI)

In medium-term studies (6–9 months), 13 comparisons with 440 control and 445 test patients revealed a significant PI change of 0.089 [95% CI (0.034; 0.144), *P* = 0.0016], with no heterogeneity (*I*² = 0.00%, *P* = 0.7490) (Table 6; Supplementary Figure 4A). Significant effects were observed for parallel designs [*n* = 7, WMD = 0.102, 95% CI (0.040; 0.164), *P* = 0.0013] and initial phase [*n* = 10, WMD = 0.108, 95% CI (0.050; 0.167), *P* = 0.0003] (Table 6).

**Table 6:** Meta-analyses and meta-regression for plaque Index (PI) changes.

(a) Medium-term Studies (6–9 months)									
	Number of			Weighted mean difference (WMD)				Heterogeneity	
		Patients			95% CI				
6–9 months	Studies	Control	Test	WMD	Lower	Upper	*p*-value	I2 (%)	*p*-value
All	13	440	445	0.089	0.034	0.144	0.0016	0.00%	0.7490
Parallel	7	141	146	0.102	0.04	0.164	0.0013	0.00%	0.9540
Split	6	299	299	0.04	−0.093	0.172	0.5560	17.27%	0.3020
Initial	10	194	199	0.108	0.05	0.167	0.0003	0.00%	0.9110
Maintenance	1	30	30	−0.14	−0.478	0.198	0.4170	N/A	N/A
SM	1	20	20	0.02	−0.227	0.267	0.8740	N/A	N/A
NS	2	26	26	0.003	−0.22	0.226	0.9800	0.00%	0.8960
Type of tetracycline									
Tetracycline	1	30	30	−0.140	−0.478	0.198	0.4170	NA	NA
Doxycycline	2	216	216	−0.055	−0.252	0.143	0.5890	0.00%	0.6220
Minocycline	10	194	199	0.108	0.050	0.167	0.0003	0.00%	0.9110
Test product									
Arestin	5	79	79	0.098	−0.036	0.232	0.1510	0.00%	0.5190
Dentomycin	2	20	20	0.182	−0.173	0.536	0.3150	0.00%	0.6990
Ligosan	2	216	216	−0.055	−0.252	0.143	0.5890	0.00%	0.6220
Mino ointment	2	89	89	0.1	0.03	0.17	0.0052	0.00%	1.0000
Minocin	1	6	11	0.17	−0.025	0.365	0.0878	NA	NA
Actisite	1	30	30	−0.14	−0.478	0.198	0.4170	NA	NA
Meta-regression									
				95% CI					
Moderator		*β* coefficient		Lower		Upper		*p*-value	
Split/parallel-mouth		−0.062		−0.197		0.073		0.3670	
Initial/maintenance		−0.248		−0.591		0.095		0.1570	
Smoking/non-smoking		0.017		−0.316		0.350		0.9200	
(b) Long-term studies (12 + months)									
	Number of			Weighted mean difference (WMD)				Heterogeneity	
		Patients			95% CI				
12 + months	Studies	Control	Test	WMD	Lower	Upper	*p*-value	I2 (%)	*p*-value
All	7	147	147	0.124	0.042	0.207	0.0032	0.00%	0.6220
Test product									
Arestin	2	26	26	−0.04	−0.289	0.21	0.7550	0.00%	0.6820
Dentomycin	3	30	30	0.179	−0.126	0.484	0.2490	0.00%	0.7910
Mino ointment	2	91	91	0.145	0.047	0.242	0.0036	28.62%	0.2370

SM, smokers; NS, non-smokers.

For long-term studies (12 + months), seven comparisons with 147 patients per group showed a significant PI change of 0.124 [95% CI (0.042; 0.207), *P* = 0.0032], with no heterogeneity (*I*² = 0.00%, *P* = 0.6220) (Table 6; Supplementary Figure 4B). No significant moderator effects were reported due to limited data (Table 6).

##### 3.3.1.5 Gingival index (GI)

In medium-term studies (6–9 months), 12 comparisons with 420 control and 425 test patients demonstrated a significant change of GI [WMD = 0.155, 95% CI (0.064; 0.247), *P* = 0.0009], with moderate heterogeneity (*I*² = 63.78%, *P* = 0.0014) (Table 7; Supplementary Figure 5A). Significant yet modest improvements were noted for split-mouth design [*n* = 5, WMD = 0.202, 95% CI (0.020; 0.383), *P* = 0.0295] as well as parallel-mouth design [*n* = 7, WMD = 0.130, 95% CI (0.011; 0.249), *P* = 0.0329], initial phase [*n* = 9, WMD = 0.160, 95% CI (0.054; 0.266), *P* = 0.0031], and partial-mouth assessment [*n* = 12, WMD = 0.155, 95% CI (0.064; 0.247), *P* = 0.0009]. Lack of data hindered the comparison between the effect of full-mouth and partial-mouth assessment (Table 7).

**Table 7:** Meta-analyses and meta-regression for gingival index (GI) changes.

(a) Medium-term Studies (6–9 months)									
	Number of			Weighted mean difference (WMD)				Heterogeneity	
		Patients			95% CI				
6–9 months	Studies	Control	Test	WMD	Lower	Upper	*p*-value	I2 (%)	*p*-value
All	12	420	425	0.155	0.064	0.247	0.0009	63.78%	0.0014
Parallel	7	141	146	0.13	0.011	0.249	0.0329	71.32%	0.0019
Split	5	279	279	0.202	0.02	0.383	0.0295	52.23%	0.0788
FM	0	–	–	–	–	–	–	–	–
PM	12	420	425	0.155	0.064	0.247	0.0009	63.78%	0.0014
Initial	9	174	179	0.16	0.054	0.266	0.0031	70.45%	0.0007
Maintenance	1	30	30	−0.23	−0.75	0.29	0.3860	NA	NA
SM	0	–	–	–	–	–	–	–	–
NS	2	26	26	0.04	−0.176	0.256	0.7180	0.00%	0.8560
Type of tetracycline									
Tetracycline	1	30	30	−0.230	−0.750	0.290	0.3860	NA	NA
Doxycycline	2	216	216	0.205	0.002	0.408	0.0479	0.00%	0.3350
Minocycline	9	174	179	0.160	0.054	0.266	0.0031	70.45%	0.0007
Test product									
Arestin	4	59	59	0.178	−0.024	0.38	0.0849	54.13%	0.0881
Dentomycin	2	20	20	0.22	−0.174	0.613	0.2740	0.00%	0.6360
Ligosan	2	216	216	0.205	0.002	0.408	0.0479	0.00%	0.3350
Minocin	1	6	11	−0.03	−0.242	0.182	0.7810	NA	NA
Actisite	1	30	30	−0.23	−0.75	0.29	0.3860	NA	NA
Mino ointment	2	89	89	0.196	0	0.392	0.0498	94.35%	0.0001
Meta-regression									
				95% CI					
Moderator		*β* coefficient		Lower		Upper		*p*-value	
Split/parallel-mouth		0.072		−0.132		0.275		0.490	
Initial/maintenance		−0.39		−0.964		0.184		0.183	
Smoking/non-smoking		−0.037		−0.319		0.245		0.798	
(b) Long-term studies (12 + months)									
	Number of			Weighted mean difference (WMD)				Heterogeneity	
		Patients			95% CI				
12 + months	Studies	Control	Test	WMD	Lower	Upper	*p*-value	I2 (%)	*p*-value
All	7	147	147	0.155	0.075	0.235	0.0001	0.00%	0.5800
Test product									
Arestin	2	26	26	0.103	−0.231	0.437	0.5450	25.49%	0.2470
Dentomycin	3	30	30	0.183	−0.137	0.502	0.2630	0.00%	0.9800
Mino ointment	2	91	91	0.158	0.061	0.255	0.0014	65.24%	0.0899

FM, full-mouth assessment; PM, partial-mouth assessment; SM, smokers; NS, non-smokers.

For long-term studies (12 + months), seven comparisons with 147 patients per group showed a significant GI change of 0.155 [95% CI (0.075; 0.235), *P* = 0.0001], with no heterogeneity (*I*² = 0.00%, *P* = 0.5800) (Table 7; Supplementary Figure 5B). No significant moderator effects were reported due to limited data (Table 7).

#### 3.3.2 Meta-analysis results by drug type

The meta-analysis demonstrated that doxycycline, minocycline, and tetracycline derivatives provided significant benefits as adjuncts to non-surgical periodontal therapy, with notable differences in efficacy across formulations and timeframes.

For Tetracycline, eight medium-term studies (6–9 months) demonstrated significant improvements in both PPD [WMD = 0.74, 95% CI (0.401–1.079), *P* = 0.0001] and CAL [WMD = 0.460, 95% CI (0.114–0.806), *P* = 0.0092]. Long-term studies (≥12 months) showed significant CAL gains [*n* = 3, WMD = 0.605, 95% CI (0.124–1.085), *P* = 0.0136] but non-significant PPD changes (*P* = 0.0538). Seven RCTs using Doxycycline exhibited significant medium-term reductions in PPD [WMD = 0.454, 95% CI (0.209–0.698), *P* = 0.0003] and CAL improvements [WMD = 0.464, 95% CI (0.191–0.738), *P* = 0.0009]. Seven long-term doxycycline studies revealed significant CAL improvement [WMD = 0.282, 95% CI (0.118–0.446), *P* = 0.0008] but no significant PPD changes. Minocycline demonstrated consistent efficacy across both timeframes, with significant short-term PPD [*n* = 27, WMD = 0.46, 95% CI (0.354–0.565), *P* = 0.0001] and CAL improvements [*n* = 18, WMD = 0.241, 95% CI (0.09–0.392), *P* = 0.0017], and sustained long-term benefits in both PPD [*n* = 10, WMD = 0.580, 95% CI (0.283–0.876), *P* = 0.0001] and CAL parameters [*n* = 8, WMD = 0.307, 95% CI (0.227–0.386), *P* = 0.0001] (Tables 3, 4).

When the effect of test product was assessed separately, for tetracycline formulations (Actisite), medium-term studies showed robust PPD reductions [*n* = 5, WMD = 0.705, 95% CI (0.503–0.907), *P* = 0.0001] and moderate CAL improvements [*n* = 5, WMD = 0.264, 95% CI (0.020–0.509), *P* = 0.0343]. Long-term studies demonstrated sustained benefits in PPD [*n* = 2, WMD = 0.554, 95% CI (0.017–1.091), *P* = 0.0434] and CAL [*n* = 2, WMD = 0.795, 95% CI (0.093–1.497), *P* = 0.0265]. None of the included studies evaluating BOP used tetracycline formulations, and long-term effects on PI and GI were not reported for this group (Tables 3–7).

For doxycycline formulations, medium-term studies (6–9 months) showed significant reductions in PPD with Atridox [*n* = 4, WMD = 0.446, 95% CI (0.029–0.862), *P* = 0.0359] and Ligosan [*n* = 3, WMD = 0.518, 95% CI (0.251–0.784), *P* = 0.0001]. CAL gains were also significant: Atridox achieved WMD = 0.495 mm (*n* = 4, 95% CI 0.061–0.928, *P* = 0.0253), and Ligosan WMD = 0.408 mm [*n* = 3, 95% CI (0.063–0.753), *P* = 0.0204]. Long-term studies (≥12 months) revealed significant CAL improvement by Atridox [*n* = 6, WMD = 0.281, 95% CI (0.115–0.446), *P* = 0.0009] but no significant PPD changes. None of the selected studies reporting BOP outcomes included doxycycline formulations, and data were limited for PI and GI. Overall, doxycycline led to a modest but statistically significant effect on PPD and CAL changes (Tables 3–7).

Minocycline-based formulations consistently demonstrated significant medium-term improvements in PPD reduction [e.g., minocycline ointment *n* = 4, WMD = 0.580, 95% CI (0.345–0.814), *P* = 0.0001] and CAL gain [*n* = 4, WMD = 0.303, 95% CI (0.011–0.595), *P* = 0.0420]. BOP was significantly affected by minocycline ointment (*n* = 2, WMD = 0.306 mm, 95% CI 0.111–0.502, *P* = 0.0021), whereas Arestin and Dentomycin did not show significant BOP changes. Plaque index showed significant improvement only with minocycline ointment (*n* = 2, WMD = 0.100 mm, 95% CI 0.030–0.170, *P* = 0.0052), but not with other formulations. Gingival index changes were not significant for any of the reported minocycline formulations. Long-term data indicated persistent benefits, particularly for minocycline ointment, with significant effect in PPD reduction [*n* = 4, WMD = 0.745, 95% CI (0.496–0.994), *P* = 0.0001], CAL gain [*n* = 4, WMD = 0.329, 95% CI (0.249–0.410), *P* = 0.0001], and changes of PI and GI. Notably, long-term studies reporting PI and GI exclusively utilized minocycline formulations (Tables 3–7).

Overall, long-term data indicate that minocycline ointment confers persistent changes in PPD, CAL, BOP, PI, and GI, whereas other minocycline formulations yield less consistent benefits (Tables 3–7). In summary, minocycline demonstrated the most consistent efficacy across outcomes and durations, while doxycycline and tetracycline provided significant but more limited benefits.

#### 3.3.3 Meta-regression on moderators

Across the pooled analyses, meta-regression indicated that study design significantly influenced effect sizes. Split-mouth designs tended to show larger treatment effects: *β* = 0.422 (*P* = 0.0001) favoring split-mouth over parallel designs for PPD, and *β* = 0.339 (*P* = 0.0094) for CAL in medium-term data (Tables 3, 4). By contrast, neither smoking status (smoker vs. non-smoker) nor treatment phase (initial therapy vs. maintenance) significantly moderated the outcomes (all *P* > 0.05). No other moderators showed a significant influence on the effect sizes (Tables 3–7).

#### 3.3.4 Assessment of publication bias

Egger's test revealed significant publication bias for the primary outcome variables, PPD and CAL, across both 6–9 months and 12 + months studies. For PPD at 6–9 months, the intercept was 0.486 (*P* = 0.001), with trim-and-fill analysis imputing 9 studies (Supplementary Table 11, Supplementary Figure 6B), yielding an adjusted effect size of 0.397 (95% CI: 0.282–0.513). At 12 + months, PPD showed an intercept of 0.419 (*P* = 0.001), with 7 studies imputed (Supplementary Table 11, Supplementary Figure 7B), resulting in an adjusted effect size of 0.587 (95% CI: 0.421–0.753). For CAL at 6–9 months, the intercept was 0.309 (*P* = 0.001), with 9 studies imputed, giving an adjusted effect size of 0.189 (95% CI: 0.056–0.321); at 12 + months, no adjustment was needed despite an intercept of 0.319 (*P* = 0.003). Among secondary outcomes, BOP, PI, and GI showed no significant bias in either time frame, with minimal or no adjustments required (Supplementary Table 11). Sensitivity analyses confirmed the robustness of these findings, indicating stable effect sizes regardless of small or outlier study exclusion (Supplementary Figure 8).

## 4 Discussion

This systematic review and meta-analysis of 43 studies, encompassing 2,525 control and 2,575 test patients, evaluated the long-term efficacy of locally delivered tetracycline-class antimicrobials (tetracycline, doxycycline, minocycline) as adjuncts to SRP in the treatment of chronic periodontitis.

Despite the considerable heterogeneity observed among studies, this meta-analysis of medium- and long-term trials, adjunctive tetracycline therapy consistently enhanced the outcomes of SRP, producing additional probing depth reduction and clinical attachment gains that proved sustainable over time. In addition, it also showed improvement in secondary clinical parameters, such as BOP, PI, and GI. Although these gains are modest, they meet established thresholds for clinical relevance and may translate into decreased risk of pocket recurrence, especially in patients presenting with deep baseline pockets or systemic risk factors (e.g., smoking). These findings highlight its potential to broadly improve periodontal health, extending benefits beyond mechanical debridement alone. Notably, although several systematic reviews and meta-analyses have focused on probing depth and attachment-level outcomes, very few have examined BOP, PI, and GI in the same quantitative synthesis. Because our literature search identified a sufficient number of trials reporting these three parameters, we analyzed BOP, PI, and GI concurrently, positioning it to assess the anti-inflammatory and anti-plaque benefits of locally delivered tetracyclines beyond mechanical debridement.

Subgroup analyses were employed to explore the potential sources of heterogeneity. The analysis revealed that study design significantly affects treatment outcomes, with split-mouth studies demonstrating larger improvements in both PPD and CAL compared to parallel-group studies. In medium-term studies (6–9 months), split-mouth designs showed a weighted mean difference (WMD) of 0.819 mm for PPD and 0.564 mm for CAL (*p* < 0.05), significantly higher than parallel designs (0.391 mm and 0.215 mm, respectively). This advantage likely stems from the reduced inter-subject variability in split-mouth designs, which enhances the detection of treatment effects by controlling for individual patient differences. These findings align with prior research, which has validated split-mouth designs for their ability to isolate treatment effects with minimal cross-over effects, as demonstrated in studies using disclosing agents and irrigation to show limited retrograde perfusion (70, 73).

Moreover, the type of periodontal assessment also influenced outcomes, particularly for PPD. Partial-mouth assessments showed a trend toward greater PPD reductions, especially in long-term studies (WMD 0.444 mm vs. 0.034 mm for full-mouth, *P* < 0.05). This suggests that partial-mouth assessments are more sensitive to localized treatment effects, a finding consistent with earlier reports (100, 101). However, for CAL, differences between full-mouth and partial-mouth assessments were less pronounced, with both showing significant improvements in long-term studies (WMD 0.230 mm for full-mouth, 0.325 mm for partial-mouth, *P* < 0.05). This indicates that full-mouth debridement may provide broader benefits by addressing microbial reservoirs, potentially enhancing overall host defense mechanisms, as noted in prior studies. The choice of assessment type thus requires careful consideration based on the specific clinical outcomes targeted.

Patients in the initial treatment phase exhibited greater benefits from adjunctive antimicrobial therapy compared to those in maintenance phases, particularly in long-term studies. For PPD, the WMD was 0.498 mm (*P* = 0.0001) in initial phases vs. 0.179 mm in maintenance (*P* = 0.1800), and for CAL, 0.340 mm (*P* = 0.0001) vs. 0.206 mm (*P* = 0.3330). Although not always statistically significant, this trend suggests that untreated sites in initial phases have greater healing potential, likely due to higher baseline disease severity. This observation is in line with clinical expectations that early intervention maximizes therapeutic impact, a concept supported by previous periodontal research (102).

### 4.1 Clinical implications in smokers vs. non-smokers

When stratified by smoking status, in the medium term, both smokers and non-smokers showed comparable gains when tetracycline acted as an adjunct to SRP, indicating that smoking does not blunt the initial adjunctive effect. However, over the long term, only non-smokers maintained significant PPD reduction, whereas smokers lost this additional benefit. Attachment level gains tended to persist in both groups in the short term. The long-term improvement of CAL in non-smokers was of higher magnitude than in smokers, but with no statistical significance, likely reflecting fewer studies or greater variability in that subgroup.

A critical finding of our research is the observation that smokers demonstrate less favorable responses to periodontal therapy compared to non-smokers. Taken together, these findings suggest that while smokers can achieve similar short-term improvements with adjunctive tetracycline, smoking may undermine the durability of pocket depth reduction. The mechanisms underlying this compromised healing response in smokers are multifactorial, involving impaired vascular function, altered immune responses, and potentially increased prevalence of resistant periodontopathogens (63).

Despite the generally compromised outcomes in smokers, our findings suggest that certain tetracycline formulations may partially counteract smoking's negative impact on periodontal healing (103). Locally applied controlled-release tetracycline fiber has demonstrated particular promise in this regard, improving clinical outcomes in smokers beyond what would be expected with mechanical debridement alone (104). Similarly, smokers treated with minocycline microspheres in addition to SRP exhibited pocket depth reductions of 1.19 mm at 9 months, compared to only 0.90 mm with SRP alone (105).

However, it must be acknowledged that the evidence for adjunctive antibiotic therapy specifically in smokers remains incomplete. A previous systematic review concluded that evidence for additional benefits of adjunctive antibiotics in smokers with chronic periodontitis is insufficient and somewhat inconclusive (106). Chambrone et al. pooled seven RCTs in heavy smokers, reporting an additional 0.81 mm PPD reduction and 0.91 mm CAL gain at sites with baseline PPD ≥5 mm, whereas systemic antimicrobials showed no significant benefit in this population (37). A smoking-specific review of local doxycycline reported a 1.10 mm CAL gain at six months from two trials (38). These findings highlight that smokers, who typically respond poorly to conventional therapy, can still achieve substantial periodontal improvements with local antimicrobials, often outperforming systemic regimens. However, the need for further well-designed studies specifically targeting this patient population should be highlighted.

### 4.2 Specific observations on formulations

The meta-analysis provides evidence that tetracycline, doxycycline, and minocycline, when used as local adjuncts in the treatment of chronic periodontitis, significantly enhance key clinical outcomes, notably PPD and CAL, though efficacy varies by drug and formulation. These findings align with the established antimicrobial and host-modulatory properties of tetracycline-class antibiotics, which are known to combat periodontal pathogens and reduce inflammation. Tetracycline fibers provided robust and sustained benefits in PPD and CAL. Doxycycline products (Atridox, Ligosan) showed notable medium-term improvements but were less consistent over the long term. Minocycline's broader efficacy may be attributed to its higher lipid solubility and enhanced tissue penetration, potentially allowing greater interaction with periodontal tissues. Additionally, minocycline's ability to inhibit matrix metalloproteinases, enzymes involved in tissue destruction in periodontitis, may contribute to its superior performance across multiple clinical parameters. Moreover, the absence of data on outcomes such as BOP for doxycycline and tetracycline formulations, and PI and GI for tetracycline fibers, underscores the need for further research to fully evaluate these adjuncts' comprehensive impact.

However, the presence of moderate to high heterogeneity in some analyses (e.g., *I*² = 85.13% for minocycline in medium-term CAL, *I*² = 73.39% for minocycline in medium-term PPD) indicates variability in study designs, patient populations, or treatment protocols, which necessitates cautious interpretation. For instance, differences in drug delivery methods, dosages, or patient characteristics could influence outcomes. The lower heterogeneity in some doxycycline analyses (e.g., *I*² = 0.00% for long-term CAL) suggests more consistent results, possibly due to standardized formulations like Atridox or Ligosan. The number of studies and participants also varied significantly, with minocycline supported by a larger evidence base (e.g., 27 studies for medium-term PPD with 1,386 controls and 1,426 test participants) compared to tetracycline (8 studies for medium-term PPD) and doxycycline (7 studies for medium-term PPD). This disparity may contribute to the robustness of minocycline's findings but also highlights the need for more extensive research on tetracycline and doxycycline, particularly for outcomes like BOP, PI, and GI, where data were limited or absent.

A recent randomized trial directly comparing tetracycline collagen fibers to 2% minocycline HCl gel showed both to produce equivalent PPD reductions and CAL gains by 3 months (107). This finding is consistent with previous analyses suggesting that the key factor is the presence of local antibiotics rather than the specific formulation. In practice, tetracycline fibers (e.g., Actisite), resorbable collagen fibers, 2% tetracycline ointment, 2% minocycline microspheres, and 10% doxycycline gel all achieve similarly modest adjunctive benefits. No consistent head-to-head study has demonstrated a clinically significant difference among these agents. Thus, while pharmacokinetics (release profile, substantivity) differ by product, our data and the literature suggest that they deliver comparable adjunctive efficacy (39). Formulation choice may therefore be guided by handling characteristics or patient preference rather than expected therapeutic advantage.

Prior systematic reviews have also compared various tetracycline formulations, but among other antimicrobial agents (e.g., chlorohexidine and metronidazole). Herrera et al. provided detailed effects on PPD reduction: Actisite fibers (0.729 mm; *n* = 7), Atridox doxycycline (0.800 mm; *n* = 2), and Arestin minocycline microspheres (0.279 mm; *n* = 6) (35). While a comparatively higher number of RCTs were analyzed for Atridox (*n* = 4) and Arestin (*n* = 17) in our study, we identified only five eligible Actisite trials. Methodological differences in study selection criteria likely explain the discrepancy (e.g., inclusion of systemic antibiotics or duplicate publications). Nadig and Shah included one doxycycline, six minocycline, and three tetracycline-fiber trials but did not report agent-specific WMDs, limiting head-to-head comparison (36). Matesanz-Pérez et al. noted that tetracycline fibers (0.850 mm; *n* = 1) produced greater pocket reduction than sustained-release doxycycline (0.562 mm; *n* = 1) or minocycline (0.500 mm; *n* = 1) in medium-term follow-up, though only one study has been analyzed (34). Our review, which incorporated more trials for each agent and extended follow-ups, yielded a PPD reduction of 0.740 mm in 6–9 months (*n* = 8) and 0.427 mm in 12 + months (*n* = 3) for tetracycline fibers. Our doxycycline PPD outcomes (6–9 months; *n* = 7, WMD = 0.454 mm, 12 + months; *n* = 7, WMD = 0.143 mm) were similar to the 0.562 mm (*n* = 1) and 0.100 mm (*n* = 1) reported by Matesanz-Pérez. These comparisons underscore that all tetracycline formulations improve clinical outcomes, but fiber- or gel-based vehicles may confer marginally greater pocket closures when examined over extended periods.

Nonetheless, clinicians should consider delivery system characteristics, as these may influence long-term outcomes, though the overall adjunctive effect remains modest. Our results highlight that the choice of local antibiotic should consider the specific delivery system's longevity and release profile. Clinicians might expect somewhat greater long-term pocket closure with a prolonged-release minocycline ointment or doxycycline systems, while acknowledging that any adjunctive drug effect is modest.

### 4.3 Comparison with previous studies

Systematic reviews in this field vary widely in scope, affecting the robustness and comparability of their findings. Our meta-analysis included 42 randomized controlled trials (RCTs), emphasizing longer follow-ups (6–9 and ≥12 months) and a broad selection of tetracycline fibers, minocycline, and doxycycline products. By comparison, Nadig and Shah focused solely on tetracycline-based local delivery, but only considered 10 RCTs, in contrast to 52 studies analyzed in ours, which may have led to overestimated effect sizes due to the limited evidence base (36). Herrera et al. reviewed 50 investigations reported in 59 papers, encompassing various local antimicrobials (35), while Matesanz-Pérez et al. analyzed 52 studies across 56 papers with a similar focus (34). Although these larger reviews offer comprehensive overviews, they also introduce greater heterogeneity in study designs, follow-up durations, and formulations.

Hanes and Purvis (108); Bonito et al. (33) reported mean pocket reductions on the order of 0.3–0.6 mm with local antibiotics. In contrast, Nadig and Shah's meta-analysis of 10 trials (588 sites) found ∼1.2 mm PPD reduction and ∼1.0 mm CAL gain with adjunctive tetracycline vs. control (36), likely driven by their use of standardized mean differences and inclusion of unpublished data. Our WMD for PPD reduction (0.516 mm) and CAL gain (0.336 mm) in 6–9 months and 0.371 mm and 0.310 mm in 12 + months respectively, more closely align with broader reviews. For instance, Herrera et al. reported a WMD of 0.365 mm for PPD reduction at 6–9 months and 0.190 mm at 12–60 months, with CAL gains of 0.263 mm and 0.090 mm, respectively (35). Matesanz-Pérez et al. found a WMD of 0.407 mm for PPD reduction and 0.310 mm for CAL gain (34), closely matching Herrera et al. for shorter-term outcomes. These variations in WMD likely reflect differences in follow-up duration, statistical methods, and specific antimicrobial agents evaluated.

To address comparative efficacy between systemic and local antimicrobials, Teughels et al. reported a WMD of 0.485 mm for PPD reduction at ≥6 months with systemic antimicrobials (109), roughly similar to the 0.365–0.407 mm reductions documented for local antimicrobials by Herrera et al. and Matesanz-Pérez et al. (34, 35). However, systemic regimens carry higher risks of adverse events and antibiotic resistance, whereas local delivery targets specific periodontal sites and avoids systemic side effects, reducing the emergence of resistant flora. Though Herrera et al. (13) found that adjunctive systemic antibiotics (e.g., amoxicillin/metronidazole) improve PPD and CAL vs. SRP alone, estimates vary by drug and patient population. Our effect sizes for local tetracycline (∼0.5 mm PPD reduction and ∼0.3 mm CAL gain) are comparable to systemic regimens but without systemic side effects. Prior meta-analyses consistently show that tetracycline-class local adjuncts provide moderate yet significant PPD and CAL improvements, and our long-term results fall squarely within those ranges. The greater magnitude of benefit seen with local delivery in smokers further supports favoring local over systemic antimicrobials in this population.

### 4.4 Clinical implications

The significant improvements in PPD, CAL, BOP, PI, and GI suggest that local tetracycline-class antimicrobials are valuable adjuncts to SRP, particularly for patients with persistent or recurrent periodontal pockets. The similar medium-term benefits in smokers and non-smokers indicate that these therapies can be broadly applied, enhancing outcomes across diverse patient populations. However, the potential for reduced long-term PPD benefits in smokers highlights the need for tailored treatment plans. Clinicians should consider more frequent monitoring and maintenance visits for smokers, alongside smoking cessation programs to optimize long-term outcomes.

The choice of antimicrobial product may also influence results. Tetracycline fibers (Actisite) and minocycline ointment demonstrated robust and sustained effects, suggesting they may be preferred for patients requiring long-term management. Doxycycline products, while effective in the medium term, showed less consistent long-term PPD benefits, which clinicians should weigh when selecting treatments. The minimal adverse effects reported enhance the appeal of local antimicrobials, as they offer a safe adjunctive option compared to systemic therapies. However, practical considerations, such as the time required for application and patient adherence to post-treatment instructions (e.g., avoiding certain foods), should be addressed to ensure efficacy.

For smoking patients specifically, locally delivered tetracyclines may partially overcome the diminished response to conventional therapy typically observed in this population. While not completely negating smoking's detrimental effects, these agents can help narrow the therapeutic gap between smokers and non-smokers.

### 4.5 Cost effectiveness

Local antimicrobial delivery systems can reduce the need for surgical interventions while maintaining clinical outcomes and potentially offering cost savings and shorter treatment times compared with conventional mechanical therapy (110). Heasman et al. highlighted that systemic antimicrobials can also be cost-effective (111); however, these analyses often overlook the potential impact of increased bacterial resistance on patient management.

While locally delivered tetracycline-class antimicrobials are effective, their use must be considered in the context of both clinical and economic factors. Their benefits may be most pronounced in high-risk groups, such as smokers, patients with recurrent disease, or those with furcation lesions, where modest clinical gains could prevent more extensive and costly interventions. However, locally delivered agents generally involve higher costs relative to their short-term clinical gains, particularly when applied as sustained-release formulations. Evaluating their efficacy is further complicated by the need to consider product costs—including the agent, number of teeth treated, number of applications, wastage, clinician time, and patient visits—against the expected benefit. Cost analyses indicate that treating a single tooth at a dedicated visit represents the most expensive scenario for all local delivery systems, whereas treating multiple teeth offers substantial savings (112). Existing studies are also limited by short follow-up periods and rarely incorporate broader patient-centered outcomes, such as tooth retention, function, aesthetics, or quality of life. These limitations highlight the need for future research that integrates clinical outcomes with comprehensive economic assessments, including evaluations of effectiveness in furcation areas, to support evidence-based decision-making in periodontal therapy.

### 4.6 Limitations

Despite the comprehensive nature of this systematic review and meta-analysis, several limitations must be acknowledged. First, the included studies exhibited substantial heterogeneity, with I² values as high as 74.43% for PPD in long-term studies. This variability may be attributed to differences in study design (split-mouth vs. parallel), patient populations (initial vs. maintenance), assessment methods (full-mouth vs. partial-mouth), and antimicrobial formulations, which could affect the precision of effect estimates. Second, most studies were assessed as having a high risk of bias due to inadequate blinding, randomization, or reporting of methodological details. Only three studies were rated as having a moderate risk of bias (21, 76, 89), underscoring the need for higher-quality trials in this area.

Third, Egger's test indicated significant publication bias for PPD and CAL, with adjusted effect sizes slightly different than the unadjusted estimates. The exclusion of grey literature and restriction to English-language publications likely amplified publication bias by overrepresenting positive or significant findings and omitting null or negative results often found in unpublished or non-English sources. Consequently, our effect estimates may be inflated, and their generalizability to non-English–speaking populations limited.

Fourth, seventeen studies did not report the smoking status of their cohorts, and among those that did, definitions of smoking varied (e.g., current vs. former smokers, smoking intensity). This limited the number of studies available for subgroup analyses and introduced potential inconsistency in the results. Fifth, some subgroup analyses were based on a small number of studies (e.g., four studies for smokers' long-term PPD), potentially reducing statistical power to detect differences.

Moreover, our search strategy, relying only on keywords, “SRP” and “chronic periodontitis,” might have overlooked recent studies using the updated classification, possibly omitting valuable contemporary evidence. As clinical practice shifts toward the 2017 framework, previous terminology may require clinicians to reinterpret results within the current diagnostic context, possibly reducing their direct relevance to modern practice; conversely, because relatively few studies to date have employed the current terminology, the research community is only gradually adapting, underscoring the need for additional investigations using the updated categorization.

Finally, the focus on clinical parameters omitted patient-reported outcomes or quality of life measures, which are increasingly important for a holistic evaluation of treatment effectiveness. The cost-effectiveness of adjunctive local tetracycline therapy was also not evaluated in the included studies, and given that these agents can be expensive and yield only modest clinical gains, it is essential to determine whether the additional expense is justified in routine practice, especially in resource-limited settings. These limitations suggest that while the findings provide valuable insights, they should be interpreted with caution, and further research is needed to confirm and expand upon these results.

### 4.7 Conclusion and future directions

This study provides robust evidence that tetracycline, doxycycline, and minocycline, when used as local adjuncts in the treatment of chronic periodontitis, significantly enhance key clinical outcomes, notably PPD and CAL, with benefits observed in both smokers and non-smokers and with split-mouth studies showing the largest effect sizes. Treatment phase did not moderate these benefits meaningfully, underscoring the broad applicability of these agents in both initial and maintenance periodontal therapy. Minocycline ointment emerged as the most consistent and sustained adjunct across all evaluated clinical parameters. While medium-term benefits are comparable, long-term PPD reduction may be reduced in smokers, likely due to impaired healing. Clinicians should integrate these adjuncts into treatment protocols, especially for high-risk patients, and tailor strategies for smokers with enhanced monitoring and smoking cessation support.

Future research should prioritize high-quality randomized controlled trials with larger sample sizes, longer follow-up periods, and standardized smoking status definitions. Mechanistic studies investigating smoking's impact on treatment efficacy and comparative studies evaluating different local agents in smokers are needed. Cost-effectiveness analyses and exploration of combined therapies, such as with host modulation or laser therapy, could further guide clinical practice and improve outcomes for periodontitis patients.

Furthermore, while the current review provides comprehensive insights into the efficacy of tetracycline-class local antimicrobials in the treatment of chronic periodontitis, there remains a notable gap in the literature regarding their specific impact on furcation lesions. Furcation lesions, which occur in multi-rooted teeth and represent a significant challenge in periodontal therapy, have been less frequently studied in the context of adjunct local antimicrobial use. Given the unique anatomical and clinical challenges presented by furcation involvement, future research should prioritize conducting dedicated studies to evaluate the effectiveness of these adjuncts in managing Grade II/III furcation areas. Such investigations could provide valuable data on whether sustained-release tetracyclines can overcome the unique anatomical challenges of furcation areas and translate into improved long-term tooth prognosis in these high-risk sites.

## Data Availability

The original contributions presented in the study are included in the article/Supplementary Material, further inquiries can be directed to the corresponding author.
