# Supplementary material for: The long-term efficacy of tetracycline class antimicrobials as local adjuncts in the treatment of chronic periodontitis: a systematic review and meta-analysis
Source: Front Dent Med. 2025 Sep 26;6:1658720. doi: 10.3389/fdmed.2025.1658720 (PMC12511111; doi:10.3389/fdmed.2025.1658720)
Supplement: Supplementary file 1 [file Table1.docx]

**Supplementary Appendix 1-3**

**Appendix 1: Search strategy**

Search timeline: Dec 2024

*Restriction: in English, in Humans, Clinical Trials*

| **Database** | **Search Strategy** |
| --- | --- |
| **Medline/PubMed** | ("Periodontal Diseases"[Mesh] OR periodontal disease*[tiab] OR periodontitis*[tiab])  AND ("Tetracyclines"[Mesh] OR doxycycline*[tiab] OR minocycline*[tiab])  AND (local*[tiab] OR "slow release*"[tiab] OR topical*[tiab]) AND ("Randomized Controlled Trial"[pt] OR randomized controlled trial*[tiab] OR rcts*[tiab] OR clinical trial*[tiab]) |
| **Cochrane Library** | (“periodontal disease” OR “periodontitis”):ti,ab,kw  AND (“tetracycline” OR “doxycycline” OR “minocycline”):ti,ab,kw  AND (“local” OR “slow release” OR "topical*):ti,ab,kw  AND (“Randomized controlled trials” OR “RCTs” OR “clinical trials”):ti,ab,kw |
| **Embase** | 1. 'periodontal disease'/exp OR 'periodontitis'/exp OR 'periodontal disease*':ti,ab,kw OR 'periodontitis':ti,ab,kw  2. 'tetracycline'/exp OR 'doxycycline':ti,ab,kw OR 'minocycline*':ti,ab,kw  3. 'local*':ti,ab,kw OR 'slow release':ti,ab,kw OR 'topical':ti,ab,kw  4. 'randomized controlled trial'/exp OR 'randomized controlled trial*':ti,ab,kw OR 'rct*':ti,ab,kw OR 'clinical trial*':ti,ab,kw |
| **Scopus** | (TITLE-ABS-KEY ("periodontal disease*" OR periodontitis)) AND (TITLE-ABS-KEY ("tetracycline" OR doxycycline OR minocycline* OR "anti-bacterial agent*"))  AND (TITLE-ABS-KEY (local* OR "slow release" OR topical))  AND (TITLE-ABS-KEY ("randomized controlled trial*" OR RCT* OR "clinical trial*")) |

| **Appendix 2: PRISMA 2020 Checklist** | | | |
| --- | --- | --- | --- |
| **Section and Topic** | **Item #** | **Checklist item** | **Location where item is reported** |
| **TITLE** | | |  |
| Title | 1 | Identify the report as a systematic review. | Title page: “Systematic Review and Meta-analysis” |
| **ABSTRACT** | | |  |
| Abstract | 2 | See the PRISMA 2020 for Abstracts checklist. | Abstract (structured headings) on Page 2 |
| **INTRODUCTION** | | |  |
| Rationale | 3 | Describe the rationale for the review in the context of existing knowledge. | Introduction (Page 4; lines 124-143) |
| Objectives | 4 | Provide an explicit statement of the objective(s) or question(s) the review addresses. | Introduction/Methods (Page 4; lines 152-158) |
| **METHODS** | | |  |
| Eligibility criteria | 5 | Specify the inclusion and exclusion criteria for the review and how studies were grouped for the syntheses. | Methods (Page 5; lines 184-198) |
| Information sources | 6 | Specify all databases, registers, websites, organisations, reference lists and other sources searched or consulted to identify studies. Specify the date when each source was last searched or consulted. | Methods (Page 5, lines 161-173), Supplementary Appendix 1 |
| Search strategy | 7 | Present the full search strategies for all databases, registers and websites, including any filters and limits used. | Methods (Page 5, lines 161-173), Supplementary Appendix 1 |
| Selection process | 8 | Specify the methods used to decide whether a study met the inclusion criteria of the review, including how many reviewers screened each record and each report retrieved, whether they worked independently, and if applicable, details of automation tools used in the process. | Methods (Pages 5-6, lines 184-198, 210-217) |
| Data collection process | 9 | Specify the methods used to collect data from reports, including how many reviewers collected data from each report, whether they worked independently, any processes for obtaining or confirming data from study investigators, and if applicable, details of automation tools used in the process. | Methods (Page 6, lines 210-217) |
| Data items | 10a | List and define all outcomes for which data were sought. Specify whether all results that were compatible with each outcome domain in each study were sought (e.g. for all measures, time points, analyses), and if not, the methods used to decide which results to collect. | Methods (Page 6; lines 214-217) |
|  | 10b | List and define all other variables for which data were sought (e.g. participant and intervention characteristics, funding sources). Describe any assumptions made about any missing or unclear information. | Methods (Page 6; lines 214-217) |
| Study risk of bias assessment | 11 | Specify the methods used to assess risk of bias in the included studies, including details of the tool(s) used, how many reviewers assessed each study and whether they worked independently, and if applicable, details of automation tools used in the process. | Methods (Page 6; lines 218-226) |
| Effect measures | 12 | Specify for each outcome the effect measure(s) (e.g. risk ratio, mean difference) used in the synthesis or presentation of results. | Methods (Page 6; lines 228-238) |
| Synthesis methods | 13a | Describe the processes used to decide which studies were eligible for each synthesis (e.g. tabulating the study intervention characteristics and comparing against the planned groups for each synthesis (item #5)). | Results (Page 7; lines 256-275, Page 8; 310-321) |
|  | 13b | Describe any methods required to prepare the data for presentation or synthesis, such as handling of missing summary statistics, or data conversions. | Methods (Page 7; lines 228-246) |
|  | 13c | Describe any methods used to tabulate or visually display results of individual studies and syntheses. | Results (Tables 1–7; Pages 31-39) |
|  | 13d | Describe any methods used to synthesize results and provide a rationale for the choice(s). If meta-analysis was performed, describe the model(s), method(s) to identify the presence and extent of statistical heterogeneity, and software package(s) used. | Methods (Pages 6-7; lines 228-246) |
|  | 13e | Describe any methods used to explore possible causes of heterogeneity among study results (e.g. subgroup analysis, meta-regression). | Methods (Page 7; lines 242-246) |
|  | 13f | Describe any sensitivity analyses conducted to assess robustness of the synthesized results. | Methods (Page 7; lines 248-254) |
| Reporting bias assessment | 14 | Describe any methods used to assess risk of bias due to missing results in a synthesis (arising from reporting biases). | Methods (Page 7; lines 248-254) |
| Certainty assessment | 15 | Describe any methods used to assess certainty (or confidence) in the body of evidence for an outcome. | Methods (Page 6; 218-226, Page 7; lines 240-254) |
| **RESULTS** | | |  |
| Study selection | 16a | Describe the results of the search and selection process, from the number of records identified in the search to the number of studies included in the review, ideally using a flow diagram. | Results (Page 7; lines 256-276); Figure 1 |
|  | 16b | Cite studies that might appear to meet the inclusion criteria, but which were excluded, and explain why they were excluded. | Results (Page 7; lines 256-276); Figure 1 |
| Study characteristics | 17 | Cite each included study and present its characteristics. | Results (Pages 8-10, Tables 1–2; Pages 31-33), Supplementary tables 1-8 |
| Risk of bias in studies | 18 | Present assessments of risk of bias for each included study. | Results (Pages 10-11; lines 389-424) and Supplementary Table 9 |
| Results of individual studies | 19 | For all outcomes, present, for each study: (a) summary statistics for each group (where appropriate) and (b) an effect estimate and its precision (e.g. confidence/credible interval), ideally using structured tables or plots. | Results (Pages 11-14; lines 435-565), Tables 3-7 |
| Results of syntheses | 20a | For each synthesis, briefly summarise the characteristics and risk of bias among contributing studies. | Results (pp. 25–28, first paragraphs) |
|  | 20b | Present results of all statistical syntheses conducted. If meta-analysis was done, present for each the summary estimate and its precision (e.g. confidence/credible interval) and measures of statistical heterogeneity. If comparing groups, describe the direction of the effect. | Results (Pages 34-39, Tables 3-7) |
|  | 20c | Present results of all investigations of possible causes of heterogeneity among study results. | Results (Pages 34-39, Tables 3-7) |
|  | 20d | Present results of all sensitivity analyses conducted to assess the robustness of the synthesized results. | Results (Page 14; lines 576-577), Supplementary figures: 8A, 8B |
| Reporting biases | 21 | Present assessments of risk of bias due to missing results (arising from reporting biases) for each synthesis assessed. | Results (Page 14; lines 566-574) Supplementary figures 6-7 |
| Certainty of evidence | 22 | Present assessments of certainty (or confidence) in the body of evidence for each outcome assessed. | Results (Page 10; lines 389-424, Page 14; lines 566-574) |
| **DISCUSSION** | | |  |
| Discussion | 23a | Provide a general interpretation of the results in the context of other evidence. | Discussion (Page 15; lines 579-621) |
|  | 23b | Discuss any limitations of the evidence included in the review. | Discussion (Pages 19-20; lines 788-821) |
|  | 23c | Discuss any limitations of the review processes used. | Discussion (Pages 19-20; lines 788-821) |
|  | 23d | Discuss implications of the results for practice, policy, and future research. | Discussion (Pages 18-19; lines 748-767, Page 20; lines 823-848) |
| **OTHER INFORMATION** | | |  |
| Registration and protocol | 24a | Provide registration information for the review, including register name and registration number, or state that the review was not registered. | Methods (Page 5; lines 170-173) |
|  | 24b | Indicate where the review protocol can be accessed, or state that a protocol was not prepared. | Methods (Page 5; line 173) |
|  | 24c | Describe and explain any amendments to information provided at registration or in the protocol. | No amendments have been made to the protocol. |
| Support | 25 | Describe sources of financial or non-financial support for the review, and the role of the funders or sponsors in the review. | Funding statement; Declaration |
| Competing interests | 26 | Declare any competing interests of review authors. | Conflict of interest (page 4) |
| Availability of data, code and other materials | 27 | Report which of the following are publicly available and where they can be found: template data collection forms; data extracted from included studies; data used for all analyses; analytic code; any other materials used in the review. | Data availability (page 6) |

**Appendix 3. Papers excluded after full-paper assessment, and main reasons for exclusion in the selection of studies**

| Reference | Main reasons for exclusion |
| --- | --- |
| (Cortelli et al., 2008) | Only microbiological data, no clinical data reported. |
| (Michalowicz et al., 1995) | Only microbiological data, no clinical data reported. |
| (Ratka-Krüger et al., 2005) | Only microbiological data, no clinical data reported. |
| (Socransky et al., 2013) | Only microbiological data, no clinical data reported. |
| (Wilson et al., 1997) | Only microbiological data, no clinical data reported. |
| (Wong et al., 1998) | Only microbiological data, no clinical data reported. |
| (Tomasi & Wennstrom, 2011) | Overlapping Data |
| (Colombo et al., 2003) | Only microbiological data, no clinical data reported. |
| (Rodrigues et al., 2004) | Only microbiological data, no clinical data reported. |

**References**:

1. Cortelli JR, Aquino DR, Cortelli SC, Carvalho-Filho J, Roman-Torres CVG, Costa FO. A double-blind randomized clinical trial of subgingival minocycline for chronic periodontitis. *J Oral Sci* (2008) 50:259–265. doi: 10.2334/josnusd.50.259
2. Michalowicz BS, Pihlstrom BL, Drisko CL, Cobb CM, Killoy WJ, Caton JG, Lowenguth RA, Quinones C, Encarnacion M, Knowles M. Evaluation of periodontal treatments using controlled-release tetracycline fibers: maintenance response. *J Periodontol* (1995) 66:708–715. doi: 10.1902/jop.1995.66.8.708
3. Ratka-Krüger P, Schacher B, Bürklin T, Böddinghaus B, Holle R, Renggli HH, Eickholz P, Kim T-S. Non-surgical periodontal therapy with adjunctive topical doxycycline: a double-masked, randomized, controlled multicenter study. II. Microbiological results. *J Periodontol* (2005) 76:66–74. doi: 10.1902/jop.2005.76.1.66
4. Socransky SS, Haffajee AD, Teles R, Wennstrom JL, Lindhe J, Bogren A, Hasturk H, van Dyke T, Wang X, Goodson JM. Effect of periodontal therapy on the subgingival microbiota over a 2-year monitoring period. I. Overall effect and kinetics of change. *J Clin Periodontol* (2013) 40:771–780. doi: 10.1111/jcpe.12117
5. Wilson TG, McGuire MK, Greenstein G, Nunn M. Tetracycline fibers plus scaling and root planing versus scaling and root planing alone: similar results after 5 years. *J Periodontol* (1997) 68:1029–1032. doi: 10.1902/jop.1997.68.11.1029
6. Wong MY, Lu CL, Liu CM, Hou LT, Chang WK. Clinical response of localized recurrent periodontitis treated with scaling, root planing, and tetracycline fiber. *J Formos Med Assoc* (1998) 97:490–497
7. Tomasi C, Wennström JL. Locally delivered doxycycline as an adjunct to mechanical debridement at retreatment of periodontal pockets: outcome at furcation sites. *J Periodontol* (2011) 82:210–218. doi: 10.1902/jop.2010.100308
8. Colombo A, Gonçalves C, Rodrigues RMJ, Souto R, Uzeda M, Feres-Filho E. Microbiological evaluation of adjunctive systemic and local tetracycline administration combined with scaling and root planing in the treatment of chronic periodontitis. *Brazilian Journal of Oral Sciences* (2003) https://www.semanticscholar.org/paper/Microbiological-evaluation-of-adjunctive-systemic-Colombo-Gon%C3%A7alves/ac8180d5219f22501f7a37a8c763facb77ef772a [Accessed April 22, 2025]
9. Rodrigues RMJ, Gonçalves C, Souto R, Feres-Filho EJ, Uzeda M, Colombo APV. Antibiotic resistance profile of the subgingival microbiota following systemic or local tetracycline therapy. *J Clin Periodontol* (2004) 31:420–427. doi: 10.1111/j.1600-051X.2004.00493.x
