# Supplementary material for: The long-term efficacy of tetracycline class antimicrobials as local adjuncts in the treatment of chronic periodontitis: a systematic review and meta-analysis
Source: Front Dent Med. 2025 Sep 26;6:1658720. doi: 10.3389/fdmed.2025.1658720 (PMC12511111; doi:10.3389/fdmed.2025.1658720)
Supplement: Supplementary file 2 [file Datasheet1.docx]

# Supplementary Tables

Supplementary Table 1. Study settings and patient recruitment.

| **Study reference** | **Setting** | **Support** | **Other control** | **Other test** | **Initial/maintenance** |
| --- | --- | --- | --- | --- | --- |
| Aboelsaad et al., 2014 | University | No | - | - | Initial |
| Agan et al., 2006 | University | NR | - | - | Initial |
| Ahamed et al., 2013 | University | No | - | - | Initial |
| Aimetti et al., 2004 | University | Dental Triy | - | - | Maintenance |
| Bogren et al., 2008 | Private practice, University | Public funds | - | - | Maintenance |
| Cortelli et al., 2006; Cortelli et al., 2008 | University | Argentine Society of Periodontology | - | - | Initial |
| Dannewitz et al., 2009 | University | NR | - | - | Maintenance |
| Deo et al., 2011 | University | NR | - | - | Initial |
| Drisko et al., 1995; Michalowics et al., 1995 | University | Palo Alto | - | Actisite alone Actisite 2x | Initial & Maintenance |
| Eickholz et al., 2002; Ratka-Krüger et al., 2005 | University | Ivoclar Vivadent | Vehicle control | - | Initial & Maintenance |
| Flemmig et al., 1996 | University | Alza, Palo Alto | - | - | Maintenance |
| Friesen et al., 2002 | University | Alza, Palo Alto; P&G | Un RX | Multiple TT | Initial |
| Gonçalves et al., 2004; Colombo et al., 2003; Rodrigues et al., 2004 | University | Multiple funders such as PRONEX | - | Systemic T | Initial |
| Goodson et al., 1985 | University | Public funds | Un RX | Actisite alone | Initial |
| Goodson et al.,2012; Socransky et al., 2013 | University | Public funds | - | Six other groups | Initial |
| Gopinath et al., 2009 | University | NR | - | - | Initial |
| Henderson et al., 2002 | University | Ora Pharma | - | - | Initial |
| Jain et al., 2012 | University | No | - | - | Initial |
| Jones et al., 1994 | University | Lederle | Un Rx | MINO alone | Initial |
| Killeen et al., 2016 | University | Reinhard Foundation | - | - | Maintenance |
| Kinane & Radvar, 1999 | University | NR | - | MET alone | Maintenance |
| Lie et al., 1998 | University | Colgate | - | MET alone | Initial |
| Machion et al., 2004 | University | FAPESP | - | - | Initial |
| Machion et al., 2006 | University | CAPES | -- | - | Maintenance |
| Meinberg et al., 2002 | University | Ora Pharma | - | - | Maintenance |
| Newman et al., 1994; Wilson et al., 1997 | Private practice | P & G | - | - | Maintenance |
| OPI study (103A) 2000 | NR | Ora Pharma | - | - | Initial |
| OPI study (103B) 2000 | NR | Ora Pharma | - | - | Initial |
| Oringer et al., 2002 | University | NR | - | Mino + SRP Open label | Initial |
| Reddy et al., 2016 | University | NR | - | CHX | Maintenance |
| Singh et al., 2014 | University | No | - | CHX | Initial |
| Soeroso et al., 2017 | University | Sunstar & public funds | - | - | Initial |
| Sweatha et al., 2015 | University | NR | - | - | Initial |
| Tabenski et al., 2017 | University | Helbo Photodynamic sytem | - | Photodynamic therapy | Initial |
| Timmerman et al., 1996 | University | Cyanamid | - | - | Initial |
| Tomasi et al., 2008; Tomasi & Wennstrom, 2011 | University | Praktikert-janst Stockholm | - | - | Initial |
| Tonetti et al., 1998 | Private practice | Alza | - | - | Maintenance |
| Tonetti et al., 2012 | Private practice | Ivoclar vivadent | - | - | Maintenance |
| Van Dyke et al., 2002 | University | NR | Un Rx. | Arestin alone | Initial |
| Van Steenberghe et al., 1999 | University | NR | - | - | Initial |
| Williams et al., 2001 | University | Oral Pharma | - | - | Initial |
| Wong et al., 1998; Wong et al., 1999 | University | Public funds | - | - | Maintenance |
| Zingale et al., 2012 | University | OraPharma | Papilla reflection + SRP | Papilla reflection + SRP + Arestin | Maintenance |

CHX, chlorhexidine; MET, metronidazole, MINO, minocycline; NR, not reported; TET, tetracycline; Un Rx, untreated

Supplementary Table 2 - Study Characteristics

|  |  |  | **Test group** | | | | | **Control group** | | | | |
| --- | --- | --- | --- | --- | --- | --- | --- | --- | --- | --- | --- | --- |
| **Study reference** | **Blinding** | **Design** | **Treatment** | **Sample size baseline/end** | **Age*** | **% Female** | **% Smokers** | **Treatment** | **Sample size baseline/end** | **Age*** | **% Female** | **% Smokers** |
| Aboelsaad et al., 2014 | Single | Split | Arestin | 20/NR | 37 (31-49) | 75 | 100 | SRP alone | 20/NR | 37 (31-49) | 75 | 100 |
| Agan et al., 2006 | Single | Split | Atridox | 10/NR | 55(41-69) | 40 | NR | SRP alone | 10/NR | 55 (41-69) | 40 | NR |
| Ahamed et al., 2013 | NR | Parallel | Atridox | 6/6 | NR | NR | NR | SRP alone | .6/6 | NR | NR | NR |
| Aimetti et al., 2004 | Single | Split | Actisite | 19/19 | 47±10.78 (NR) | 58 | Non-smokers | SRP alone | 19/19 | 47±10.78 (NR) | 58 | Non-smokers |
| Bogren et al., 2008 | Single | Parallel | Atridox | 63/60 | 58 (34-77) | 59 | 28 | SRP alone | 65/64 | 60 (35-82) | 58 | 31 |
| Cortelli et al., 2006; Cortelli et al., 2008 | Double | Parallel | Arestin | 13 | NR | NR | Non-smokers | SRP+vehicle | 13 | NR | NR | Non-smokers |
| Dannewitz et al., 2009 | Single | Parallel | Ligosan  (14%_doxycycline) | 19/15 | 52.6±9.7 (NR) | 47.4 | 26.3 | SRP alone | 20/19 | 50.3±8.2 (NR) | 70 | 25 |
| Deo et al., 2011 | Single | Parallel | Atridox | 30/30 | NR | NR | Non-smokers | SRP+placebo | 30/30 | NR | NR | Non-smokers |
| Drisko et al., 1995 | Single | Split | Actisite | 122/116 | 45.1 (25-73) | 44.3 | NR | SRP alone | 122/116 | 45.1 (25-73) | 44.3 | NR |
| Eickholz et al., 2002; Ratka-Krüger et al., 2005 | Double | Split | Ligosan | 111/110 | 49.9±9.8 (23-71) | 63 | 37 | SRP+vehicle | 111/110 | 49.9±9.8 (23-71) | 63 | 37 |
| Flemmig et al., 1996 | Single | Split | Actisite | 35/28 | 53.6±12.4 (NR) | 35.7 | NR | SRP alone | 35/28 | 53.6±12.4 (NR) | 35.7 | NR |
| Friesen et al., 2002 | Single | Split | Tetra strips (single) | 28/24 | 43.6 (26-69) | 53.6 | NR | SRP alone | 28/24 | 43.6 (26-69) | 53.6 | NR |
|  |  |  | Tetra strips (multiple) | 28/24 | 43.6 (26-69) | 53.6 | NR |  |  |  |  |  |
| Gonçalves et al., 2004; Colombo et al., 2003; Rodrigues et al., 2004 | Single | Parallel | Actisite | 10/NR | 45±9 | 70 | 20 | SRP alone | 10/NR | 46±8 (NR) | 60 | 20 |
| Goodson et al., 1985 | Single | Split | Actisite | 10/NR | 38 (27-52) | 70 | NR | SRP alone | 10/NR | 38 (27-52) | 70 | NR |
| Goodson et al.,2012; Socransky et al., 2013 | Single | Parallel | Actisite | 30/26 | 46±2.0 (NR) | NR | 27 | SRP alone | 28/23 | 47±2.6 (NR) | NR | 23.3 or 30% |
| Gopinath et al.,2009 | NR | Split | Arestin | 15/NR | NR (35-50) | NR | NR | SRP alone | 15/NR | NR (35-50) | NR | NR |
| Henderson et al., 2002 | Single | Split | Arestin | 15/15 | 46.3 (35-69) | 53.3 | 40 | SRP alone | 15/15 | 46.3 (35-69) | 53.3 | 40 |
| Jain et al., 2012 | NR | Split | Dentomycin 2% min gel | 15/13 | NR | NR | Non-smokers | SRP alone | 15/13 | NR | NR | Non-smokers |
| Jones et al., | Double | Parallel | Minocin (Mino. Powder) | NR/11 | NR | NR | NR | SRP alone | NR /6 | NR | NR | NR |
| Killeen et al., 2016 | Single | Parallel | Arestin | 30/24 | 66.8±10.0 (NR) | 20.8 | 33.3 | SRP alone | 30/27 | 67.0±12.2 | 40.7 | 14.8 |
| Kinane & Radvar, 1999 | Single | Parallel | Dentomycin | 22/21 | NR (44.3-46.3) | NR | NR | SRP alone | 21/20 | NR (44.3-46.3) | NR | NR |
|  |  |  | Actisite | 19/19 | NR (44.3-46.3) | NR | NR | SRP alone | 21/20 | NR (44.3-46.3) | NR | NR |
| Lie et al., 1998 | Single | Split | Aureomycin (Tet. ointment) | 18/18 | NR (36-77) | NR | NR | SRP alone | 18/18 | NR (36-77) | NR | NR |
| Machion et al., 2004 | Single | Parallel | Atridox | 22/22 | 40.45±4.47 | NR | Smokers | SRP alone | 21/21 | 42.00±4.38 | NR | Smokers |
| Machion et al., 2006 | Single | Parallel | Atridox | 12m-22/19 24m- 22/17 | 40.45±4.47 | NR | Smokers | SRP alone | 12m-21/16 24m- 21/13 | 42.00±4.38 | NR | Smokers |
| Meinberg et al., 2002 | Single | Parallel | Arestin | 24/24 | 55.5±2.3 | 54.2 | 37.5 | SRP alone | 24/24 | 56.8±2.3 | 54.2 | 25 |
| Newman et al., 1994; Wilson et al., 1997 | Single | Split | Actisite | 113/105 | 51.0 (NR) | 46.7 | NR | SRP alone | 113/105 | 51 | 46.7 | NR |
| OPI study (103A) 2000 | Single | Parallel | Arestin | 121/115 | 48.6 ± 10.1 (29-76) | 43 | 42.1 | SRP alone | 124/114 | 48 ±10 (31-76) | 46 | 40.3 |
|  |  |  |  |  |  |  |  | SRP+vehicle | 123/112 | 47.7 ±9.7  (29-77) | 45.5 | 40.7 |
| OPI study (103B) 2000 | Single | Parallel | Arestin | 128/122 | 49.6 ± 10.2 (29-75) | 49.2 | 30.5 | SRP alone | 126/115 | 47.4 ±9.5 (29-72) | 48.4 | 32.5 |
|  |  |  |  |  |  |  |  | SRP+vehicle | 126/118 | 46.7 ± 10.3 (29-79) | 38.9 | 31.7 |
| Reddy et al., 2016 | NR | Parallel | PerioCol-TC Tet.fibers | 16/16 | NR | NR | Non-smokers | SRP alone | 16/16 | NR | NR | Non-smokers |
| Singh et al., 2014 | NR | Split | Periodontal Plus AB | 41/35 | 20-50 | 51.4 | - | SRP alone | 41/35 | 20-50 | 51.4 | - |
| Soeroso et al., 2017 | Open blind | Parallel | Periocline | NR/42 | 43.67±6.88 (NR) | 78.6 | Non-smokers | SRP alone | NR/39 | 44.00±6.93 (NR) | 76.9 | Non-smokers |
| Sweatha et al., 2015 | Single | Split | Arestin | 18/NR | >30 | NR | NR | SRP alone | 18/NR | >30 | NR | NR |
| Tabenski et al., 2017 | Single | Parallel | Arestin | NR/15 | 56(48-630 | 26.7 | 6.7 | SRP alone | 18/15 | 57 (51-59) | 66.7 | 26.7 |
| Timmerman et al., 1996 | Double | Parallel | Dentomycin | 10/10 | NR | NR | NR | SRP+placebo | 10/10 | NR | NR | NR |
| Tomasi et al., 2008; Tomasi & Wennstrom, 2011 | Single | Parallel | Atridox | NR/19 | 52 (37-67) | 52.6 | 47.4 | SRP alone | NR/13 | 54 (32-70) | 53.8 | 46.1 |
| Tonetti et al., 2012 | Single | Parallel | Ligosan | 100/89 | 50.2 (NR) | 67.4 | 37 | SRP alone | 102/92 | 49.8 (NR) | 67.3 | 37 |
| Tonetti et al., 1998 | Single | Parallel | Actisite | 66/63 | 51.3±9 (NR) | 48.4 | 42.2 | SRP alone | 61/60 | 48±9.3 (NR) | 41 | 49.2 |
| Van Dyke et al.,2002 | Double | Parallel | Arestin | .12/12 | NR | NR | NR | SRP alone | .12/10 | NR | NR | NR |
| Van Steenberghe et al., 1999 | Double | Parallel | Mino ointment | 46/40 | 48±7 (35-64) | 54.3 | NR | SRP+vehicle | 47/42 | 44 ±7 (34-61) | 53.2 | NR |
| Williams et al., 2001 | Single | Parallel | Arestin | 249/237 | 49.1±10.2  (29-76) | 46.2 | 36.1 | SRP alone | 250/229 | 47.7±9.7  (29-76) | 47.2 | 36.4 |
|  |  |  |  |  |  |  |  | SRP+vehicle | 249/230 | 47.2±10  (29-79) | 42.1 | 36.1 |
| Wong et al., 1998; Wong et al., 1999 | NR | Split | Actisite | 30/30 | 42.7 (NR) | 36.7 | NR | SRP alone | 30/30 | 42 .7 (NR) | 36.7 | NR |
| Zingale et al., 2012 | Double | Split | Arestin | 25/25 | 50.9 (31-76) | 52 | 4 | SRP alone | 25/25 | 50.9 NR (31-76) | 52 | 4 |

NR, not reported; SRP, scaling and root planing

Supplementary Table 3. Periodontal therapies: before and after inclusion.

| **Study reference** | **PT before inclusion** | **PT after inclusion** | **SRP treatment** | **Other Rx. during the follow-up period** |
| --- | --- | --- | --- | --- |
| Aboelsaad et al., 2014 | No PT in 6m | OHI, maintenance visits every 2wk – 2m once/m - 4m | FMSRP | No brushing - 12 h, no hard/sticky food - 1 wk, no interproximal cleaning – 10 d, no AB, no anti-inflammatory |
|  | No PT in 6m | OHI & FMSRP | Local SRP (study sites) | No OH in Rx areas - 1 wk |
| Ahamed et al., 2013 | No PT in 6m | OHI | FMSRP | No brushing/floss Rx sites - 7d, no MW, no AB |
| Aimetti et al., 2004 | No PT in 3m (SRP in ≥3m) | OHI &oral rinsing, maintenance scaling 3, 6, 9 m post-Rx | Local SRP (study sites) | Rinse with 15ml 0.2% CHX b.d - 2 min - 10±2 d. Refrain from brushing sites - 10±2 d |
| Bogren et al., 2008 | No PT in 3m (in SPT≥1y) | OHI & SRT every 6 m | FMSRP | No interdental OH - 1wk, 0.1% CHX b.d - 1 min - 1 m |
| Cortelli et al., 2006 | No PT in 12m | NR | FMSRP | NR |
| Dannewitz et al., 200) | No PT in 3-6m (SPT≥2y) | SRP at 3,6 &12 m after BL | FMSRP (PPD>4 mm) | NR |
| Deo et al.,2011 | NR | OHI Re-evaluation in 6 wk by measuring PBI and PI | Local SRP (study sites) | NR |
| Drisko et al., 1995 | Untreated | No SPT Scaling on d 10 | Local SRP (study sites) | NR |
| Eickholz et al., 2002; Ratka-Krüger et al., 2005 | Untreated / recurrent | OHI & FMSRP Maintenance at 3mth | Local and neighbouring site SRP_Local SRP | To use tooth brush gently, Refrain from interdental hygiene - 1 wk |
| Flemmig et al., 1996 | No PT in 3-6m (SPT every 3-6m) | OHI | FMSRP | Rinse with 15ml 0.2% CHX b.d – 10 d. Refrain from brushing test sites. |
| Friesen et al., 200) | NR | OHI & full mouth PMPR_supra & polishing | Local SRP | NR |
| Gonçalves et al., 2004; Colombo et al., 2003; Rodrigues et al., 2004 | No PT in 6m | OHI, supragingival prophylaxis, maintenance therapy/m | FMSRP | Rinsing with 0.12% CHX |
| Goodson et al., 1985 | Untreated | No OHI | Local SRP (study quadrant) | NR |
| Goodson et al.,2012; Socransky et al., 2013 | No PT in 3m | OHI with powered tooth brush | FMSRP | 0.12% CHX mouth rinse - 1 m during SRP |
| Gopinath et al., 2009 | No PT in 6m | OHI, no mouth wash use | Local SRP | Refrain from brushing or flossing area - 10 days |
| Henderson et al., 2002 | No PT in 6m | OHI | FMSRP | No interproximal cleaning - 3 d, brush and floss, no MW |
| Jain et al., 2012 | Untreated | OHI and full mouth supragingival scaling 1 wk before | FMSRP | No brushing -12 h |
| Jones et al., 1994 | No PT in 12m (comprehensive) | OHI | Local SRP (study sites) | No flossing - 2 wk in study quadrants |
| Killeen et al., 2016 | No PT in 6m (SPT every 6m) | NR | FMSRP (students) Local sites (hygienist) | Avoid flossing, tooth picks or hard crunching or sticky food in the Rx area - 7-10 d |
| Kinane & Radvar, 1999 | No PT in 6m SRP (>6m) | NR | Local SRP | NR |
| Lie et al., 1998 | Untreated | OHI | Local SRP (study sites, twice) | Proximal cleaning with toothpicks after 1d |
| Machion et al., 2004 | No PT in 6m | OHI, PMPR_supra | Local SRP (study sites, | No floss - 7 d in Rx area |
| Machion et al., 2006 | No PT in 6m | OHI, PMPR_supra | Local SRP (study sites, | No floss - 7 d in Rx area |
| Meinberg et al., 2002 | No PT in 6m | OHI | Local SRP (study sites, | NR |
| Newman et al., 1994; Wilson et al., 1997 | Regular SPT | NR | FMSRP | No brushing Rx sites, no crusty food - 10 d |
| OPI (103A) 2000 | No PT in 6m | NR | NR | NR |
| OPI (103B) 2000 | No PT in 6m | NR | NR | NR |
| Oringer et al., 2002 | NR | NR | FMSRP | NR |
| Reddy et al., 2016 | No PT in 3 m | OHI, PMPR | FMSRP | Not disturbing the area, no hard/sticky food - 1 wk, No brushing/flossing – 1 wk |
| Singh et al., 2014 | NO PT in 6m | OHI & PMPR_supra, SPT 1, 3, 6m | FMSRP | NR |
| Soeroso et al., 201) | No PT in 6m | OHI | FMSRP | - |
| Sweatha et al., 2015 | No PT in 6m | OHI | Local SRP sites | Avoid hard food - 1 wk, avoid interproximal cleaning - 10 d |
| Tabenski et al., 2017 | No PT in 3m | OHI & PMPR_supra, SPT 6w, 3, 6, 9 and 12 m | FMSRP | Rinse with 0.2% CHX 1 min, b.d, - 5 d. No flossing - 10 d |
| Timmerman et al., 1996 | No PT in 12m | OHI, PMPR | FMSRP | - |
| Tomasi et al., 2008; Tomasi & Wennstrom, 2011 | No PT in 12m | OHI & PMPR_supra 1m, 3m | FMSRP (PPD>4mm, furcation) | No interproximal cleaning - 1 wk in Rx area |
| Tonetti et al., 1998 | No PT in 3-6 m (SPT in 3-6m) | OHI | Local SRP | NR |
| Tonetti et al., 2012 | No PT in 6m | OHI SPT every 3m | FMSRP (PPD>4mm) | Avoid interdental cleaning – 36 h |
| Van Dyke et al., 200) | NR | OHI | Local SRP | NR |
| Van Steenberghe et al., 1999 | NR | NR | FMSRP | Avoid oral irrigation devices |
| Williams et al., 2001 | No PT in 6m | NR | FMSRP | NR |
| Wong et al., 1998; Wong et al., 1999 | SPT | NR | Local SRP | Avoid brushing, Rx sites, CHX MW - 10 d |
| Zingale et al., 2012 | SPT | OHI | Local SRP (2quadrants) | Avoid brushing/flossing, Rx sites – 14 d. CHX MW (0.122%), b.d, - 14 d |

b.d., twice a day; CHX, chlorhexidine; FMSRP, full-mouth scaling and root planing; MW, mouth wash; NR, not reported; OHI, instructions in oral hygiene; PMPR, professional mechanical plaque removal; PPD, probing pocket depth; PT, periodontal therapy; Rx, treatment; SPT, supportive periodontal therapy; SRP, scaling and root planing; sub, subgingival; supra, supragingival; min, minutes; h, hours; d, days; wk, week; m, months.

Supplementary Table 4. Periodontitis features and evaluated teeth/sites criteria

| **Study reference** | **Extension** | **Type** | **Severity** | **FM/PM** | **Criteria used to select treatment** |
| --- | --- | --- | --- | --- | --- |
| Aboelsaad et al., 2014 | NR | Chronic | Mod-sev | PM- 2 sites | PPD≥5mm, CAL> 4mm, and radio evidence of bone loss |
| Agan et al., 2006 | NR | Chronic | NR | PM- 2 sites | PPD≥6mm, CAL>3mm & BOP Single-rooted, non-adjacent |
| Ahamed et al., 2013 | NR | Chronic (adult) | NR | PM- 5 sites | PPD 6-7mm, non-adjacent |
| Aimetti et al., 2004 | Localized | Recurrent (persistent) | NR | PM- 2 teeth | PPD 4-5mm & BOP Non-furcation, non-adjacent |
| Bogren et al., 2008 | NR | Recurrent | Mod-adv | FM- PD>4 | PPD≥5mm |
| Cortelli et al., 2006; Cortelli et al., 2008 | NR | Chronic (adult) | Adv | PM- 2 sites | PPD≥6mm, non-molar sites, no furcation |
| Dannewitz et al., 2009 | NR | Recurrent | Mod-sev | PM- all furcation lesions | PPD 5-7 mm, BOP, furcation sites |
| Deo et al., 2011 | Generalized | Chronic | NR | PM- 6 sites | PPD> 5mm, BOP, molar |
| Drisko et al., 1995 | Few generalized | Adult | Mod-sev | FM | PPD≥ 5 mm and BOP, non-adjacent |
| Eickholz et al., 2002; Ratka-Krüger et al., 2005 | NR | Untreated / recurrent | Mod-sev | PM- 4-6 site | PPD≥5mm & BOP / PPD≥6mm, single-rooted teeth |
| Flemmig et al., 1996 | Localized | Recurrent | NR | PM- 1 tooth 6 sites/tooth | PPD≥5mm & BOP |
| Friesen et al., 2002 | NR | Periodontitis | NR | PM- 1 tooth | PPD 6-10mm & BOP |
| Gonçalves et al., 2004; Colombo et al., 2003; Rodrigues et al., 2004 | NR | Chronic (adult) | NR | FM/PM - 4 sites | PPD>6mm (PM) non-adjacent sites, no furcation |
| Goodson et al., 1985 | NR | NR | NR | FM | CAL≥2mm |
| Goodson et al.,2012; Socransky et al., 2013 | NR | NR | NR | FM | PPD> 5 mm & CAL> 3mm all teeth except third molars |
| Gopinath et al.,2009 | NR | Chronic | NR | PM- 4 sites 2 -6 teeth | PPD 5-8mm, radiographic evidence of alveolar bone loss |
| Henderson et al., 2002 | NR | Chronic (adult) | Mod-sev | PM- 1 site | PPD 6-9mm, CAL≥3 mm, no furcation |
| Jain et al., 2012 | NR | Chronic | Mod-sev | PM 1-2 sites | PPD> 5mm; CAL> 4mm |
| Jones et al., 1994 | NR | Adult | Mod-adv | PM- 13 site (average) | PPD≥ 7mm & culture |
| Killeen et al., 2016 | NR | Recurrent | Mod-sev | PM- 1 site | PPD≥ 5mm & BOP, posterior inter-proximal |
| Kinane & Radvar, 1999 | NR | Recurrent | NR | PM- 1 site | PPD≥ 5mm & BOP &/or suppuration Non-adjacent, no furcation |
| Lie et al., 1998 | NR | Chronic (adult) | Mod-sev | PM- 1 site | PPD≥ 5mm & BOP single-rooted |
| Machion et al., 2004 | NR | Chronic | NR | PM- 6 site | PPD≥ 5mm & BOP, single-rooted teeth (anteriors) |
| Machion et al., 2006 | NR | Chronic | NR | PM- 6 site | PPD≥ 5mm & BOP, single-rooted teeth (anteriors) |
| Meinberg et al., 2002 | NR | Chronic | Mod-adv | PM-2 sites | PPD PD≥ 5mm & BOP (PM & M inter-proximal) |
| Newman et al., 1994; Wilson et al., 1997 | Localized | Recurrent | NR | PM- -2sites | PPD 5-8mm & BOP |
| OPI study (103A) 2000 | NR | Chronic | Mod-adv | FM-PPD≥ 5mm | PPD 6-9mm and BOP |
| OPI study (103B) 2000 | NR | Chronic | Mod-adv | FM-PPD≥ 5mm | PPD 6-9mm and BOP |
| Oringer et al., 2002 | NR | Chronic | Mod-sev | PM | PPD >5 mm |
| Reddy et al., 2016 | NR | Recurrent | NR | PM- 1 site | PPD≥5mm & BOP/suppuration in non-adjacent teeth, no furcation. |
| Singh et al., 2014 | NR | Chronic | NR | FM | PPD 5-8mm, CAL >3mm & BOP |
| Soeroso et al., 2017 | Localized | Chronic (adult) | Mod-sev | FM | PPD4-6mm, radiographic evidence ≥4mm & BOP |
| Sweatha et al., 2015 | NA | Chronic | NA | PM-4 | PPD≥5mm; radiographic evidence of bone loss. |
| Tabenski et al., 2017 | Generalized | Chronic (adult) | Mod-sev | PM- 4 teeth | PPD≥6mm & BOP |
| Timmerman et al., 1996 | NR | Chronic (adult) | Mod-sev | FM/PM- 4-10 sites | PPD≥5mm & CAL ≥3mm & alveolar bone loss |
| Tomasi et al., 2008; Tomasi & Wennstrom, 2011 | NR | Chronic (adult) | Mod-adv | FM/PM- all furcation lesions | PPD≥5mm & BOP FM, all teeth except third molars / PM, teeth associated with furcation involvement |
| Tonetti et al., 1998 | NR | Recurrent | NR | PM- 1 furcation lesion | Mandibular class II furcation with BOP |
| Tonetti et al., 2012 | NR | Recurrent | Mod-sev | FM- PPD >3 | PPD≥4mm & BOP |
| Van Dyke et al.,2002 | NR | Periodontitis | Mod-sev | PM- 2 teeth | PPD≥5mm & PGE2 levels>66.2ng/ml in GCF |
| Van Steenberghe et al., 1999 | NR | Chronic (Adult) | Mod-sev | PM – 6 sites | PPD≥5mm, CAL P≥3mm, radiographic evidence of bone loss |
| Williams et al., 2001 | NR | Chronic (adult) | Mod-sev | FM- PPD >4 | PPD ≥ 5mm (6-9mm) & BOP |
| Wong et al., 1998; Wong et al., 1999 | Localized | Recurrent | NR | PM - 1-2 sites | PPD 4-8mm & BOP,  AST >800mIU |
| Zingale et al., 2012 | NR | Chronic | Mod-adv | PM - 1 site | PPD 5-9mm & BOP |

adv, advanced; AST, aspartate aminotransferase; BOP, bleeding on probing; CAL, clinical attachment level; FM, full-mouth (all sites, specific criteria); GCF, gingival cervical fluid; Mod, moderate; NR, not reported; PM, partial-mouth (selected sites); PPD, probing pocket depth; PGE2, prostaglandin E2; sev, severe.

Supplementary Table 5. Clinical outcome assessment and other outcomes.

| **Study reference** | **N groups** | **Sites/ tooth** | **Number of examiners** | **Calibration** | **Probing type** | **Probe/Stent** | **Statistical unit** | **Other outcomes measured** |
| --- | --- | --- | --- | --- | --- | --- | --- | --- |
| Aboelsaad et al., 2014 | 2 | 1 | 1 | Yes | MA | Colored Peri-probe PQWBR | Site-based | NR |
| Agan et al., 2006 | 4 | 6 | NR | NR | NR | NR | Subject-based | Biomarkers (MMP-8) |
| Ahamed et al.,2013 | 2 | NR | NR | NR | MA | Probe (1mm interval) & stent | Site-based | Micro |
| Aimetti et al., 2004 | 2 | 6 | 1 | NR | MA | Williams | Site-based | Micro & radio |
| Bogren et al., 2008 | 2 | 4 | 2 | Yes | MA | UNC 15/ duplicate | Subject-based | Micro |
| Cortelli et al., 2006; Cortelli et al., 2008 | 2 | 6 | 2 | Yes | MA | Periodontal probe (PQWBR) | NR | No |
| Dannewitz et al., 2009 | 2 | 6 | 1 | NR | MA | PCP-UNC 15 | Subject-based | No |
| Deo et al.,2011 | 2 | 6 | 1 | NR | MA | Williams | Subject-based | No |
| Drisko et al., 1995 | 4 | 3 | 3 | Yes | MA | UNC 15/ duplicate | Subject-based | NR |
| Eickholz et al., 2002; Ratka-Krüger et al., 2005 | 3 | 4-6 | 6 | Yes | MA | UNC 15/ stent &duplicate | Subject-based | Micro |
| Flemmig et al., 1996 | 2 | 6 | 1 | NR | FC & CA | Florida/ duplicate | Site-based | Biomarkers |
| Friesen et al., 2002 | 4 | 6 | NR | NR | MA | UNC 15 | Subject-based | AB concentration, GCF volume |
| Gonçalves et al., 2004; Colombo et al., 2003; Rodrigues et al., 2004 | 3 | 6 | 2 | Yes | MA | UNC 15 | Subject-based | Micro |
| Goodson et al., 1985 | 4 | 6 | 1 | NR | MA | Michigan ‘O’ probe | NR | Bioassay |
| Goodson et al.,2012; Socransky et al., 2013 | 8 | 6 | 2 | Yes | MA | UNC/ duplicate | Subject-based | No |
| Gopinath et al., 2009 | 2 | 4 | NR | NR | NR | Stent | NR | No |
| Henderson et al., 2002 | 2 | 1 | 1 | NR | MA | Calibrated probe with 1mm increment | Subject-based | No |
| Jain et al., 2012 | 2 | NR | NR | NR | MA | Stent and endodontic spreader | Site-based | Micro |
| Jones et al.,1994 | 4 | 6 | NR | NR | FC | Florida | Subject-based | Micro |
| Killeen et al., 2007 | 2 | 1 | 2 | Yes | MA | UNC 15 | Subject-based | Biomarkers (Ratio ofIL-1b/IL-1R antagonist) |
| Kinane & Radvar, 1999 | 4 | 4 | 1 | NR | FC & CA | Florida probe/stent duplicate | Subject-based | No |
| Lie et al., 1998 | 3 | 2 | 1 | NR | FC & CA | Florida probe | Subject-based | Micro |
| Machion et al., 2004 | 2 | 6 | 1 | Yes | FC & CA | Florida probe/stent | Subject-based | No |
| Machion et al., 2006 | 2 | 6 | 2 | Yes | FC & CA | Florida probe/stent | Subject-based | No |
| Meinberg et al., 2002 | 2 | 2 | 1 | Yes | NR | NR | Subject-based | Radio |
| Newman et al., 1994; Wilson et al., 1997 | 2 | 2 | 7 | Yes | FC & CA | Florida | Subject-based | Micro |
| OPI (103A) 2000 | 3 | NR | 8 | Yes | MA | NR | Subject-based | No |
| OPI (103B) 2000 | 3 | NR | 8 | Yes | MA | NR | Subject-based | No |
| Oringer et al., 2002 | 3 | 6 | 18 | Yes | MA | UNC15/ duplicate | Subject-based | No |
| Reddy et al., 2016 | 3 | NR | 1 | NR | MA | UNC 15 | NR | No |
| Singh et al., 2014 | 3 | 4 | NR | NR | FC & CA | Florida | NR | No |
| Soeroso et al., 2017 | 2 | 2 | NR | NR | NR | NR | NR | Micro |
| Sweatha et al., 2015 | 2 | 6 | NR | NR | MA | UNC-15/stent | NR | No |
| Tabenski et al., 2017 | 3 | 6 | 3 | Yes | FC | Borodontic | Subject-based | Micro |
| Timmerman et al., 1996 | 2 | 6 | 1 | NR | MA | 15mm et al, 0.56 interval/ Stent | Subject & site-based | Micro |
| Tomasi et al., 2008; Tomasi & Wennstrom, 2011 | 2 | 6 Max.mo -9 Man.mo-12 | 2 | Yes | MA | PCP-15/ duplicate | Subject & site-based | Radio |
| Tonetti et al., 1998 | 2 | 1 | 1 | Yes | FC | Pressure sensitive probe | Subject-based | No |
| Tonetti et al., 2012 | 2 | 6 | 5 | NR | FC | Borodontic | NR | Micro |
| Van Dyke et al., 2002 | 4 | 6 | NR | NR | FC&CA | Florida | Subject-based | Biomarkers & systemic |
| Van Steenberghe et al., 1999 | 2 | 6 | NR | NR | MA | 15mm et al,0.56 interval/stent | Subject-based | Micro & radio |
| Williams et al., 2001 | 3 | 6 | 18 | Yes | MA | UNC-15 & duplicate | Subject-based | Systemic |
| Wong et al., 1998; Wong et al., 1999 | 2 | NR | NR | NR | FC | Interprobe | Subject-based | Micro & biomarkers |
| Zingale et al., 2012 | 4 | NR | 1 | Yes | MA | UNC-15 | Subject-based | No |

AB, antibiotic; CA, computer-assisted; FC, force-controlled; GCF, gingival crevicular fluid; MA, manual; Micro, microbiological; MMP, matrix metalloproteinase; NR, not reported; PCP, periodontal color-coded probe; Radio, radiological; UNC, University of North Carolina.

Supplementary Table 6. Methodology used to measure PI, GI and BOP.

| **Study reference** | **PI reference** | **GI reference** | **BOP** |
| --- | --- | --- | --- |
| Aboelsaad et al., 2014 | Silness & Lӧe, 1964 | NR | Lang et al., 1991 |
| Agan et al., 2006 | Quigley & Hein, 1962 | NR | Probe bleeding index (PBI) Saxer et al., 1975 |
| Ahamed et al., 2013 | Silness & Lӧe, 1964 | Lӧe & Silness, 1963 | Ainamo & Bay, 1975 |
| Aimetti et al., 2004 | Dichotomous | NR | Dichotomous |
| Bogren et al.,2008 | Dichotomous | NR | Dichotomous |
| Cortelli et al., 2006 | NR | NR | NR |
| Dannewitz et al., 2009 | O’Leary et al., 1972 | NR | Dichotomous |
| Deo et al., 2011 | Turesky et al., 1990 | NR | Periodontal bleeding index Mulhelmann HR, 1977 |
| Drisko et al., 1995 | Lӧe et al., 1978 | NR | Dichotomous |
| Eickholz et al., 2002; Ratka-Krüger et al., 2005 | Lӧe, 1967 | NR | NR |
| Flemmig et al., 1996 | Silness & Lӧe, 1964 | Lӧe & Silness, 1963 | NR |
| Friesen et al., 2002 | Silness & Lӧe, 1964 | Lӧe & Silness, 1963 | Dichotomous |
| Gonçalves et al., 2004; Colombo et al., 2003; Rodrigues et al., 2004 | Dichotomous | NR | NR |
| Goodson et al., 1985 | NR | NR | NR |
| Goodson et al.,2012; Socransky et al., 2013 | Dichotomous | NR | Dichotomous |
| Gopinath et al., 2009 | Carranza and Newman, 1996 | Carranza and Newman, 1996 | Carranza and Newman, 1996 |
| Henderson et al., 2002 | Silness & Lӧe, 1964 | NR | Dichotomous |
| Jain et al., 2012 | Silness & Lӧe, 1964 | NR | Ainamo & Bay, 1975 |
| Jones et al., 1994 | Silness & Lӧe, 1964 | Lӧe & Silness, 1963 | Dichotomous |
| Killeen et al., 2016 | NR | NR | NR |
| Kinane & Radvar, 1999 | Silness & Lӧe, 1964 | Modified gingival index Lobene et al., 1986 | Dichotomous |
| Lie et al., 1998 | NR | NR | Dichotomous |
| Machion et al., 2004 | NR | NR | NR |
| Machion et al., 2006 | Dichotomous | NR | Dichotomous |
| Meinberg et al., 2002 | NR | NR | NR |
| Newman et al., 1994; Wilson et al., 1997 | NR | NR | NR |
| OPI study (103A) 2000 | NR | NR | Dichotomous |
| OPI study (103B) 2000 | NR | NR | Dichotomous |
| Oringer et al., 2002 | NR | NR | NR |
| Reddy et al., 2016 | NR | NR | NR |
| Singh et al., 2014 | NR | NR | Dichotomous |
| Soeroso et al., 2017 | Lӧe & Silness, 1963 | NR | Sulcular bleeding index Muhlemann & Son, 1971 |
| Sweatha et al., 2015 | Silness & Lӧe, 1964 | Lӧe & Silness, 1963 | Papillary bleeding index Mulhelmann HR, 1977 |
| Tabenski et al., 2017 | Approximal plaque index Lange et al., 1977 | - | Bleeding on Probing Lang et al., 1986 |
| Timmerman et al., 1996 | Silness & Lӧe, 1964 | Lӧe & Silness, 1963 | Papillary bleeding index Mulhelmann HR, 1977 |
| Tomasi et al., 2008; Tomasi & Wennstrom, 2011 | Dichotomous | NR | Dichotomous |
| Tonetti et al., 1998 | O’Leary, 1972 | NR | Lang et al., 1990 |
| Tonetti et al., 2012 | O’Leary, 1972 | NR | Dichotomous |
| Van Dyke et al., 2002 | Silness & Lӧe, 1964 | Lӧe & Silness (1963) | NR |
| Van Steenberghe et al., 1999 | Silness & Lӧe, 1964 | Lӧe & Silness (1963) | Mulhelmann HR, 1977 |
| Williams et al., 2001 | NR | NR | Dichotomous |
| Wong et al., 1998; Wong et al., 1999 | Silness & Lӧe, 1964 | NR | NR |
| Zingale et al., 2012 | NR | NR | Dichotomous |

NR, not reported

Supplementary Table 7. Root debridement characteristics.

| **Study reference** | **Local anesthesia** | **Number of operators** | **Operator type** | **Debridement time** | **Instruments used for debridement** |
| --- | --- | --- | --- | --- | --- |
| Aboelsaad et al., 2014 | NR | 1 | NR | NR | Hand and ultrasonic |
| Agan et al., 2006 | Yes | NR | NR | 10 min/tooth | Hand |
| Ahamed et al., 2013 | NR | NR | NR | NR | Hand and ultrasonic |
| Aimetti et al., 2004 | Yes | 1 | NR | NR | NR |
| Bogren et al., 2008 | If requested | 2 | NR | No restriction | Hand and ultrasonic |
| Cortelli et al., 2006 | Yes | NR | NR | NR | NR |
| Dannewitz et al., 2009 | NR | 1 | Periodontist | NR | Sonic scaler (sonicflex) |
| Deo et al., 2011 | Yes | 1 | NR | NR | Hand |
| Drisko et al., 1995 | Yes | 3 | NR | 5 min/tooth | NR |
| Eickholz et al., 2002; Ratka-Krüger et al., 2005 | Yes | 6 | NR | 10min/tooth | Hand |
| Flemmig et al., 1996 | NR | 1 (fiber) | Dentist (fiber) | NR | NR |
| Friesen et al., 2002 | Yes | NR | NR | 5min/tooth | NR |
| Gonçalves et al., 2004; Colombo et al., 2003; Rodrigues et al., 2004 | Yes | 2 | Periodontist | Full mouth 1h | NR |
| Goodson et al.,1985 | NR | 1 | NR | >45min/quadrant | NR |
| Goodson et al.,2012; Socransky et al., 2013 | Yes | >2/center | NR | NR | NR |
| Gopinath et al.,2009 | NR | NR | NR | NR | NR |
| Henderson et al., 2002 | If indicated | 1 | Dentist | Full mouth 90 min | Hand and ultrasonic |
| Jain et al., 2012 | NR | NR | NR | NR | NR |
| Jones et al., 1994 | NR | NR | NR | NR | NR |
| Killeen et al., 2016 | NR | 2 | Dental student (FMSRP), Dental hygienist (local sites) | <5min | NR |
| Kinane & Radvar, 1999 | NR | 1 | NR | NR | NR |
| Lie et al., 1998 | NR | 1 | NR | NR | Hand and ultrasonic |
| Machion et al., 2004 | NR | 1 | NR | NR | NR |
| Machion et al., 2006 | NR | 1 | NR | NR | NR |
| Meinberg et al., 2002 | No | 1 | Dental hygienist | >1h – BL, <5min/appointment | Hand and ultrasonic |
| Newman et al., 1994; Wilson et al., 1997 | NR | 7 | Therapist | NR | NR |
| OPI (103A) 2000 | NR | NR | NR | No restriction | NR |
| OPI (103B) 2000 | NR | NR | NR | No restriction | NR |
| Oringer et al., 2002 | Yes | NR | NR | No restriction | NR |
| Reddy et al., 2016 | Yes | NR | NR | NR | Hand and ultrasonic |
| Singh et al., 2014 | Yes | NR | NR | NR | NR |
| Soeroso et al., 2017 | NR | NR | NR | NR | NR |
| Sweatha et al., 2015 | NR | NR | NR | NR | NR |
| Tabenski et al., 2017 | Yes | 3 | Dentists | NR | Hand and ultrasonic |
| Timmerman et al., 1996 | If necessary | NR | Dental hygienist | 3 min/tooth, 15min/quadrant, 3h/pt | Hand |
| Tomasi et al., 2008; Tomasi & Wennstrom, 2011 | If requested | 1 | Dental hygienist | No restriction | Ultrasonic |
| Tonetti et al., 2012 | NR | 5 | NR | No restriction | Ultrasonic/sonic |
| Tonetti et al., 1998 | NR | 6 | NR | 3min/tooth | Sonic and hand |
| Van Dyke et al., 2002 | NR | NR | NR | NR | NR |
| Van Steenberghe et al., 1999 | If necessary | NR | NR | 15min/quadrant | NR |
| Williams et al., 2001 | As needed | NR | NR | No restriction | NR |
| Wong et al., 1998; Wong et al., 1999 | NR | NR | NR | NR | NR |
| Zingale et al., 2012 | Yes | 2 | Periodontist | No restriction | NR |

BL, baseline; NR, not reported; pt, patient; min, minutes; h, hours

Supplementary Table 8. Frequency and time of local antimicrobial application.

| **Study reference** | **Number of applications** | **Time of administration** | **Dressing** | **Dislodge record** |
| --- | --- | --- | --- | --- |
| Aboelsaad et al., 2014 | 1 | BL | NR | NR |
| Agan et al., 2006 | 1 | BL | Peripac - 7d | NR |
| Ahamed et al., 2013 | 1 | BL | Periodontal dressing - 7d (10±2d) | Yes |
| Aimetti et al., 2004 | 1 | BL | Cyanoacrylate-10±2d | Yes |
| Bogren et al., 2008 | 3 | BL 1y, 2y | No | - |
| Cortelli et al., 2006 | 4 | BL, 3m, 6m, 9m | No | NR |
| Dannewitz et al., 2009 | 1 | BL | No | NR |
| Deo et al., 2011 | 1 | BL | NR | NR |
| Drisko et al., 1995 | 1 (2) | BL (if dislodged) | Cyanoacrylate - 10±2d | Yes |
| Eickholz et al., 2002; Ratka-Krüger et al., 2005 | 1 | BL | No | Yes |
| Flemmig et al., 1996 | 1 | 1 w after FMSRP | Cyanoacrylate - 10d | Yes |
| Friesen et al., 2002 | 1 | BL | Cyanoacrylate - 7-10d | Yes |
| Gonçalves et al., 2004; Colombo et al., 2003; Rodrigues et al., 2004 | 1 | BL | No | NR |
| Goodson et al., 1985 | 1 | BL | Periodontal dressing -10d | NR |
| Goodson et al.,2012; Socransky et al., 2013 | 1 | BL | No | NR |
| Gopinath et al., 2009 | 2 | BL, 30 d | NR | NR |
| Henderson et al., 200) | 1 | BL | NR | NR |
| Jain et al., 2012 | 3 | BL, 2w, 4w | Cyanoacrylate | NR |
| Jones et al.,1994 | 1 | BL | NR | NR |
| Killeen et al., 2016 | 2 | BL, 6m | No | - |
| Kinane & Radvar, 1999 | Dentomycin - 3 | BL,2w,4w | No | - |
|  | Actisite - 1 | BL | NR |  |
| Lie et al., 1998 | 2 | BL, 1w | No | - |
| Machion et al., 2004 | 1 | BL | NR | NR |
| Machion et al., 2006 | 2 | BL, 12m | NR | NR |
| Meinberg et al., 2002 | 4 | BL, 1m, 3m, 6m | NR | NR |
| Newman et al., 1994; Wilson et al., 1997 | 1 | BL | Cyanoacrylate 10±2 d | Yes |
| OPI (103A) 2000 | 1 | BL | NR |  |
| OPI (103B) 2000 | 1 | BL | NR |  |
| Oringer et al., 2002 | 3 | BL, 3m, 6m | NR | NR |
| Reddy et al., 2016 | 1 | BL | No | - |
| Singh et al., 2014 | 2 | BL, 1m | Perio pack | NR |
| Soeroso et al., 2017 | 4 | BL, 1w, 2w, 3w | No | - |
| Sweatha et al., 2015 | 1 | BL, 1m | No | - |
| Tabenski et al., 2017 | 1 | BL | No | - |
| Timmerman et al., 1996 | 7 | BL, 2w, 1m, 3m, 6m, 9m, 12m | No | - |
| Tomasi et al., 2008; Tomasi & Wennstrom, 2011 | 1 | BL | No | - |
| Tonetti et al., 1998 | 1 (2) | - | Cyanoacrylate-10±3d | Yes |
| Tonetti et al., 2012 | 1 | BL | No | - |
| Van Dyke et al., 2002 | 1 | BL | No | - |
| Van Steenberghe et al., 1999 | 7 | BL, 2w, 1m, 3m, 6m, 9m, 12m | NR | NR |
| Williams et al., 2001 | 3 | BL, 3m, 6m | No | - |
| Wong et al., 1998; Wong et al., 1999 | 1 (2) | BL (during first 10d, if lost) | Cyanoacrylate-10d | Yes |
| Zingale et al., 2012 | 1 | BL | No | - |

BL, baseline; d, days; FMSRP, full-mouth scaling and root planing; NR, not reported; d, days; w, weeks; m, months; y, years.

Supplementary Table 9. Risk of bias assessment of the individual studies.

| **Study reference** | **Selection bias** | | **Performance bias (blinding of participants and personnel)** | **Detection bias (blinding of outcome assessment)** | **Attrition bias (incomplete outcome data)** | **Reporting bias (selective reporting)** | **Global RoB** |
| --- | --- | --- | --- | --- | --- | --- | --- |
|  | **Random sequence generation** | **Allocation concealment** |  |  |  |  |  |
| Aboelsaad et al., 2014 | UR | UR | UR | L | UR | L | H |
| Agan et al., 2006 | UR | UR | UR | UR | UR | UR | H |
| Ahamed et al., 2013 | UR | H | H | H | L | UR | H |
| Aimetti et al., 2004 | UR | UR | UR | L | L | UR | H |
| Bogren et al., 2008 | L | UR | UR | L | UR | UR | H |
| Cortelli et al., 2006 | UR | H | L | L | UR | UR | H |
| Dannewitz et al., 2009 | L | L | L | UR | UR | L | H |
| Deo et al., 2011 | UR | H | UR | UR | L | UR | H |
| Drisko et al., 1995 | L | UR | L | L | UR | L | H |
| Eickholz et al., 2002; Ratka-Krüger et al., 2005 | L | L | L | UR | L | L | M |
| Flemmig et al., 1996 | UR | UR | UR | UR | UR | UR | H |
| Friesen et al., 2002 | L | UR | UR | UR | L | L | H |
| Gonçalves et al., 2004; Colombo et al., 2003; Rodrigues et al., 2004 | L | UR | UR | H | H | L | H |
| Goodson et al., 1985 | L | UR | UR | UR | UR | UR | H |
| Goodson et al.,2012; Socransky et al., 2013 | UR | UR | H | L | L | H | H |
| Gopinath et al., 2009 | H | H | UR | H | H | UR | H |
| Henderson et al., 2002 | UR | UR | L | UR | L | UR | H |
| Jain et al., 2012 | UR | UR | H | UR | UR | UR | H |
| Jones et al., 1994 | UR | UR | L | UR | H | UR | H |
| Killeen et al., 2016 | UR | UR | L | UR | UR | L | H |
| Kinane & Radvar, 1999 | UR | UR | UR | UR | UR | L | H |
| Lie et al., 1998 | UR | UR | UR | L | UR | H | H |
| Machion et al., 2004 | UR | H | UR | UR | UR | UR | H |
| Machion et al., 2006 | UR | H | UR | UR | UR | UR | H |
| Meinberg et al., 2002 | UR | H | UR | UR | UR | L | H |
| Newman et al., 1994; Wilson et al., 1997 | L | UR | UR | UR | UR | UR | H |
| OPI (103A) 2000 | L | UR | UR | L | L | L | H |
| OPI (103B) 2000 | L | UR | UR | L | L | L | H |
| Oringer et al., 2002 | UR | H | UR | UR | H | UR | H |
| Reddy et al., 2016 | UR | H | H | UR | UR | UR | H |
| Singh et al., 2014 | UR | H | UR | UR | L | UR | H |
| Soeroso et al., 2017 | UR | H | H | UR | L | UR | H |
| Sweatha et al., 2015 | UR | H | UR | UR | UR | UR | H |
| Tabenski et al., 2017 | L | H | UR | UR | UR | L | H |
| Timmerman et al., 1996 | UR | H | L | UR | L | UR | H |
| Tomasi et al., 2008; Tomasi & Wennstrom, 2011 | L | L | UR | UR | UR | L | H |
| Tonetti et al., 1998 | UR | UR | UR | L | UR | UR | H |
| Tonetti et al., 2012 | L | L | UR | UR | L | UR | H |
| Van Dyke et al., 2002 | UR | H | L | UR | UR | L | H |
| Van Steenberghe et al., 1999 | L | L | L | UR | UR | L | M |
| Williams et al., 2001 | UR | UR | UR | L | UR | L | H |
| Wong et al., 1998; Wong et al., 1999 | UR | UR | UR | UR | L | L | H |
| Zingale et al., 2012 | UR | UR | L | L | UR | UR | H |

H, high; L, low; RoB, risk of bias; UR, unclear risk

Supplementary Table 10. Outcomes with significant differences in individual studies with adverse effects and exclusion criteria used

| **Study reference** | **Outcomes with significant differences >6 months** | **Adverse effects** | **Exclusion criteria** |
| --- | --- | --- | --- |
| Aboelsaad et al., 2014 | PPD | No | Periodontal Rx in last 6 months, Systemic diseases, Pregnant/lactation, Systemic AB, NSAIDS in last 3 months, Allergy |
| Agan et al., 2004 | No difference | No | Systemic AB-6 months, Mouth wash/irrigation 1 month, Pregnant/lactation, Allergy |
| Ahamed et al., 2013 | PPD, CAL, BOP | No | Compromised medical condition, Contraindicated medicine, Pregnant/lactation, AB last 6 months, Allergy |
| Aimetti et al., 2004 | PPD, CAL, BOP | NR | Systemic diseases, Pregnant/lactation, AB last 3 months, Allergy |
| Bogren et al., 2008 | No difference | No | Systemic diseases, Pregnant/lactation, AB prophylaxis, Allergy, AB 3 months, Drugs that could influence outcome |
| Cortelli et al., 2006 | No difference | NR | DM, Immunocompromised patients, Pregnant/lactation |
| Dannewitz et al., 2009 | No difference | NR | Allergy, Alcohol abuse, Chronic diseases, Local/systemic AB-6 months, Psychological disorders, Anti-coagulants |
| Deo et al., 2011 | PPD, CAL | No | No systemic diseases, AB/anti-infl. for 6 months, Allergy, Pregnant/lactating, No MW |
| Drisko et al., 1995 | No difference | 12 Abscess -1, oral candidiasis-3, severe gingival redness -3, tongue pigmentation-3, glossitis-1 | AB premedication, Pregnant/lactation, AB 3 months, Candidiasis, Allergy |
| Eickholz et al., 2002; Ratka-Krüger et al., 2005 | PPD, CAL, GI | 2 minor complaints; inflammation. | Local or Systemic AB last 6 months, Systemic diseases, Allergy, Pregnant/lactating, Anticoagulant therapy. |
| Flemmig et al., 1996 | PPD, GI | No | Systemic AB last 3 months, AB premedication, Perio. surgery last 6 months, Systemic Diseases, Allergy, Pregnant/lactating, Oral candidiasis |
| Friesen et al., 2002 | PPD, CAL | One. Bitter taste | Systemic AB last 3 months, AB premedication, Systemic diseases, Allergy, Pregnant/lactation, Oral candidiasis |
| Gonçalves et al., 2004; Colombo et al., 2003; Rodrigues et al., 2004 | CAL, BOP | NR | Systemic AB last 6 months, AB premedication, Systemic diseases, Allergy, Pregnant/lactation |
| Goodson et al., 1985 | No difference | NR | Allergy to tetracycline |
| Goodson et al.,2012; Socransky et al., 2013 | No difference | No | Pregnant/lactation, AB/surgery – 3 months, Systemic Diseases, AB premedication, Allergy |
| Gopinath et al., 2009 | PPD, GI, PI, GBI | NR | Long-term drug Rx – 1 month before, AB last 3 months, Systemic diseases, Allergy, Pregnant/lactation |
| Henderson et al., 2002 | No difference | No | AB prophylaxis, AB last 3 months/ DM, Immunocompromised, Allergy, Pregnant/lactation |
| Jain et al., 2012 | PPD | No | Allergy, Pregnant/lactation, AB/anti-inflammatory drugs/steroids -3 months, |
| Jones et al., 1994 | No difference | No | AB/NSAIDs last 3 months (drugs affecting perio. status), Systemic diseases, Allergy, Pregnant/lactation, AB prophylaxis |
| Killeen et al., 2007 | No difference | No | Systemic diseases, AB/NSAID & other long-term drugs, Surgical perio Rx.-1year, Pregnant/lactation, Allergy |
| Kinane & Radvar, 1999 | PPD | NR | Systemic diseases, Systemic AB -6 months |
| Lie et al., 1998 | No difference | NR | Systemic diseases, AB- 6 months |
| Machion et al., 2004 | CAL | No | Systemic diseases, Local/Systemic AB -6 months Prophylactic AB, Allergy, Ongoing drug Rx. |
| Machion et al., 2006 | CAL | No | Systemic diseases, Local/Systemic AB -6 months Prophylactic AB, allergy, Ongoing therapy |
| Meinberg et al., 2002 | PPD | NR | Drugs affecting the perio. status, Pregnant/lactation |
| Newman et al., 1994; Wilson et al., 1997 | PPD, CAL, BOP | No | Systemic diseases (DM), Systemic AB - 3 months, AB premedication, Candidiasis, Perio. Surgery- 4 months, Allergy, Pregnant/lactation, Taking 0.12% CHX MW. |
| OPI (103A) | PPD | Test: periodontitis, tooth sensitivity, tooth caries, infection, dental pain, gingivitis, headache, stomatitis, flu syndrome, dental infection, accidental injury, Vehicle: same AE as test | NR |
| OPI (103B) | PPD |  |  |
| Oringer et al., 2002 | PPD | No | NR |
| Reddy et al., 2016 | No difference | NR | Aggressive Periodontitis, Systemic AB-2 months, Allergy, Perio. surgery |
| Singh et al., 2014 | PPD, CAL | No | Systemic diseases, AB premedication, Allergy, Prosthesis |
| Soeroso et al., 2017 | No difference | NR | AB – 3 months, Systemic diseases, Allergy, Pregnant/lactation |
| Sweatha et al., 2015 | PPD, CAL, PI, GI, BOP | No | Systemic diseases, Pregnant/lactation, Allergy, AB-2 weeks before, Perio. surgery, Long-term drugs implicated in perio. conditions |
| Tabenski et al., 2017 | No difference | No | Heavy smokers, Uncontrolled medical conditions, Systemic AB – 3 months, Pregnant/lactation, Aggressive periodontitis |
| Timmerman et al., 1996 | No difference | No | AB – 3 months, Anti-inflammatory drugs, Mouth rinses |
| Tomasi et al., 2008; Tomasi & Wennstrom, 2011 | No difference | 4 took pain killers, 4 in each group had chewing discomfort due to root sensitivity | Systemic diseases, AB premedication, Pregnant/lactation, Ongoing drug Rx. Implicated, Allergy |
| Tonetti et al., 1998 | No difference | Periodontal abscess in 7 (2 in control and 5 in test) | Pregnant/lactation, AB premedication, Systemic AB – 3 months, Allergy, Systemic diseases, Oral candidiasis, Perio. Surgery – 6 months. |
| Tonetti et al., 2012 | PPD, CAL – 3 m | T- 34pts – 56ADR. C -49 pts – 75 ADR. Only 3 related to the drug. | Allergy, Systemic/local AB – 3 months, Alcohol, Drug abuse, HIV, |
| Van Dyke et al., 2002 | PPD, CAL | T- black hairy tongue -1, rhinitis -1 C- myalgia-1, granulomatous lesion-1 | NR |
| Van Steenberghe et al., 1999 | PPD, CAL, BI | T-8 (15%) gingival abscess-3; redness of site-3 C -14 (27% redness of site | AB – 3 months, Chronic use of drugs with anti-inflammatory properties, MW |
| Williams et al., 2001 | PPD | T- 68.3%;C-62.4%; C-veh-71.9%; Headache, dental infection, increased periodontitis, tooth sensitivity, tooth caries, dental pain, gingivitis, stomatitis | Pregnant/lactation, AB – 3 months, Allergy, Chronic use of drugs implicating the perio. condition |
| Wong et al., 1998; Wong et al., 1999 | No difference | NR | Systemic diseases (DM), Pregnant/lactation, Allergy, AB premedication-3 months, Routine mouth rinses, Smoking >2 packs/day |
| Zingale et al., 2012 | No difference | No | Allergies, Pregnant/lactation, Systemic diseases, AB prophylaxis, AB – 3 months, Drugs on regular basis – OCP, anticonvulsants, anti-coagulants, NSAIDS |

AB, antibiotics; ADR, adverse drug reaction; AE, adverse events; BI, bleeding index; BOP, bleeding on probing; CAL, clinical attachment level; CHX, chlorhexidine; DM, diabetes mellitus; GI, gingival index; GBI, gingival bleeding index; HIV, human immunodeficiency virus; MW, mouth wash; NR, not reported; NSAIDS, non-steroidal anti-inflammatory drugs; OCP, oral contraceptive pills; Perio., periodontal; PI, plaque index; PPD, probing depth; pt, patient; Rx, treatment.

Supplementary Table 11: Results of Egger’s Test and Trim and Fill method for Analysis of Risk of Bias across Studies

|  | **Egger's Test** | | | **Trim & Fill** | | | |
| --- | --- | --- | --- | --- | --- | --- | --- |
|  | **Intercept** | **P value** | **Bias Indicator** | **Adjusted Effect Size** | **Lower CI** | **Upper CI** | **Studies Added** |
| **6-9 months** | | | | | | | |
| **PPD** | 0.486 | 0.001 | Possible Bias | 0.397 | 0.282 | 0.513 | 9 |
| **CAL** | 0.309 | 0.001 | Possible Bias | 0.189 | 0.056 | 0.321 | 9 |
| **BOP** | 0.284 | 0.100 | No Significant Bias | 0.246 | -0.074 | 0.567 | 2 |
| **PI** | 0.082 | 0.393 | No Significant Bias | No Adjustment Needed | -0.106 | 0.270 | 0 |
| **GI** | 0.147 | 0.079 | No Significant Bias | No Adjustment Needed | -0.017 | 0.312 | 0 |
| **12+ months** | | | | | | | |
| **PPD** | 0.419 | 0.001 | Possible Bias | 0.587 | 0.421 | 0.753 | 7 |
| **CAL** | 0.319 | 0.003 | Possible Bias | No Adjustment Needed | 0.110 | 0.529 | 0 |
| **BOP** | 0.312 | 0.099 | No Significant Bias | No Adjustment Needed | -0.059 | 0.682 | 0 |
| **PI** | 0.112 | 0.402 | No Significant Bias | No Adjustment Needed | -0.150 | 0.375 | 0 |
| **GI** | 0.160 | 0.173 | No Significant Bias | 0.149 | -0.075 | 0.373 | 1 |

BOP, bleeding on probing; CAL, clinical attachment level; CI, confidence interval; GI, gingival index; PI, plaque index; PPD, probing pocket depth.
