# Supplementary material for: The long-term efficacy of tetracycline class antimicrobials as local adjuncts in the treatment of chronic periodontitis: a systematic review and meta-analysis
Source: Front Dent Med. 2025 Sep 26;6:1658720. doi: 10.3389/fdmed.2025.1658720 (PMC12511111; doi:10.3389/fdmed.2025.1658720)
Supplement: Supplementary file 3 [file Datasheet2.docx]

## Supplementary Figures


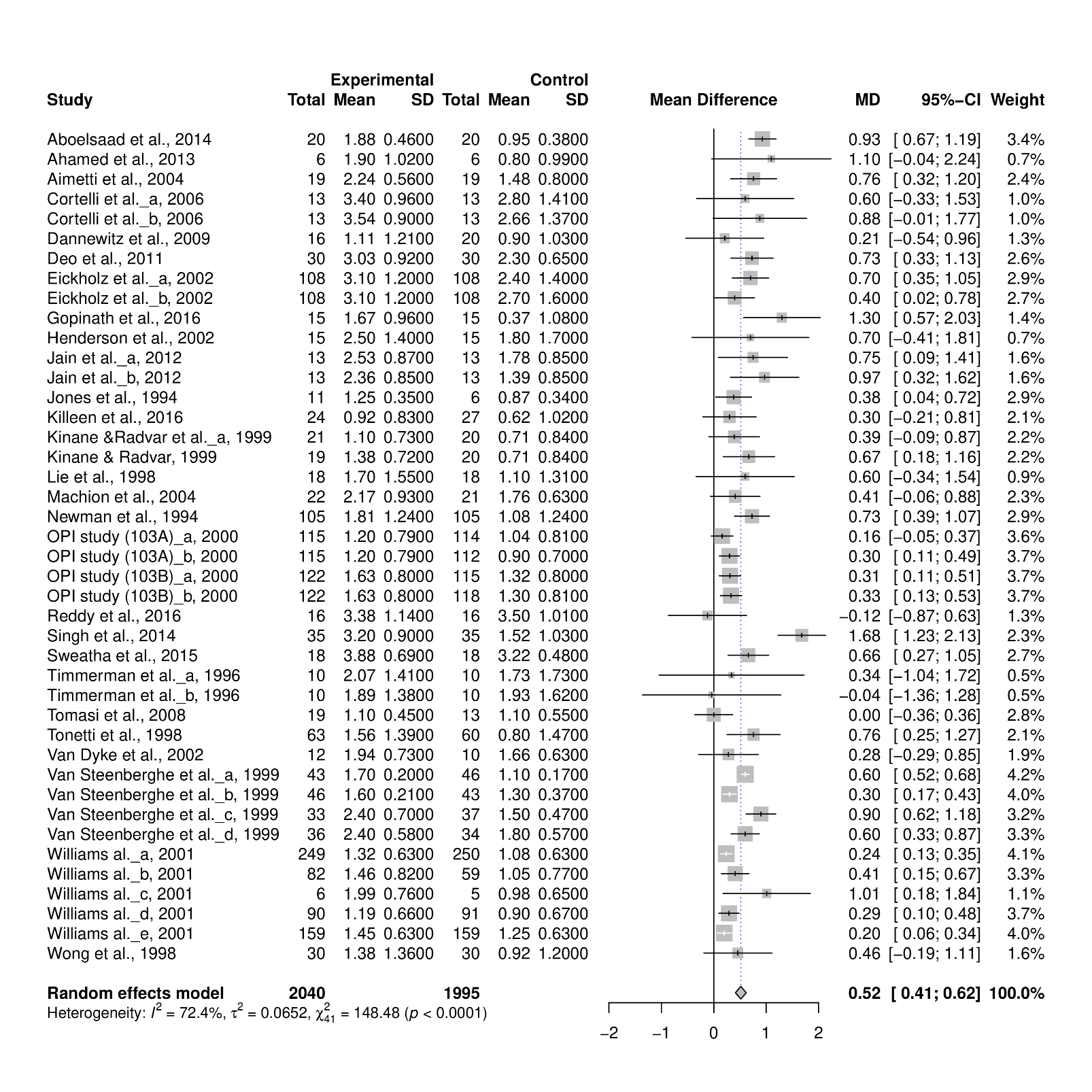


**Supplementary Figure 1A**. Forest plot showing the effect sizes and 95% confidence intervals for PPD reduction across all the studies in 6-9 months. The central markers represent point estimates, and horizontal lines indicate 95% confidence intervals. The blue vertical line denotes the null effect of random-effect model.


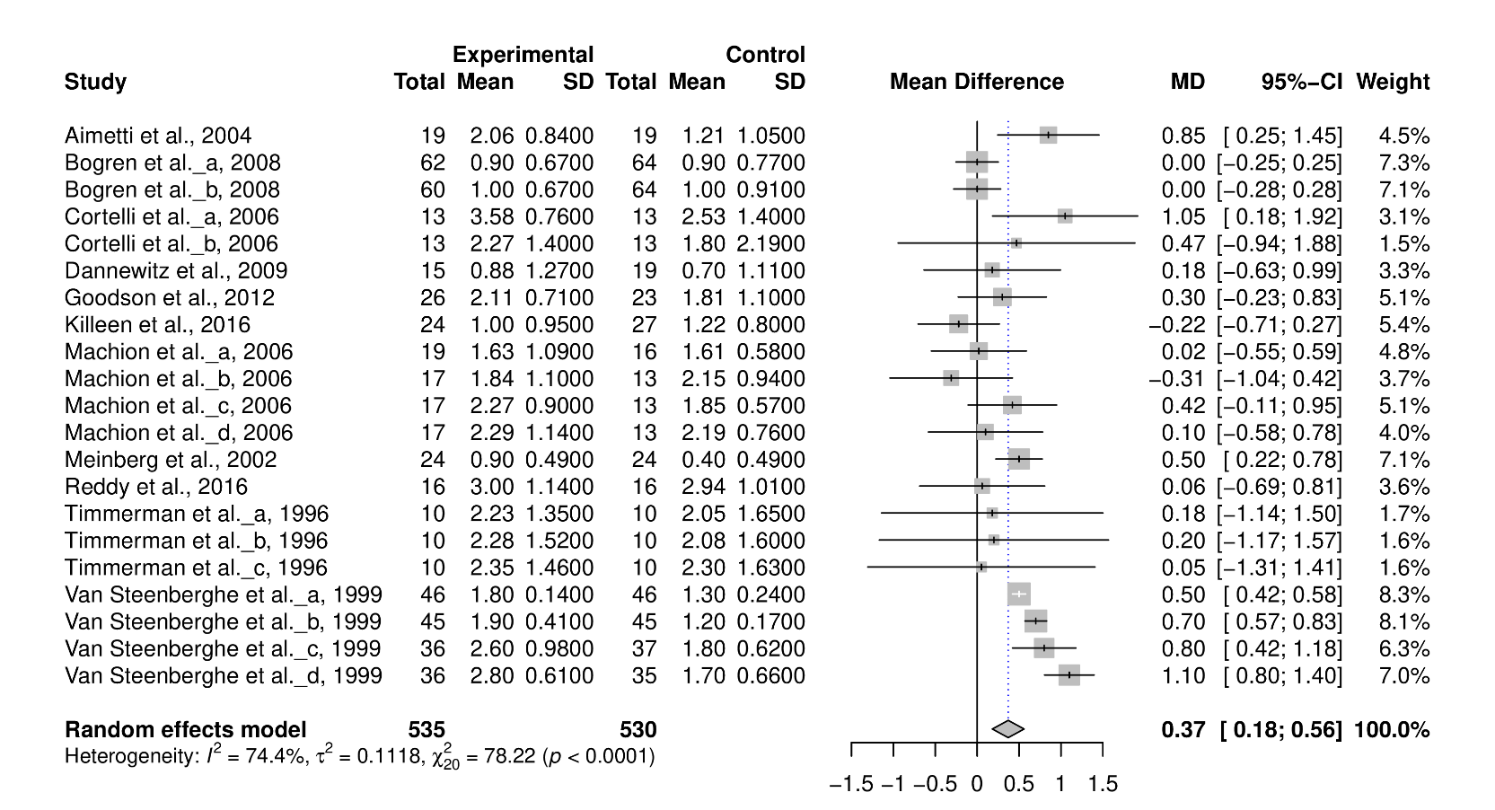


**Supplementary Figure 1B**. Forest plot showing the effect sizes and 95% confidence intervals for PPD reduction across all the studies in 12+ months. The central markers represent point estimates, and horizontal lines indicate 95% confidence intervals. The blue vertical line denotes the null effect of random-effect model.


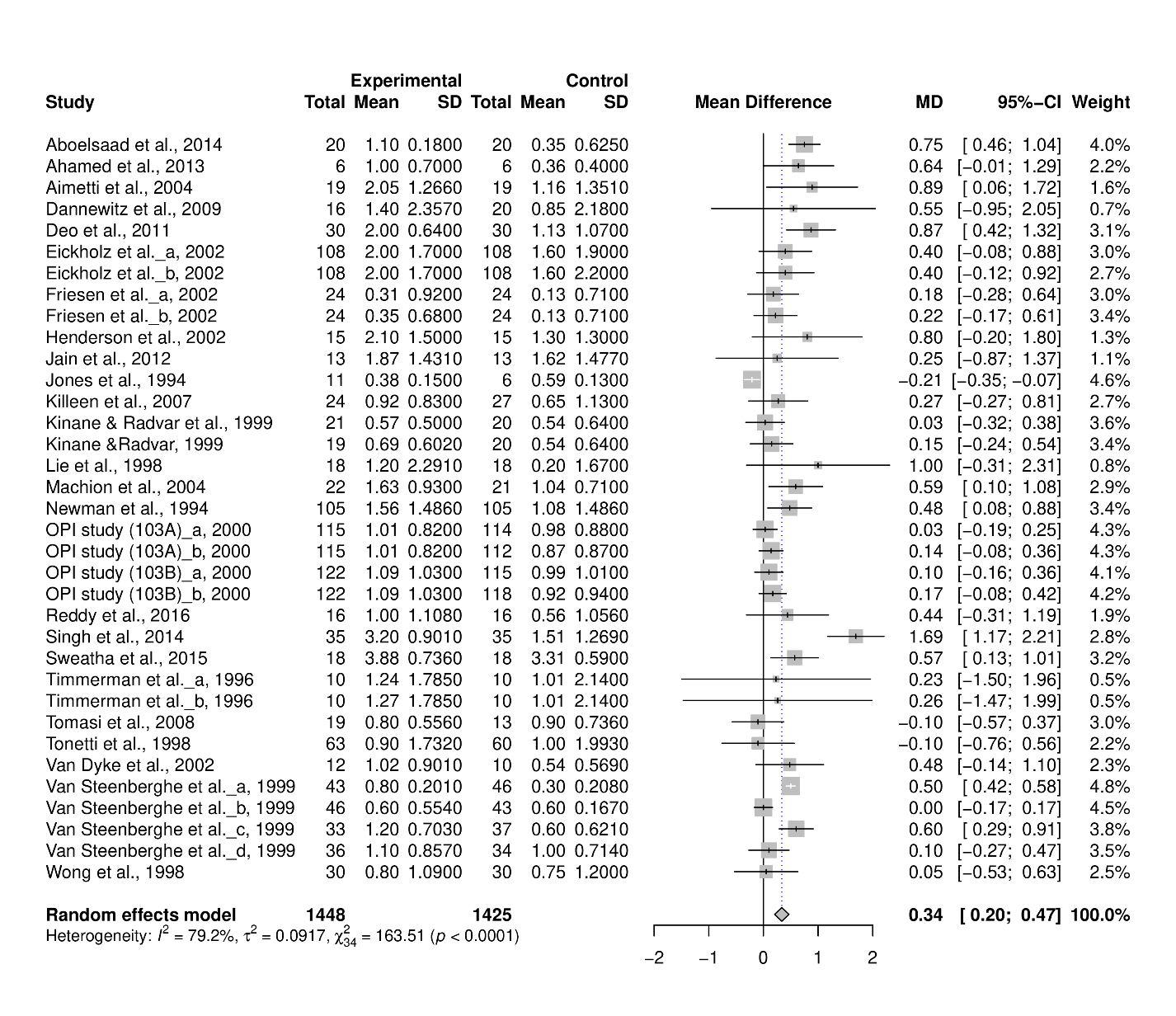


**Supplementary Figure 2A**. Forest plot showing the effect sizes and 95% confidence intervals for CAL gain across all the studies in 6-9 months. The central markers represent point estimates; horizontal lines indicate 95% confidence intervals. The blue vertical line denotes the null effect of random-effect model.


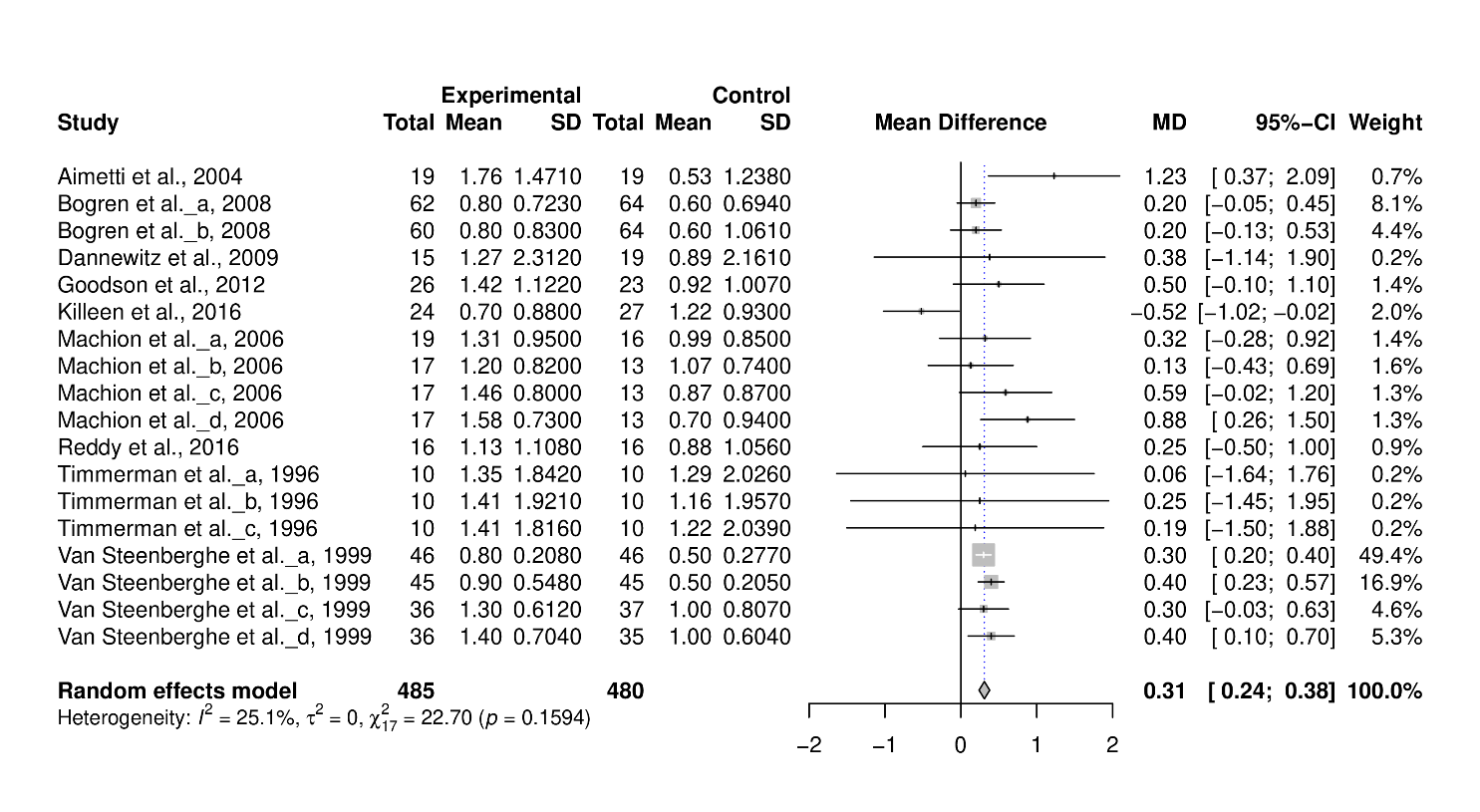


**Supplementary Figure 2B**. Forest plot showing the effect sizes and 95% confidence intervals for CAL gain across all the studies in 12+ months. The central markers represent point estimates; horizontal lines indicate 95% confidence intervals. The blue vertical line denotes the null effect of random-effect model.


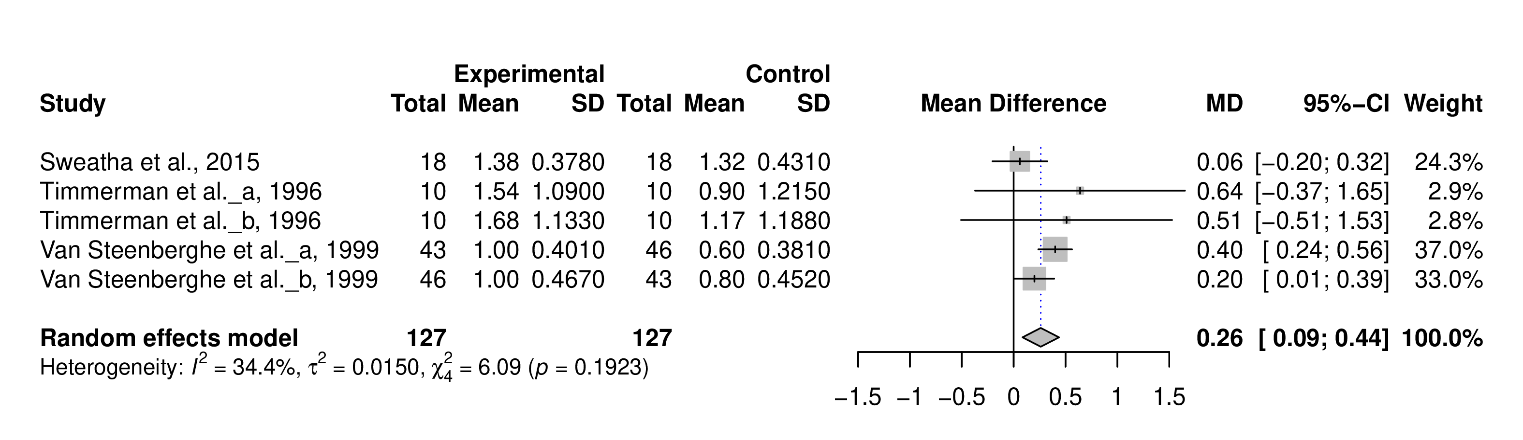


**Supplementary Figure 3A**. Forest plot showing the effect sizes and 95% confidence intervals for BOP change across all the studies in 6-9 months. The central markers represent point estimates; horizontal lines indicate 95% confidence intervals. The blue vertical line denotes the null effect of the random-effect model


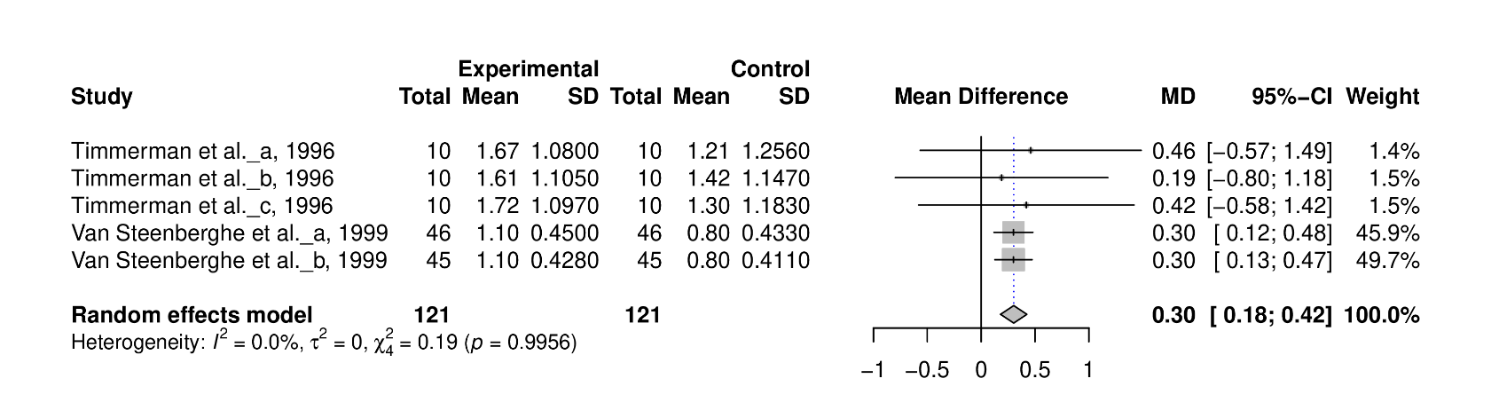


**Supplementary Figure 3B**. Forest plot showing the effect sizes and 95% confidence intervals for BOP change across all the studies in 12+ months. The central markers represent point estimates; horizontal lines indicate 95% confidence intervals. The blue vertical line denotes the null effect of the random-effect model

.


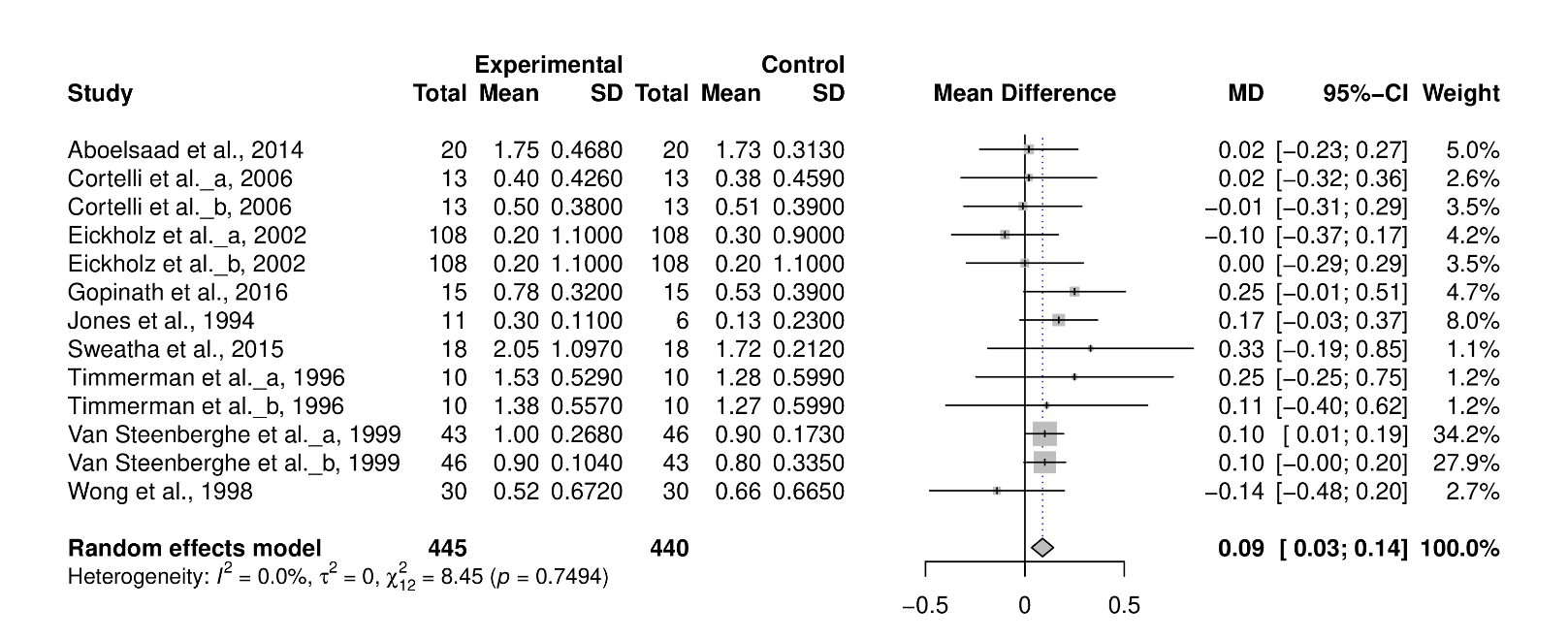


**Supplementary Figure 4A**. Forest plot showing the effect sizes and 95% confidence intervals for PI change across all the studies in 6-9 months. The central markers represent point estimates; horizontal lines indicate 95% confidence intervals. The blue vertical line denotes the null effect of random-effect model.


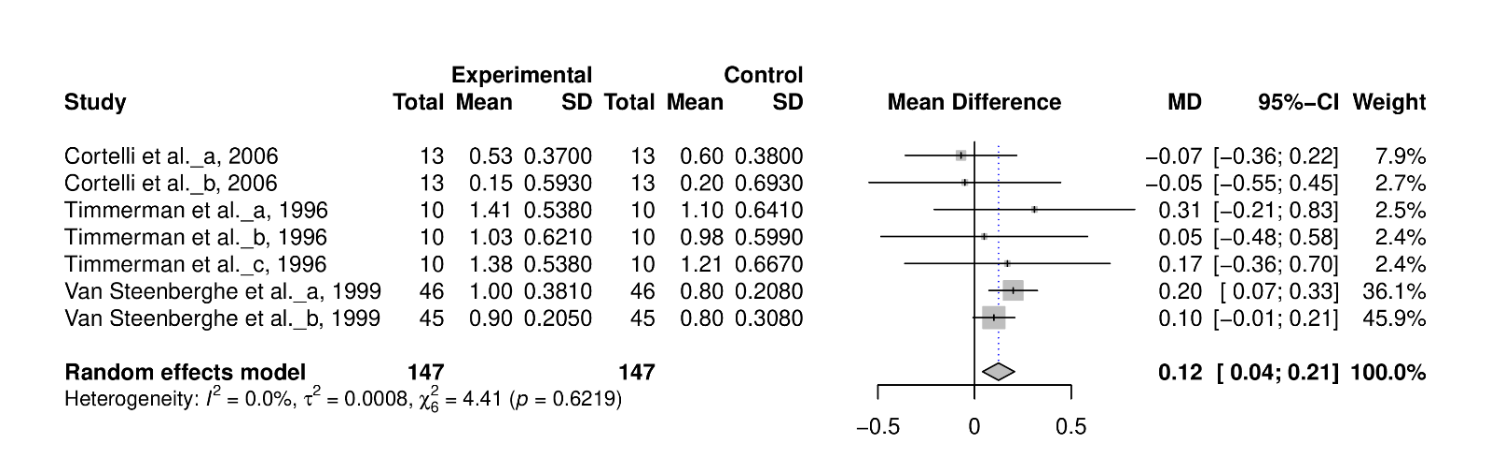


**Supplementary Figure 4B**. Forest plot showing the effect sizes and 95% confidence intervals for PI change across all the studies in 12+ months. The central markers represent point estimates; horizontal lines indicate 95% confidence intervals. The blue vertical line denotes the null effect of random-effect model.


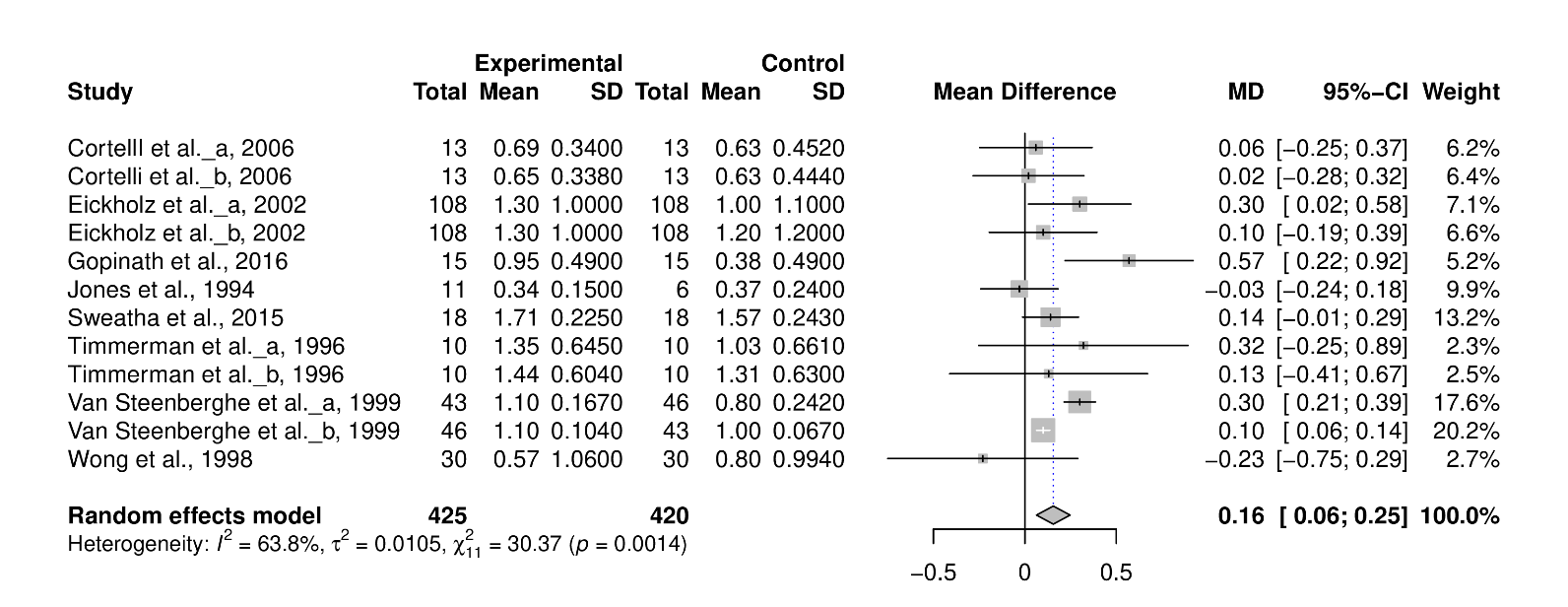


**Supplementary Figure 5A**. Forest plot showing the effect sizes and 95% confidence intervals for GI change across all the studies in 6-9 months. The central markers represent point estimates; horizontal lines indicate 95% confidence intervals. The blue vertical line denotes the null effect of random-effect model. GI, gingival index


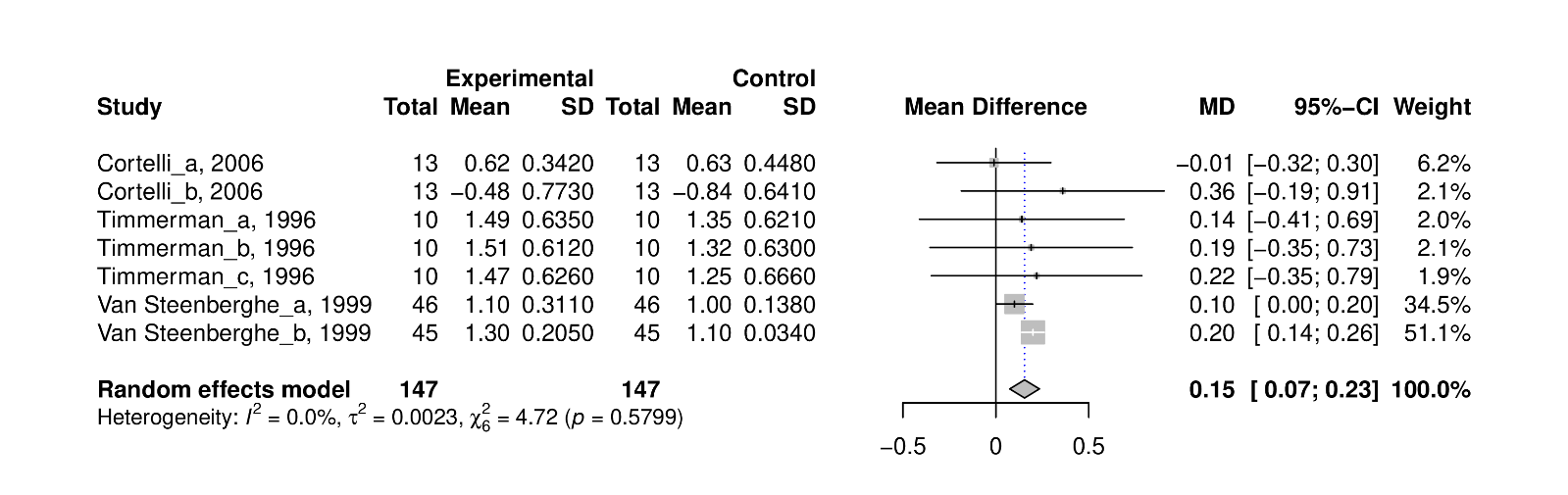


**Supplementary Figure 5B**. Forest plot showing the effect sizes and 95% confidence intervals for GI change across all the studies in 12+ months. The central markers represent point estimates; horizontal lines indicate 95% confidence intervals. The blue vertical line denotes the null effect of random-effect model. GI, gingival index

**(A)**

**
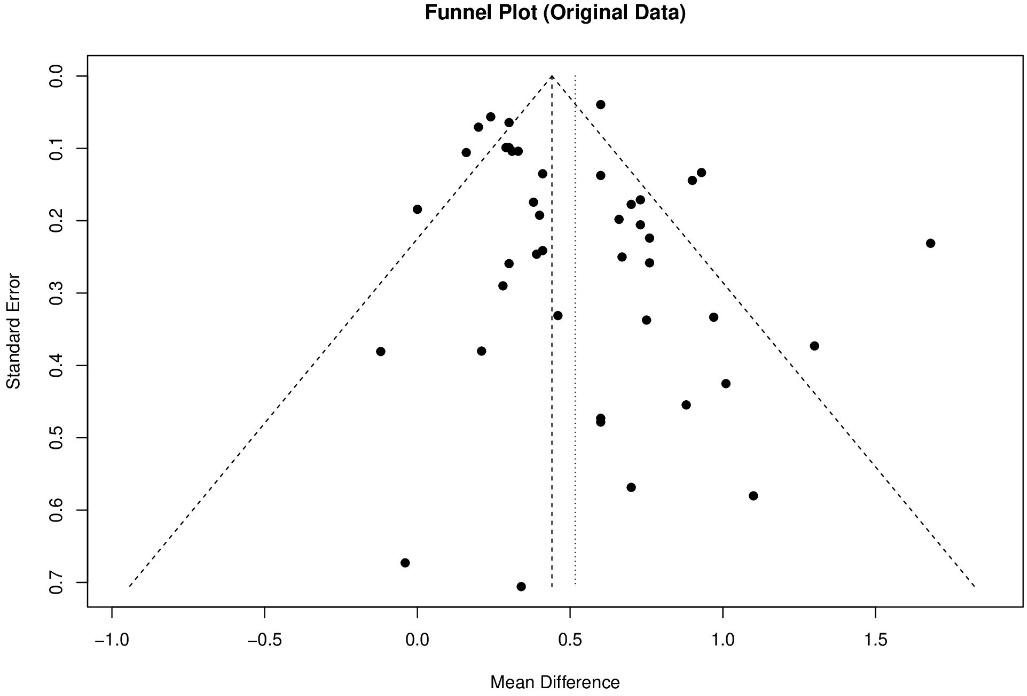
(B)**

**
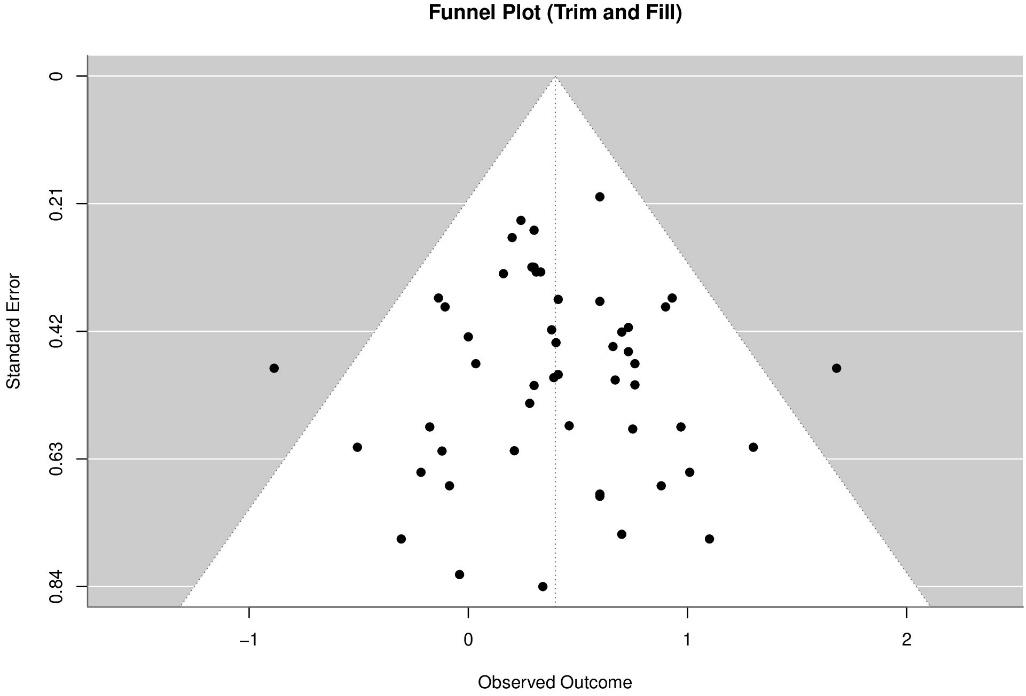
**

**Supplementary Figure 6.** Funnel plots evaluating publication bias in studies reporting probing pocket depth (PPD) reduction in 6-9 months. **(A)** Funnel plot of the original data. **(B)** Funnel plot after applying the trim-and- fill method, with imputed studies added to correct for asymmetry and estimate an adjusted effect size. Dotted lines represent the expected distribution of studies in the absence of bias.

**(A)**

#
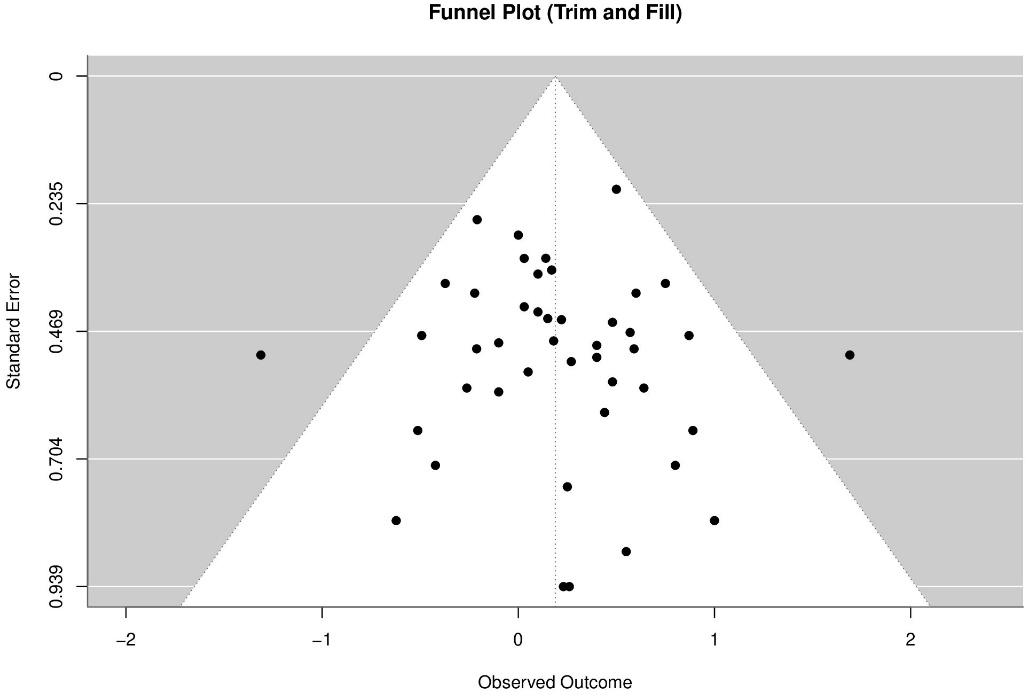

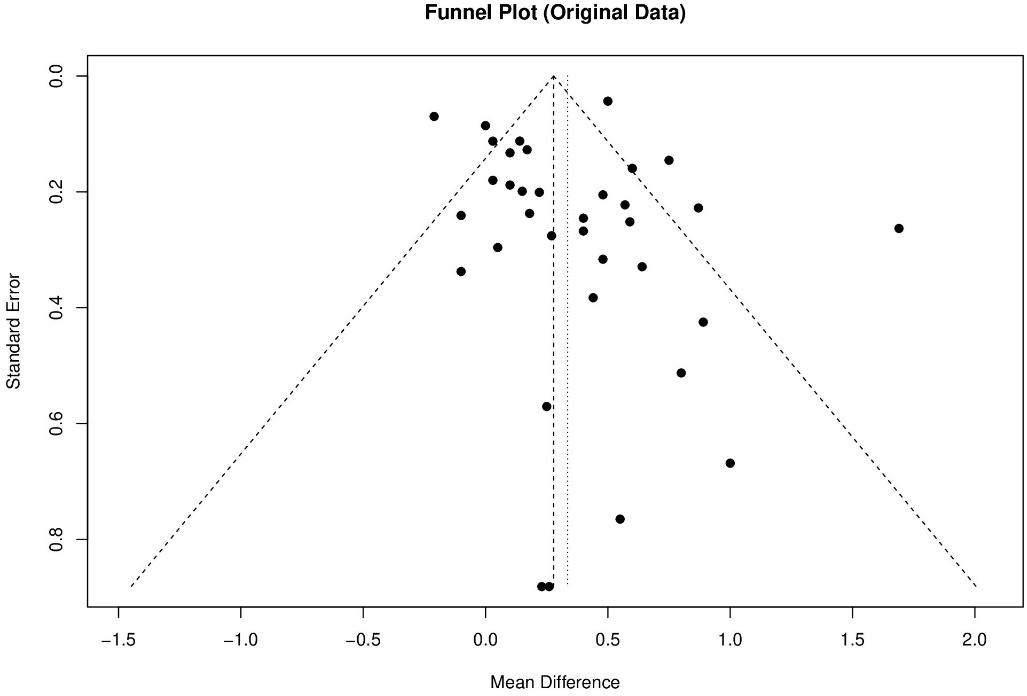
(B)

**Supplementary Figure 7.** Funnel plots evaluating publication bias in studies reporting clinical attachment gain (CAL) in 6- 9 months. **(A)** Funnel plot of the original data. **(B)** Funnel plot after applying the trim-and-fill method, with imputed studies added to correct for asymmetry and estimate an adjusted effect size. Dotted lines represent the expected distribution of studies in the absence of bias.


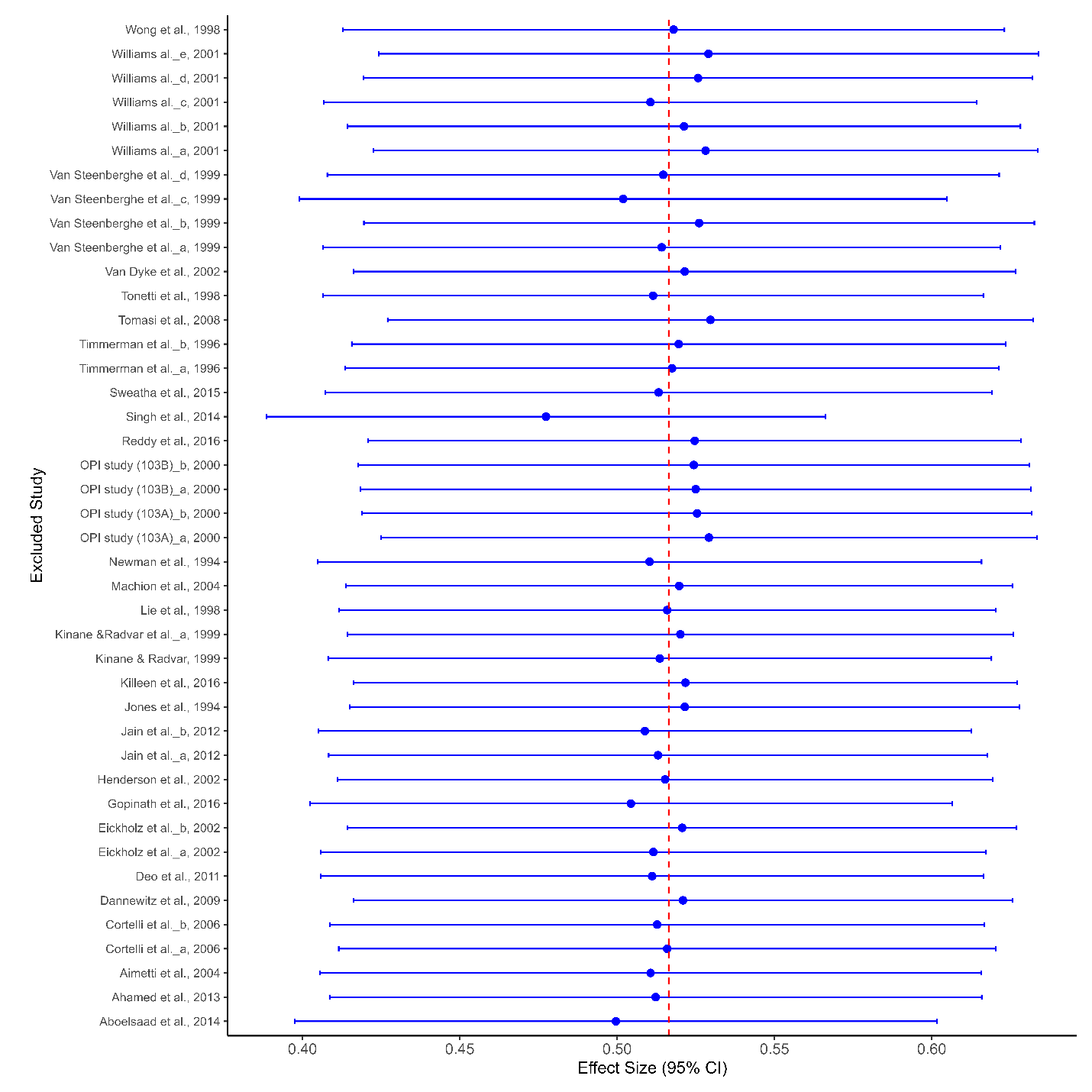


**Supplementary Figure 8A**. Leave‑one‑out sensitivity analysis of the pooled effect size for PPD reduction in 6-9 months. Each point on the plot represents the recalculated overall estimate (with 95% CI) after omitting one study in turn; the red horizontal line shows the original pooled effect.


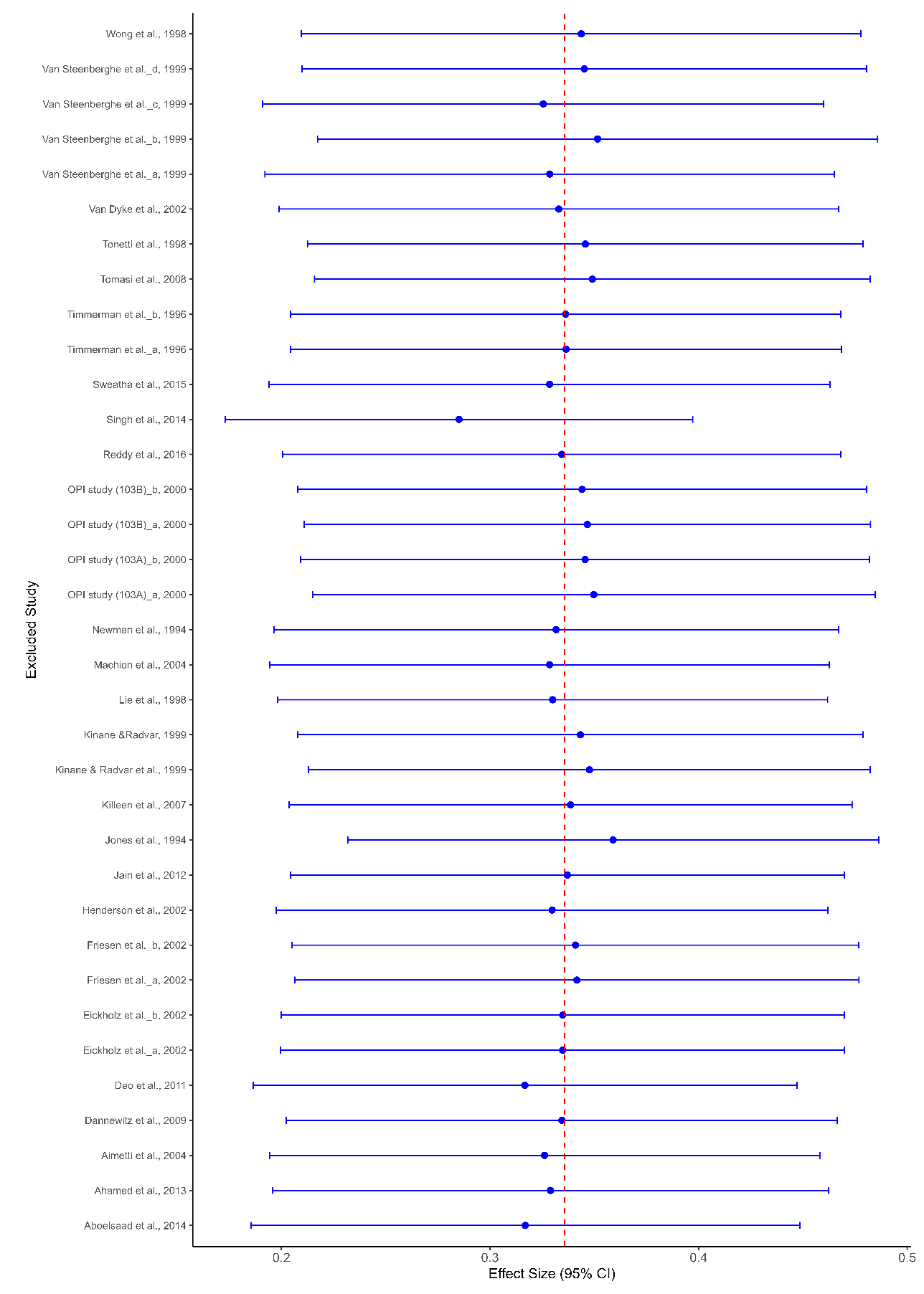


**Supplementary Figure 8B**. Leave‑one‑out sensitivity analysis of the pooled effect size for CAL gain in 6-9 months. Each point on the plot represents the recalculated overall estimate (with 95% CI) after omitting one study in turn; the red horizontal line shows the original pooled effect.
